# Fluorinated synthetic anion carriers: experimental and computational insights into transmembrane chloride transport†
†Electronic supplementary information (ESI) available: Compound synthesis, vesicle-based anion transport studies, NMR titrations, computational studies and anionophore-mediated anion transport in cells. CCDC 1859132. For ESI and crystallographic data in CIF or other electronic format see DOI: 10.1039/c8sc05155k


**DOI:** 10.1039/c8sc05155k

**Published:** 2018-12-14

**Authors:** Michael J. Spooner, Hongyu Li, Igor Marques, Pedro M. R. Costa, Xin Wu, Ethan N. W. Howe, Nathalie Busschaert, Stephen J. Moore, Mark E. Light, David N. Sheppard, Vítor Félix, Philip A. Gale

**Affiliations:** a Chemistry , University of Southampton , Southampton SO17 1BJ , UK; b School of Physiology, Pharmacology and Neuroscience , University of Bristol , Biomedical Sciences Building, University Walk , Bristol BS8 1TD , UK . Email: d.n.sheppard@bristol.ac.uk; c Department of Chemistry , CICECO – Aveiro Institute of Materials , University of Aveiro , 3810-193 , Aveiro , Portugal . Email: vitor.felix@ua.pt; d School of Chemistry , The University of Sydney , NSW 2006 , Australia . Email: philip.gale@sydney.edu.au

## Abstract

A series of fluorinated tripodal tris-thioureas function as highly active anion transporters across lipid bilayers and cell membranes. Here, we investigate their mechanism of action using anion transport assays in cells and synthetic vesicles and molecular modelling of transporter–lipid interactions. When compared with non-fluorinated analogues, fluorinated compounds demonstrate a different mechanism of membrane transport because the free transporter cannot effectively diffuse through the membrane. As a result, in H^+^/Cl^–^ cotransport assays, fluorinated transporters require the presence of oleic acid to form anionic oleate complexes for recycling of the transporter, whereas non-fluorinated analogues readily diffuse through the membrane as free transporters and show synergistic transport with the proton transporter gramicidin. Molecular dynamics simulations revealed markedly stronger transporter–lipid interactions for fluorinated compounds compared with non-fluorinated analogues and hence, higher energy barriers for fluorinated compounds to cross the membrane as free transporters. With use of appropriate proton transporters to ensure measurement of the correct rate-limiting steps, the transport rates determined in synthetic vesicle assays show excellent agreement with the anion transport rates determined in cell-based assays. We conclude that integration of computational and experimental methods provides a strategy to optimise transmembrane anion transporter design for biomedical applications.

## Abstract 2

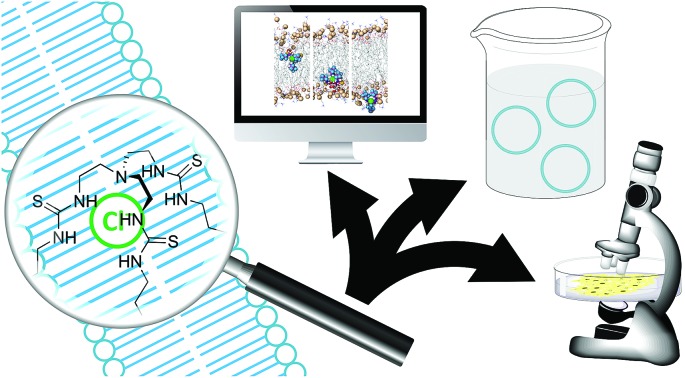
A series of fluorinated tripodal tris-thioureas function as highly active anion transporters across lipid bilayers and cell membranes.

## Introduction

The design, synthesis and investigation of small synthetic transmembrane anion transporters (anionophores) is an important focus of supramolecular chemistry research due to the potential biomedical application of these compounds as research tools and innovative therapies for genetic diseases caused by loss of anion transport.^1^ Numerous classes of anionophores have now been reported^2^ and structure–activity studies conducted to optimise transporter design. These have included analysis of substituent effects,^3^ defining the principle of lipophilic balance,^4^ tuning the length of alkyl tails to enhance transporter activity^5^ and anion encapsulation to confer Cl^–^ selectivity.^6^

Fluorination of therapeutically active compounds is a commonly used strategy in medicinal chemistry to modulate lipophilicity, acidity, conformation and pharmacokinetic properties or to tune reactivity.^7^ The ability to tune properties such as lipophilicity with small structural modifications is desirable in transmembrane transport, given the well-established requirement to optimise the log *P* of a transporter scaffold to balance its affinity for different regions of the membrane.^8^

In the development of highly active anion transporters, addition of trifluoromethyl or fluorine substituents has proven to be an effective strategy. This has been attributed to a combination of increased lipophilicity and anion binding strength.^9^ For example, a CF_3_-containing *o*-phenylenediamine-bisurea was developed as a small-molecule bicarbonate transporter that out-performs the natural anionophore prodigiosin in vesicle tests.^10^ It has also been reported that replacement of a linear hydrocarbon chain by a fluorocarbon chain led to increased Cl^–^/NO_3_^–^ exchange efficacy of the monoacylglycerol class of transporters.^11^ To our knowledge however, there has been little systematic study of the effects of fluoroalkyl substituents. Therefore, the precise effects of alkyl fluorination on anionophore mechanism and interaction with the lipid bilayer are currently unknown.

Due to the highly polarised nature of the C–F bond,^12^ which stems from the high electronegativity of fluorine, perfluorinated compounds are strongly non-polarisable.^13^ Thus, polyfluorinated compounds offer unique amphiphobic properties, such as insolubility in both polar and organic solvents, which has been exploited in the separation of complex reaction mixtures.^14^ With new tools available to elucidate anionophore mechanism of action^6^ and the ability to monitor anion transport by anionophores in living cells,^15^ we investigated the effects of alkyl fluorination on anion transport by synthetic anionophores to determine the physical effects of fluorination on the transport process and activity. To contextualise these observations, a comprehensive molecular dynamics study of the behaviour of the compounds within a model POPC bilayer was also undertaken to identify the structural and energetic origins of the trends observed.

**Fig. 1:**
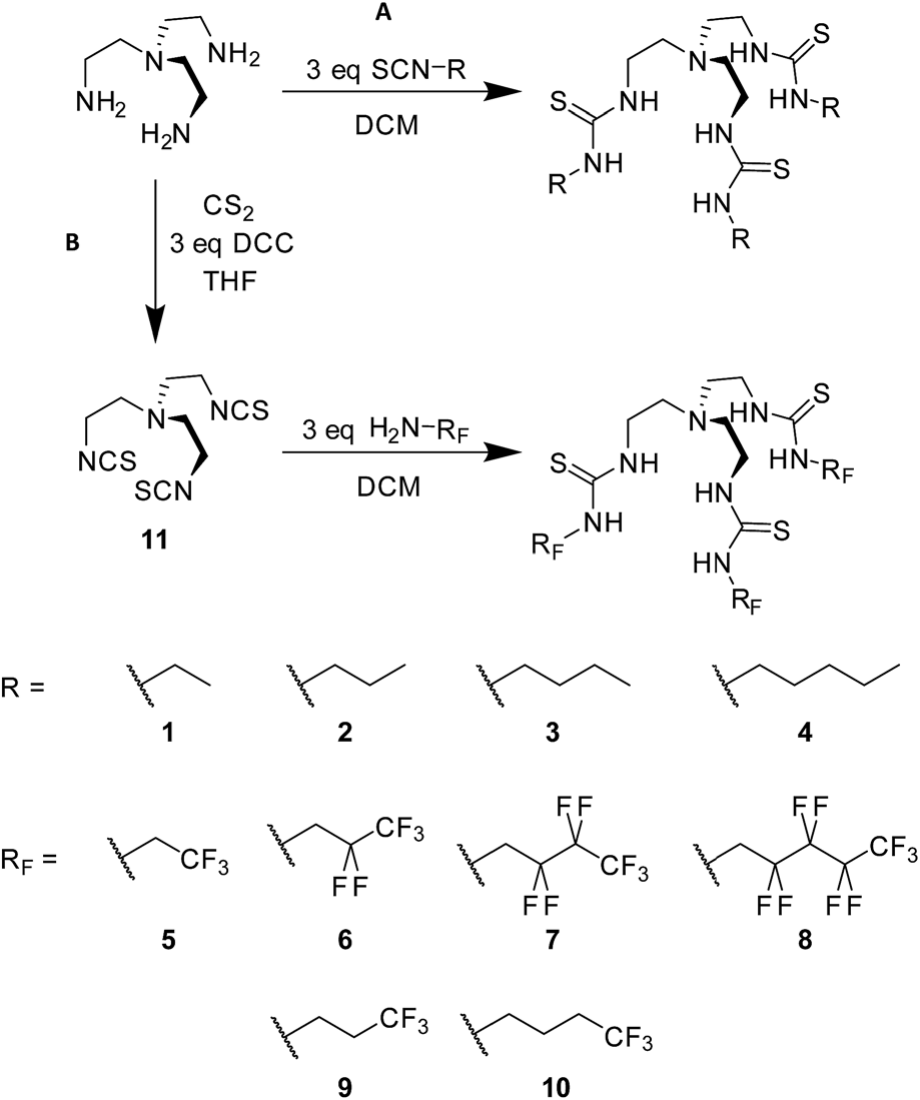
Synthesis and general structure of the tris-thiourea series, (A) alkyl compounds **1–4**, SCN-R = isothiocyanates; (B) compounds **5–10** with fluorinated alkyl (*R*_F_) chains.

For this study, a systematic series of alkyl and fluorous tris-thiourea anion transporters **1–8** (Fig. 1) was designed. Additionally, compounds **9** and **10** were also synthesised with a single terminal CF_3_ group to determine whether the number of C–F bonds mediate any observed effects (Fig. 1).

## Results and discussion

### Synthesis and characterisation

**Fig. 2:**
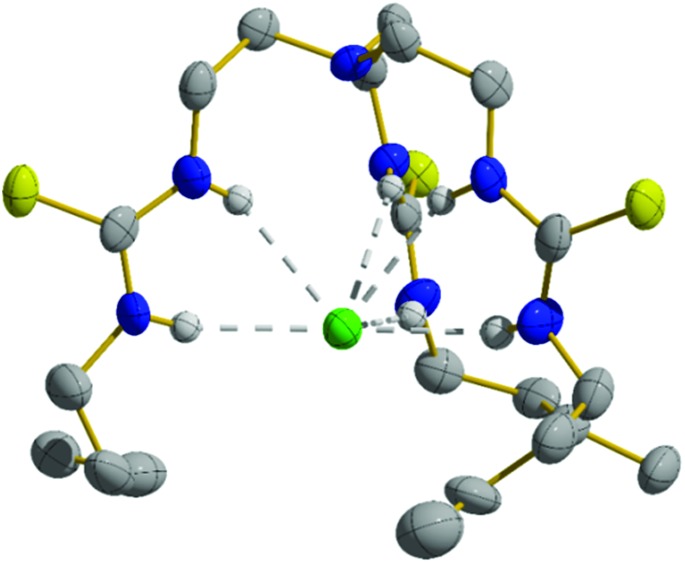
Single crystal structure of the Cl^–^ complex of compound **3** (CCDC ; 1859132 †). Thermal ellipsoids drawn at the 50% probability level. Only one tripodal complex from the asymmetric unit is shown while TBA^+^ counterions and most hydrogen atoms have been omitted for clarity.

Compounds **1–4** were synthesised in a single step from commercially available tris-(2-aminoethyl)amine and the appropriate isothiocyanates; compounds **3** and **4** have been previously reported.^6^,^16^ Compounds **5–10** were synthesised from the appropriate commercially available fluorous alkyl amine and the tris-(isothiocyanatoethyl)amine intermediate **11**, prepared as per the literature procedure (Fig. 1).^17^ As part of this work, an X-ray crystal structure of **3** was obtained, showing Cl^–^ held by six·NH···Cl^–^ hydrogen bonds (Fig. 2). Full experimental details and characterisation data are available in Section 2 of ESI.†

The lipophilicity of compounds **1–10** was evaluated by determining the experimental retention factor (*k*′) calculated from the measured retention of the compounds on a C_18_ RP-HPLC column after isocratic elution with 50% CH_3_CN/H_2_O. Under these conditions, log(*k*′) is proportional to log *P*,^18^ and these values are tabulated in Table 1. Perfluorination of the alkyl chains resulted in a large increase in lipophilicity (*e.g.* increase of 1.06 units in log(*k*′) from compound **3** (butyl) to **7** (perfluorinated butyl)). By contrast, the increase in lipophilicity on the introduction of a single CF_3_ substitution was very modest (increase of 0.11 units between **3** and **10**), as has been noted in previous studies.^7^,^13^,^19^

**Table 1:** Selected data for the tris-thiourea series

	Log(*k*′)^*a*^	Cl^–^*K*_a_^*b*^ (M^–1^)	*V*_S,max_ ^*c*^ (kcal mol^–1^)
**1**	–1.82	557	82.03
**2**	–0.87	677	81.72
**3**	–0.14	596	81.34
**4**	0.45	648	81.35
**5**	–0.44	470	92.27
**6**	0.27	565	93.03
**7**	0.92	733	92.76
**8**	1.53	669	92.86
**9**	–0.28	466	90.36
**10**	–0.03	575	91.40

^*a*^Log of the retention factor (*k*′) derived from the retention time on a C_18_ RP-HPLC column measured by elution with 30% MeCN/H_2_O. log(*k*′) ∝ log *P*.^18^

^*b*^Association constants from ^1^H NMR titration with TBACl in 0.5% H_2_O/DMSO-*d*_6_. *K*_a_ obtained by fitting of the chemical shift data to the 1 : 1 binding model using BindFit v0.5.^20^ Asymptotic errors all <3%, details in the ESI.

^*c*^Electrostatic potential maximum estimated as described below (see the Molecular modelling section).

To quantify the Cl^–^ binding affinities of the different compounds, we used ^1^H NMR titration with tetrabutylammonium chloride (TBACl) in 0.5% H_2_O/DMSO-*d*_6_. Binding constants (*K*_a_) were obtained using a global fitting of the chemical shift data^20^ and are reported in Table 1. The results demonstrate relatively strong Cl^–^ binding for all compounds in a competitive solvent environment.

### Transport in synthetic vesicles

**Fig. 3:**
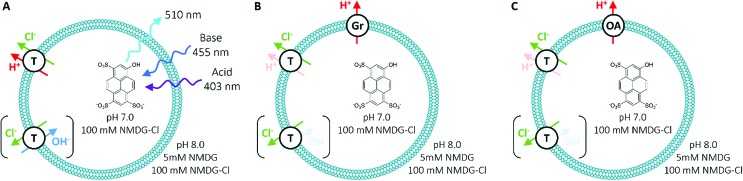
Schematic of the HPTS assay employed for anion transport studies in synthetic vesicles, with (A) no assisting proton transporter, (B) in the presence of 0.1% gramicidin (Gr) and (C) with 2% oleic acid (OA). T = test transporter molecule.

To measure Cl^–^ transport by compounds **1**–**10**, we used a pH-discharge vesicle-based assay (Fig. 3). POPC vesicles loaded with the pH-sensitive dye 8-hydroxypyrene-1,3,6-trisulfonic acid (HPTS) were suspended in a solution of *N*-methyl-d-glucamine (NMDG) chloride, buffered to pH 7.0 with HEPES (10 mM). The test compound was added externally as a solution in DMSO, before the transport assay was commenced by the external addition of the free base NMDG to raise the external pH to 8.0. Anionophores were added at 0.01 mol% loading with respect to lipid.

**Fig. 4:**
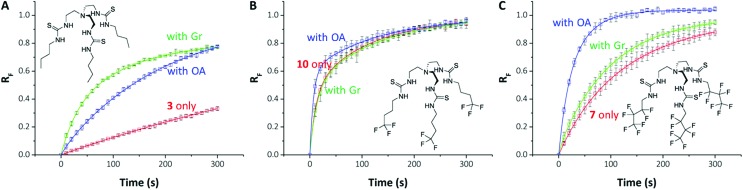
Differential effects of proton transporters on transmembrane Cl^–^ transport by anionophores. (A–C) Show chemical structures and data from HPTS assays for compounds **3** (A), **10** (B) & **7** (C) for transporter (0.01 mol%) only (red); transporter with gramicidin (0.1 mol%) (green) and transporter with oleic acid (2 mol%) (blue). Data are means ± SD (*n* = 3); the continuous lines are the fit of exponential functions to mean data. *R*_F_ = normalised fluorescence ratio.

In this assay, anionophores dissipate the pH gradient generated by external NMDG addition (Fig. 4) by electroneutral co-efflux of HCl, Cl^–^/OH^–^ exchange or a combination of both processes. To record intra-vesicular pH, we monitored the ratio between the fluorescence emissions (at 510 nm) of the acidic (excitation at 403 nm) and basic (excitation at 460 nm) forms of HPTS. At the end of the experiment, data were calibrated to 100% dissipation by lysis of the vesicles with detergent (Triton-X 100 in 7 : 1 water/DMSO). Experiments lasted 5 minutes and were conducted in triplicate. The fluorescence ratio recorded was averaged and fitted with an exponential function to obtain values for the initial rate of transport (see ESI†).

We have demonstrated previously that hydrogen bond-based anion transporters facilitate H^+^ transport by deprotonation of hydrogen bond donor groups, which couple to Cl^–^ transport leading to HCl cotransport and hence, dissipation of pH gradients in pH discharge assays using Cl^–^ containing media.^6^ However, some tripodal thioureas exhibit Cl^–^ over H^+^/OH^–^ selectivity with the result that their transport rates in the pH-discharge assay were limited by slow H^+^/OH^–^ transport. To prevent this rate-limiting effect obscuring the true Cl^–^ transport potency of these compounds, two additional proton transporters were used to accelerate H^+^/OH^–^ transport.^21^ The first proton transporter was the bacterial proton channel gramicidin (Gr, 0.1 mol% with respect to lipid concentration), which allows fast efflux of protons. The second proton transporter was oleic acid (OA, 2 mol%), a naturally occurring fatty acid. Anionophores facilitate fatty acid-mediated H^+^ transport by promoting the transbilayer movement (“flip-flop”) of anionic fatty acids.^22^ Compounds that are rate-limited by their H^+^ or functionally equivalent OH^–^ transport exhibit enhanced transport in the presence of Gr or OA, with Cl^–^ transport coupling to the faster H^+^ efflux *via* the assisting proton transporter. It should be noted that Gr function is equivalent to the widely used FCCP (carbonyl cyanide 4-(trifluoromethoxy)phenylhydrazone), which accelerates H^+^ transport in HPTS assays.^23^ However, the use of both Gr and OA in HPTS assays provided key mechanistic insights that are not accessible using one proton transporter alone, as will be detailed in this section.

**Fig. 5:**
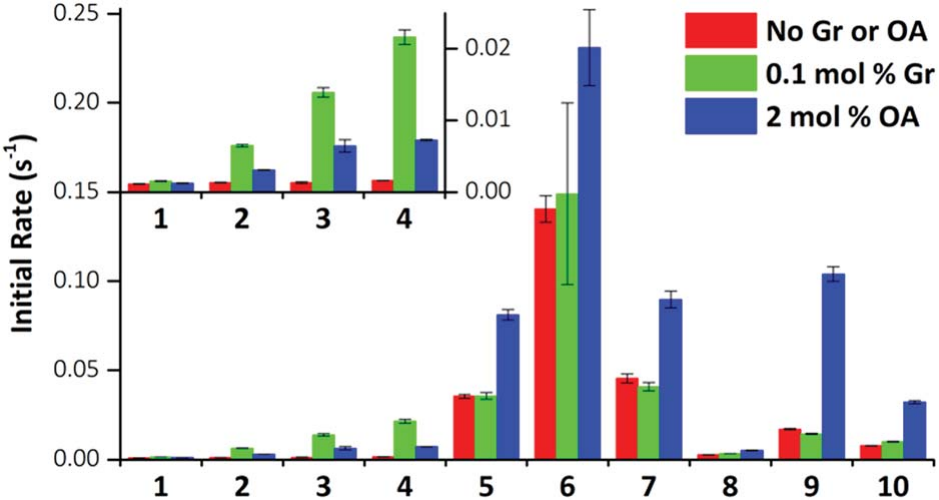
Effect of gramicidin and oleic acid on anionophore-mediated pH gradient dissipation. The initial rate of pH gradient dissipation determined from the HPTS assays for compounds **1–10** are shown (0.01 mol% transporter only, red bars; with 0.1 mol% Gr, green bars; with 2 mol% OA, blue bars). Data are from exponential fits to mean curves ± SEM (*n* = 3); insert: expanded view for compounds **1–4**.

We performed the HPTS assay on all anionophores using three conditions: (i) without any proton transporter, (ii) with Gr and (iii) with OA. The initial rates determined for each compound at 0.01 mol% loading under these conditions are plotted in Fig. 5.

The data demonstrate that fluorination of the tripodal thioureas dramatically improved transmembrane Cl^–^ transport. Except for compound **8** that might be too lipophilic to be effectively delivered into the lipid bilayer,^15^ the fluorinated compounds are all markedly more active than their nonfluorinated analogues and follow the trend of increasing activity with increasing chain length. For example, the most active fluorous compound **6** exhibits an improvement more than an order of magnitude over alkyl analogue **2**. Moreover, compounds **9** and **10** exhibited enhanced activity over their alkyl analogues **2** and **3**, albeit the effect was not as great as compounds with higher degrees of fluorination. These results are further rationalised by our molecular modelling investigations below.

These trends are not simply explained by considering the relationship between the lipophilicity or anion binding ability of the compounds and their transport activity. The data demonstrate no correlation between the initial rate and *K*_a_ and little evidence of the expected parabolic relationship between lipophilicity and the transport rate.8b–d For instance, while compounds **4**, **10** and **6** have comparable lipophilicity, the former ones display low initial rates of pH dissipation, while **6** promotes the fastest efflux of Cl^–^, even in the absence of a proton transporter (Fig. 5).

On close examination of the HPTS assay data a complex picture emerges with differences in the behaviour of fluorinated and non-fluorinated analogues. The non-fluorinated anionophores **1–4** show enhanced pH gradient dissipation with both Gr and OA (Fig. 4A and 5), which can be explained by their Cl^–^ > H^+^/OH^–^ selectivity. These anionophores are not effective H^+^/OH^–^ transporters and therefore a proton transporter (either Gr or OA) is required for net H^+^/Cl^–^ symport to dissipate the pH gradient. The results also demonstrate that for these anionophores, rate enhancement by OA is not as high as by Gr. We interpret these results to suggest that proton efflux mediated by OA coupled to these anionophores is still potentially rate-limiting and not as kinetically fast as with Gr.

**Fig. 6:**
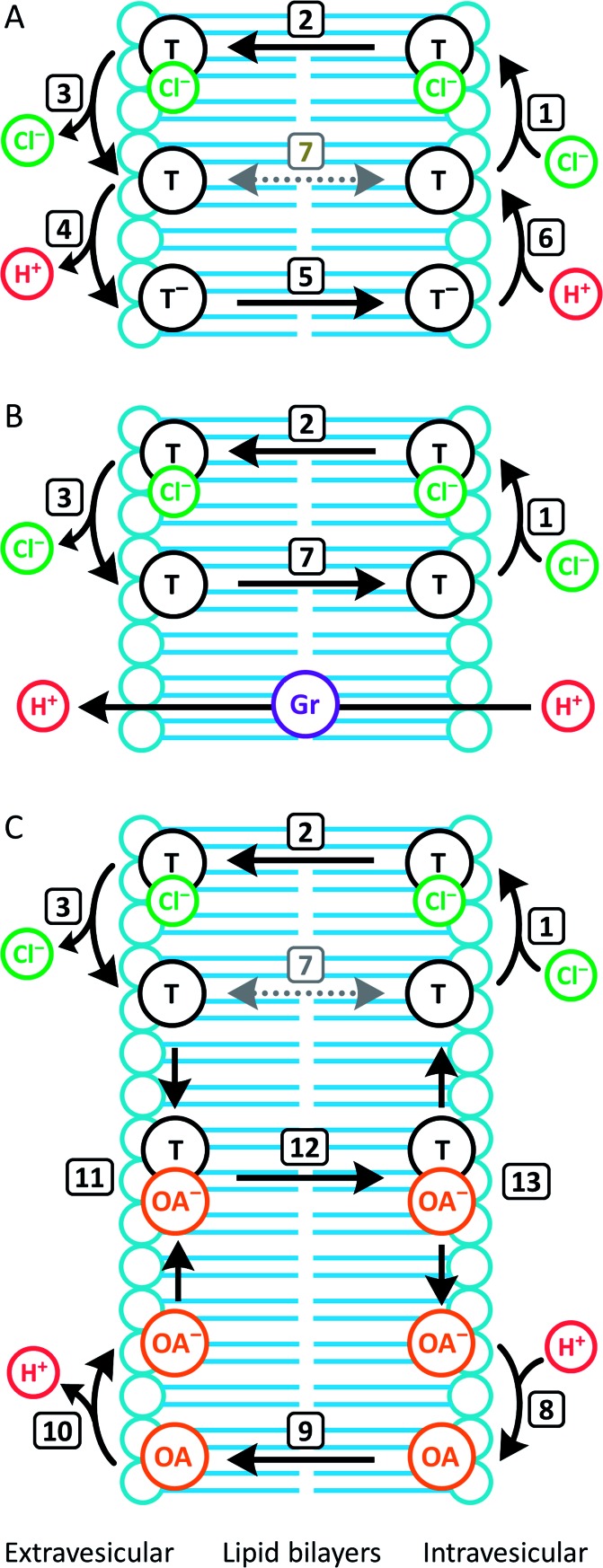
Dissection of transport processes in the HPTS assay under different conditions. Numbered arrows indicate different steps in the transport mechanism. (A) Without Gr or OA, the transporter (T) facilitates H^+^/Cl^–^ cotransport by a mechanism that involves deprotonation/reprotonation and Cl^–^ binding/release. OH^–^ binding/release is a functionally equivalent alternative process for deprotonation/reprotonation. Transmembrane diffusion of the free transporter (step 7) is not required; (B) in the presence of Gr, which facilitates H^+^ uniport, the transporter facilitates synergistic H^+^/Cl^–^ cotransport with Gr by a mechanism that requires step 7. (C) In the presence of OA, the transporter facilitates synergistic H^+^/Cl^–^ cotransport with OA by a mechanism that does not require step 7.

In contrast to **1–4**, the fluorinated compounds **5–10** displayed the surprising behaviour that transmembrane Cl^–^ transport is little affected by Gr, but significantly enhanced by OA (Fig. 4B, C and 5). These results suggest a fundamental difference in transport mechanism between the alkyl compounds **1–4** and the fluorous analogues **5–10**. In the absence of any proton transporters (Fig. 6A), the anionophore requires six steps to dissipate the pH gradient: (1) the anion transporter (T) binds Cl^–^ on the intra-vesicle side of the membrane; (2) translocation of the TCl^–^ complex across the membrane; (3) release of Cl^–^ outside the vesicle; (4) deprotonation (or OH^–^ binding) of T; (5) translocation of deprotonated T^–^ (or OH^–^ complex of T) across the membrane; (6) reprotonation (or OH^–^ release) to form neutral T. In this process, the translocation of the free transporter (step 7) is not required. In the Gr-coupled assay (Fig. 6B), besides steps 1, 2 and 3, the additional step 7 is required for the anionophore to function synergistically with Gr to dissipate the pH gradient. If anionophore-mediated H^+^ or OH^–^ transport (steps 4, 5 and 6) is rate-limiting, the presence of Gr facilitates H^+^ flux and enhances the rate of Cl^–^ transport (as observed in the case of **1–4**), but only under the condition that the translocation of the free transporter (step 7) is fast.

In the presence of OA (Fig. 6C), new pathways to complete the overall transport cycle are introduced. The anionophores can perform steps (1)–(3), which can be considered as a partial Cl^–^ transport process (a complete Cl^–^ transport cycle should include step (7)). OA performs partial H^+^ transport by protonation of the oleate ion at the intravesicular side of the membrane to form neutral OA (step 8) and the neutral OA moves to the extravesicular side (step 9) where it deprotonates (step 10). The oleate ion cannot move across the membrane by itself because of the hydrophilicity of the carboxylate head group and therefore a H^+^ transport cycle cannot be completed with OA alone. In the presence of an anionophore, the oleate ion binds the anionophore (step 11) forming a lipophilic anionic complex that moves back to the intravesicular side (step 12) where the complex dissociates (step 13) allowing the anionophore to re-bind Cl^–^ (step 1) and oleate to re-bind H^+^ (step 8) to initiate the next transport cycle. The overall transport cycle results in net flow of H^+^ and Cl^–^ out of vesicles. In this process, there is no requirement for free anionophore to cross the membrane (step 7) because step 12 serves as the alternative process to recycle the free anionophore. As step 7 is required in synergistic transport with Gr, but not OA, the observation for anionophores **5–10** that pH gradient dissipation was enhanced by OA, but not Gr suggests that step 7 is rate-limiting for **5–10**.

To summarise, data from the HPTS assays suggest that all the compounds tested mediate steps 1–3 for Cl^–^ transport. However, for the fluorinated compounds **5–10**, the free transporter cannot effectively diffuse through the membrane (step 7 prohibited), leading to no synergistic H^+^/Cl^–^ symport with Gr.

### Transport in cells

To investigate anion transport in cells by fluorous anionophores, we used Fischer rat thyroid (FRT) cells, a cell line widely employed to investigate epithelial ion transport.^24^ To study anionophore-mediated anion transport, we used FRT cells engineered to express the halide-sensitive yellow fluorescent protein YFP-H148Q/I152L^15^,24b,^25^ With these cells, we measured indirectly facilitated Cl^–^ transport through the plasma membrane by I^–^ entry into cells coupled to the exit of intracellular Cl^–^, leading to the quenching of YFP fluorescence by I^–^.^15^

**Fig. 7:**
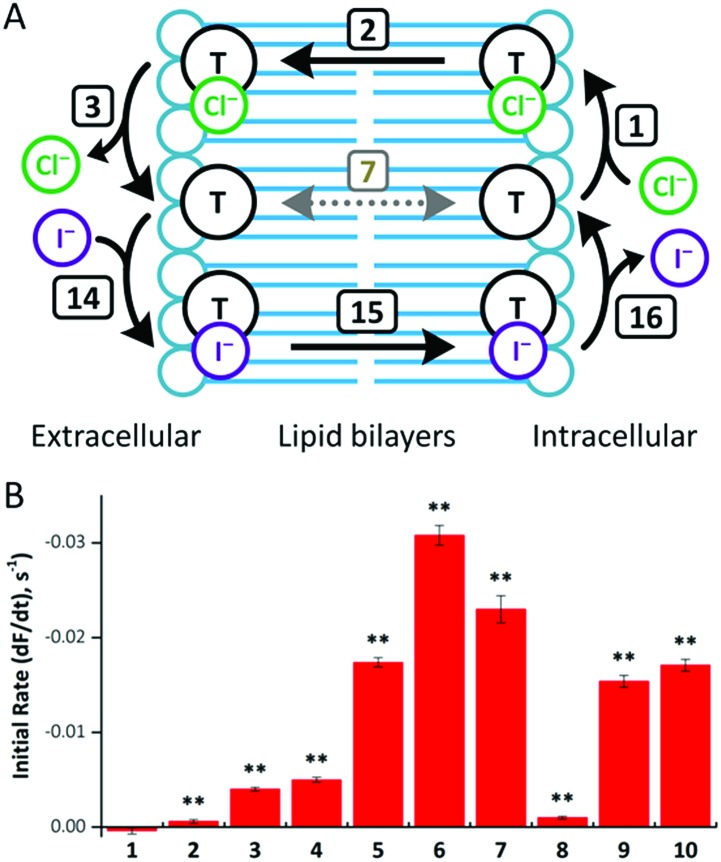
(A) Mechanism of anionophore (T)-mediated Cl^–^/I^–^ exchange. Transmembrane diffusion of the free transporter (step 7) is not required for anion exchange. (B) Anion transport by compounds **1–10** in YFP-FRT cells. Anion transport by the indicated anionophores (50 μM) was determined from the fit of first-order exponential functions to the fluorescence decay elicited by NaI (100 mM). Fluorescence quenching by the anionophore vehicle (DMSO, 0.5% v/v) was subtracted from each anionophore to determine their activity. Data are means ± SEM (*n* = 16–32 from ≥4 independent experiments); **, *P* < 0.01 *vs.* vehicle.

Consistent with our observations using the HPTS assay, fluorous compounds demonstrated higher efficacy than their alkyl counterparts (Fig. 7). Of note, compounds **5** and **6** were more than 40 times more effective than their alkyl counterparts (compounds **1** and **2**, respectively). However, further extension of transporter tail length failed to improve anion transport. For example, compound **7** was only 5 times more effective than compound **3**, while compound **8** was less effective than compound **4** (Fig. 7).

To explore anionophore potency in cells, we determined concentration–response relationships for compounds **5–7**, **9** and **10** (ESI Fig. S4.35†). For compounds **5–7** and **9**, fluorescence decay exhibited noticeable concentration-dependence, whereas compound **10** did not demonstrate activity until tested at 50 μM. Although YFP fluorescence is pH sensitive,^25^ the change in fluorescence achieved with the tested compounds is too large to be explained by a change in intracellular pH and there was no transmembrane pH gradient in these experiments. Thus, the observed fluorescence quenching of YPF resulted from I^–^ entry into the cells instead of a change in intracellular pH.

Of note, the most active transporter, compound **6**, demonstrates a ∼50% improvement in transport rate compared with the previously reported best transporter tested in the same assay.^26^ We also note that the best correlation between vesicle and cell data was achieved when the fastest rate achieved in any of the proton transporter-assisted assays (with Gr or OA) was used (ESI Fig. S4.36†). The YFP-FRT assay measures an exchange process that does not involve free transporter diffusion. As discussed above, in HPTS assays compounds may be limited by different transport processes. It is therefore important to select the conditions that best demonstrate the rate of Cl^–^ transport (steps 1–3). In this way, the correct rate-determining step is selected to avoid underestimating anionophore activity in the HPTS assays.

### Molecular modelling

Using Density Functional Theory (DFT) calculations and Molecular Dynamics (MD), structural and energetic insights into Cl^–^ binding affinity and transmembrane transport by compounds **1–10** were obtained. The electrostatic potential on the molecular surface (*V*_S_) of the binding conformations of transporters **1–10** was computed from the optimised geometries of their Cl^–^ complexes (ESI Section S3.3†). Table 1 lists values of the electrostatic potential maximum (*V*_S,max_), which are intimately related to anion recognition by anionophores.8*a* ESI Fig. S4.23† shows their location, the distribution of the electrostatic potential and the optimised structures of the Cl^–^ complexes for compounds **2**, **6** and **9**. As these transporters have three thiourea binding units synergistically recognising Cl^–^, the *V*_S,max_ is located within the binding pocket.

Table 1 demonstrates that non-fluorinated compounds **1–4** exhibit similar *V*_S,max_ values, while their fluorinated analogues **5–8** have higher *V*_S,max_ values. Molecules **9** and **10**, with three mono-CF_3_ substituted chains, have *V*_S,max_ values slightly lower than **5–8**, but higher than **1–4**. This comparison shows that within each series of molecules (**1–4**, **5–8**, and **9–10**), the tripodal receptors have similar binding affinities for Cl^–^, which increase in the order **1–4** < **9–10** < **5–8**. However, there is no apparent relationship between the *V*_S,max_ values and the experimental binding constants (Table 1). For instance, compounds **5** and **7**, with similar *V*_S,max_ values, have the second lowest and highest binding constants of the series. These puzzling results indicate that other factors (*e.g.* solvent effects and/or conformational changes) play an important role in anion recognition in DMSO. Interestingly, compound **6**, which possesses the most acidic thiourea binding units, is the best transmembrane Cl^–^ transporter. However, the *V*_S,max_ and transport data are not easily related, prompting us to undertake a comprehensive MD investigation with POPC membrane models (ESI Sections S3.3 and S4.4†).

To evaluate the proposed anion carrier mechanism (step 2, Fig. 6), the passive diffusion of the tripodal Cl^–^ complexes in the POPC bilayer was studied with the complexes initially positioned in the bilayer core (scenario A) and simulated for 300 ns (single MD run). In addition, the ability of the tripodal molecules to permeate the water/lipid interface was investigated by positioning their Cl^–^ complexes in the water phase (scenario B) using 200 ns simulations (two independent MD runs). The position and orientation.

The position and orientation of the molecules in the bilayer was evaluated using the distances between the tripodal nitrogen (N_tren_) atom or the centre of mass defined by the terminal carbon atoms of the three thiourea chains (C_ter_) and the closest interface (P_int_, defined by the centre of mass of the 64 phosphorus atoms in that monolayer, see ESI Fig. S4.24–S4.26† for details).

**Fig. 8:**
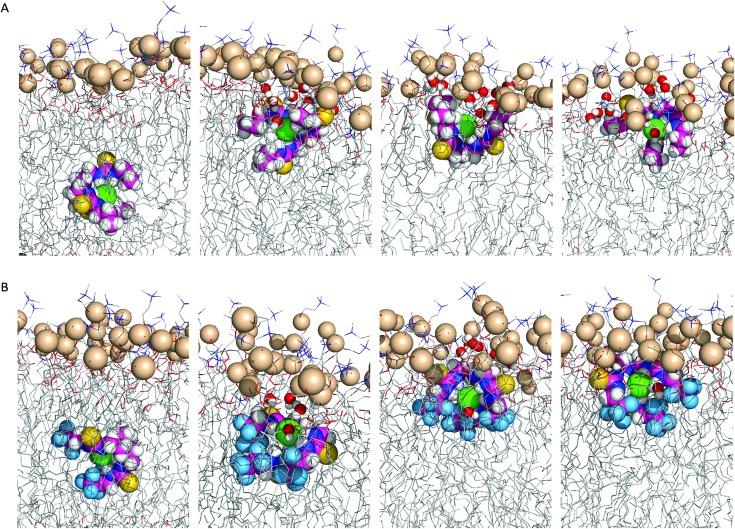
Consecutive snapshots depicting the diffusion of the Cl^–^ complex of **2** (A) and **6** (B) throughout the MD simulation in scenario A. The anionophore, complexed Cl^–^ and phosphorus atoms are represented by spheres. The remaining atoms of the bilayer are shown as lines. Hydrogen atoms are shown in white, oxygen atoms in red, sulfur atoms in yellow, nitrogen atoms in blue, fluorine atoms in light blue, phosphorus atoms in wheat, chloride in green.

In scenario A, the anionic tripodal complexes diffuse towards the water/lipid interface, with Cl^–^ sheltered from water molecules by the tripodal conformation of anionophores. Throughout the MD simulations of **1**, **4**, **7**, and **8** the anion is released to the water phase and the transporters form hydrogen bonding interactions with water molecules or phosphate head groups (ESI Fig. S4.24†). With increasing fluorination and thiourea chain length, the molecules adopt well-defined orientations within the highly-packed phospholipid medium, as observed for **3**, **4**, **6–10**, where the tren moiety is closer to the water phase and the chains are nearly aligned with the phospholipid tails. By contrast, **1**, **2** and **5**, with their shorter alkyl chains and reduced fluorination, adopt random orientations. Fig. 8 demonstrates these features for **2** and **6**.

In scenario B, all the compounds permeate the membrane, as free transporters or associated with Cl^–^ (ESI Fig. S4.25 and S4.26†). Most molecules release and take up Cl^–^ from the water phase before permeating the interface (ESI Fig. S4.25, B2.1, B1.5 and S4.26, B2.9†). The spatial dispositions attained below the interface are equivalent to the ones observed when the complexes are initially positioned in the bilayer core (scenario A).

**Fig. 9:**
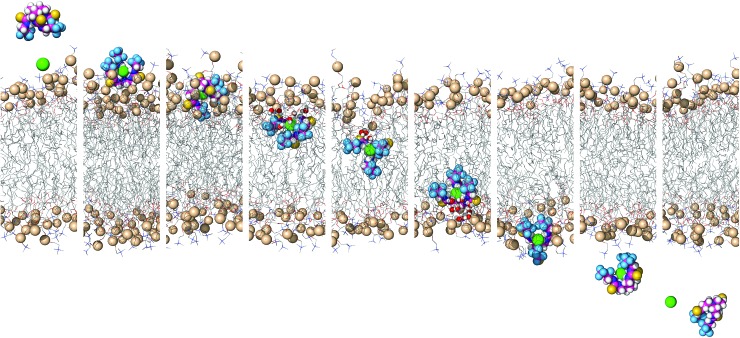
Consecutive snapshots depicting the movement of **6** across the POPC bilayer in a 170 ns long SMD simulation. The free transporter binds Cl^–^ before permeating and crossing the bilayer to the opposite side, where the anion dissociates from the transporter. For further caption details, see Fig. 8.

To simulate transmembrane Cl^–^ transport by **6**, the free receptor was dragged at a constant slow velocity (0.5 ns Å^–1^) from one aqueous phase to the other across the POPC bilayer (ESI Sections S3.3 and S4.4†). Fig. 9 and Movie S1† demonstrate that **6** can promote transmembrane Cl^–^ transport as an anion carrier, illustrating step 2 of the transport mechanism (Fig. 6). In addition, the slow translocation diffusion velocity allowed the tilting and tumbling of **6**·Cl^–^ within the highly packed POPC bilayer, adopting the same preferential orientation near both water/lipid interfaces. The slow permeation of **6**·Cl^–^ is accompanied by depression of the entry leaflet and many solvating water molecules (ESI Fig. S4.27†).

The free energy profiles associated with the transmembrane translocation of **2**, **6**, **9** and **8** as free transporters and Cl^–^ complexes were determined from Umbrella Sampling (US) MD simulations by computing the Potential of Mean Force (PMF) using the variational free energy profile (VFEP) method.^27^ The starting MD frames for the US windows of the free tripodal transporters were generated by Steered Molecular Dynamics (SMD) simulations where transporters were pulled from the bilayer core to the water phase, while the frames for the charged Cl^–^ complexes were generated from the passive diffusion MD simulations in both scenarios (ESI Section 3.3†). This approach reduced structural artefacts associated with the diffusion of polar entities across phospholipid bilayers.^28^

**Fig. 10:**
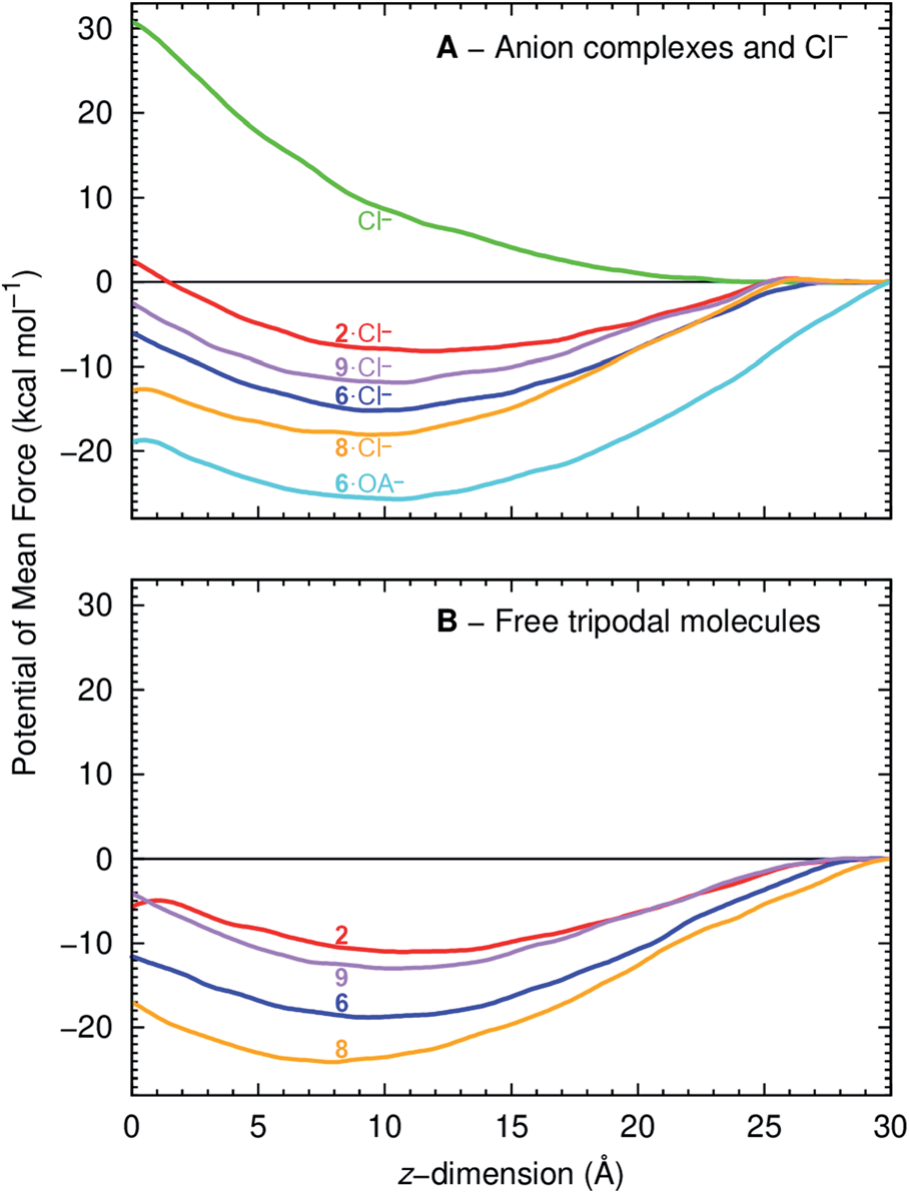
PMF as a function of anionophore distance to the membrane centre of mass (*z* = 0 Å), for the Cl^–^ complexes (top) and free transporters (bottom). The red, blue, purple and orange lines correspond to **2**, **6**, **9** and **8**. The green line corresponds to the PMF of a free Cl^–^ and the cyan line to the PMF of **6**·OA^–^.

The PMF of a Cl^–^ complex is intrinsically linked with step 2 of the transport mechanism, while the energy profile of a free transporter characterises step 7 (Fig. 6). Moreover, the PMF calculations for compounds **2**, **6**, and **9** energetically assess how fluorination affects anionophore-mediated Cl^–^ transport, while the effect of increasing chain length was evaluated with molecules **6** and **8**. The PMF profiles for Cl^–^ alone and complexed with **2**, **6**, **8** and **9** show that unassisted Cl^–^ translocation is highly disfavoured (Fig. 10A). Thus, an anionophore is essential to shuttle Cl^–^ across the bilayer. The PMF minima data further demonstrate that each Cl^–^ complex readily partitions from the water phase to the phospholipid bilayer, but faces an energy barrier (kcal mol^–1^) of 10.6 (**2**), 9.1 (**6**), 5.2 (**8**) and 9.3 (**9**) to cross the bilayer core towards the opposite leaflet (Fig. 10A). Interestingly, the energy profiles of the free transporters follow the same trend of the corresponding Cl^–^ complexes (Fig. 10). As a result, the PMF minima of the free transporters and of their complexes are linearly correlated (*R*^2^ = 0.94; ESI Fig. S4.31†).

The PMF profiles indicate that the free transporters surmount higher energy barriers (11.0, 18.8. 24.1 and 13.0 kcal mol^–1^ for **2**, **6**, **8** and **9**, respectively) than their corresponding complexes (8.2, 15.2, 18.1, 11.9 kcal mol^–1^ in the same order) to reach the water phase, revealing that the translocation of a complex (step 2, Fig. 6) is favoured over the translocation of its free transporter (step 7, Fig. 6B). They also suggest the existence of stronger anionophore–lipid interactions for fluorinated compounds (**6**, **8** and **9**) compared with non-fluorinated **2**.

**Fig. 11:**
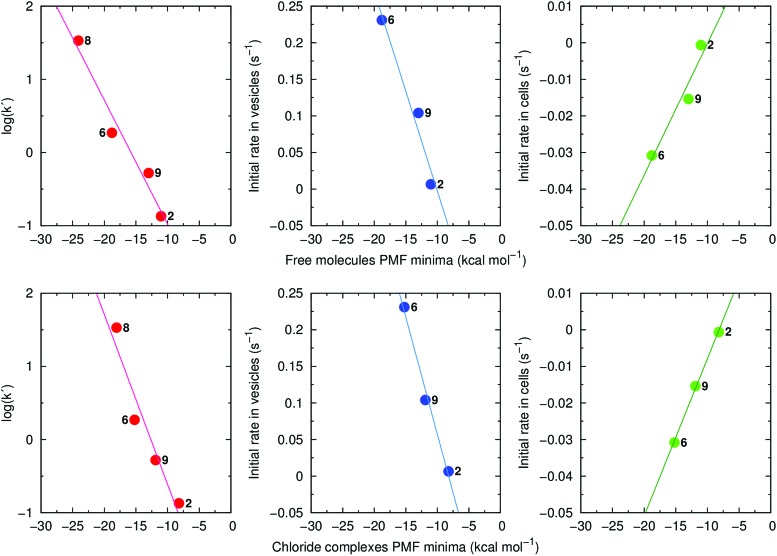
Left: log(*k*′) as a function of the PMF minima calculated for the free transporters **2**, **6**, **9** and **8** (top, *R*^2^ = 0.96) and their Cl^–^ complexes (bottom, *R*^2^ = 0.93). Middle: initial rate of Cl^–^ efflux from vesicles (values are from the Gr-assays for non-fluorinated **2** and the OA-assays for fluorinated **6** and **9**) as a function of the PMF minima calculated for the free transporters **2**, **6**, and **9** (top, *R*^2^ = 0.96) and their Cl^–^ complexes (bottom, *R*^2^ = 0.99). Right: initial rate of Cl^–^ efflux in cells as a function of the PMF minima calculated for the free transporters **2**, **6**, and **9** (top, *R*^2^ = 0.93) and their Cl^–^ complexes (bottom, *R*^2^ = 1.00).

Of note, retention times (log(*k*′)) are strongly correlated with the global minima of the individual PMF profiles for the free transporters (*R*^2^ = 0.96) and their Cl^–^ complexes (*R*^2^ = 0.93) (Fig. 11, left). Strong linear relationships were also found when the PMF minima were plotted against the initial rates of Cl^–^ efflux from vesicles (Fig. 11, centre) or from cells (Fig. 11, right). These results demonstrate that lipophilicity, determined by degree of fluorination and thiourea chain length, plays an important role in the interaction of **2**, **9**, **6** and **8** with phospholipids and hence, their transport activity. Using Molecular Mechanics (MM), the interaction energies between transporters and POPC lipids were calculated at the positions determined by each PMF profile global minimum, where the compounds adopt a well-defined orientation within the highly-packed lipid medium (ESI Section 3.3.3†). The interaction energies are composed of electrostatic and van der Waals contributions with the latter being the main contributor to the total MM interaction energy (Table S4.3†). This reveals that transport activity is strongly dependent on interactions between anionophores and phospholipids. Consistent with this idea, for free transporters there is a direct relationship between van der Waals contributions and the global minima in the individual free energy profiles (*R*^2^ = 0.90; Fig. S4.32†).

Because OA markedly enhanced anion transport by fluorinated transporters (*e.g.***6**) (Fig. 5), the interaction of **6**·OA^–^ with a POPC bilayer was investigated by US MD simulations with the reconstruction of the corresponding PMF (ESI Section 4.4.1†). The orientation of the long **6**·OA^–^ alkyl chain relatively to the membrane was assessed by monitoring the position of the terminal CH_3_ carbon atom (OA_CH_3__) and the position of the tripodal nitrogen atom (N_tren_). ESI Fig. S4.33† demonstrates that in the middle of the POPC bilayer (*z* = 0 Å) where lipid chains are highly disordered, the OA^–^ chain is randomly orientated. However, as the complex moves towards the water/lipid interface the OA chain becomes aligned with the phospholipid alkyl tails.

Fig. 10A and ESI Fig. S4.30 and S4.34† plot the PMF reconstructed from simulations with OA^–^. The data suggest that the recycling of the transporter after Cl^–^ release is energetically favoured when associated with OA^–^, due to interactions between the long alkyl chain of OA^–^ and phospholipid tails. Thus, OA^–^ facilitates the back diffusion of this molecule (Fig. 6C, step 12) after it has promoted Cl^–^ transport (Fig. 6, steps 2 and 3). However, the high energy barrier for **6**·OA^–^ to reach the interface suggests that this event is unlikely. Consequently, dissociation of the **6**·OA^–^ complex should occur just under the water/lipid interface, releasing the transporter to bind a solvated Cl^–^ and reinitiate anion transport (Fig. 6C, step 1).

## Conclusions

This study explored the relationship between fluorination of alkyl substituents in tripodal anion transporters and their ability to transport anions across lipid bilayers and cell membranes. Fluorination increased strongly the activity of compounds with shorter alkyl tails through enhanced lipophilicity. Importantly, fluorinated anionophores displayed an unusual transport mechanism because the free transporter failed to rapidly cross the membrane in vesicle-based pH discharge assays. Consistent with these data, molecular modelling revealed stronger interactions of fluorinated anionophores with phospholipid bilayers than non-fluorinated analogues. It also demonstrated that transport activity relies on a finely-tuned balance between lipophilicity and anion binding affinity for each transporter. However, Cl^–^ uptake and release occurs at the water/lipid interface without transporters partitioning into the water phase, as recently found for squaramide transporters.^29^ Thus, these anionophores assist Cl^–^ translocation by a shuttling mechanism.

In conclusion, this work demonstrates that full characterisation of transport mechanisms in proton transporter-assisted vesicle assays is an important tool to improve the activity of synthetic transporters. However, care must be taken to ensure that the rate limiting step in vesicle assays corresponds to the same step in cell-based assays given the complexity of biological membranes. Using this approach, compounds with therapeutic potential will be identified.

## Conflicts of interest

There are no conflicts to declare.

## Supplementary Material

Supplementary information

Crystal structure data
