# Supplementary material for: Fluorinated synthetic anion carriers: experimental and computational insights into transmembrane chloride transport
Source: Chem Sci. 2018 Dec 14;10(7):1976–85. doi: 10.1039/c8sc05155k (PMC6381411; doi:10.1039/c8sc05155k)
Supplement: Supplementary file 1 [file SC-010-C8SC05155K-s001.pdf]

Electronic Supplementary Material (ESI) for Chemical Science.
This journal is © The Royal Society of Chemistry 2018

Fluorinated synthetic anion carriers: experimental and computational insights into transmembrane chloride transport

M. J. Spooner, H. Li, I. Marques, P. M. R. Costa, X. Wu, E. N. W. Howe, N. Busschaert, S. J. Moore, M.E. Light, D. N. Sheppard, V. Félix, & P. A. Gale

Electronic Supplementary Information

S1 Compound Overview 2

S2 Synthesis 3

S2.1 Synthetic Procedures 3

S2.2 NMR Spectra 7

S3 Experimental Methods 15

S3.1 Transport Studies 15

S3.2 NMR Titrations 16

S3.3 Computational Methods 16

S3.4 Anionophore-Mediated Anion Transport in Cells 20

S4 Supplementary Data 22

S4.1 Lipophilicity Estimation 22

S4.2 NMDG Assay Transport Curves 23

S4.3 NMR Titration Fit Plots 29

S4.4 Additional Modelling Data 34

S4.5 Anion Transport in Cells 45

S4.6 Crystal Data 46

S5 References 48

S1 Compound Overview

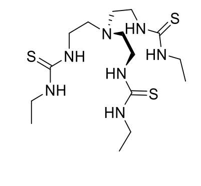

1

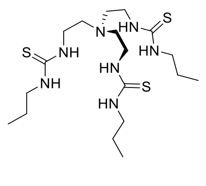

2

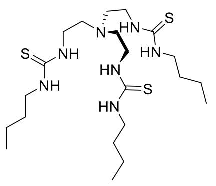

3

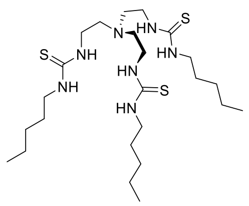

4

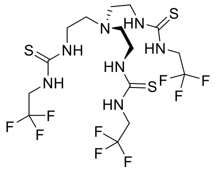

5

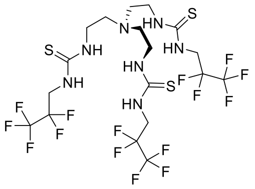

6

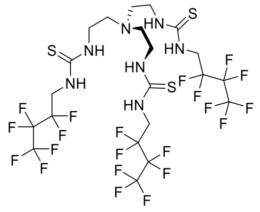

7

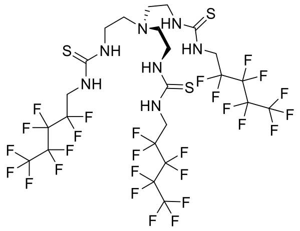

8

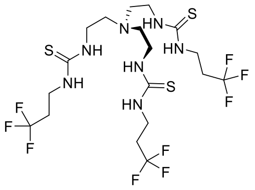

9

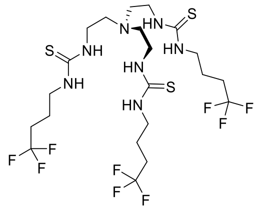

10

Figure S1.1 Structure of the fluorinated tren-based compounds.

S2 **Synthesis**

S2.1 **Synthetic Procedures**

NMR spectra were recorded on Bruker AVII400 or Bruker AVIIIHD400 FT-NMR spectrometers in the indicated solvent at 298 K. Chemical shifts for proton and carbon spectra are reported on the delta scale in ppm and were referenced to residual solvent references or internal TMS reference. High resolution mass spectra were recorded using positive/negative ion electrospray ionisation and analysed using a MaXis (Bruker Daltonics, Bremen, Germany) mass spectrometer equipped with a Time of Flight (TOF) analyser. Samples were introduced to the mass spectrometer via a Dionex Ultimate 3000 autosampler and uHPLC pump. Mobile phase: gradient 20% acetonitrile (0.2% formic acid) to 100% acetonitrile (0.2% formic acid) in five minutes at 0.6 mL min. Column: Acquity UPLC BEH C18 (Waters) 1.7 micron 50 x 2.1 mm. Starting materials were used as provided by suppliers. Dichloromethane was distilled over sodium hydroxide under nitrogen before use. Other solvents were used without further purification. The synthesis of compound **4** has been previously reported.[1]

A

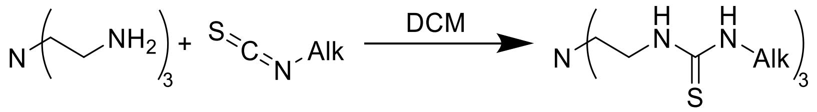

B

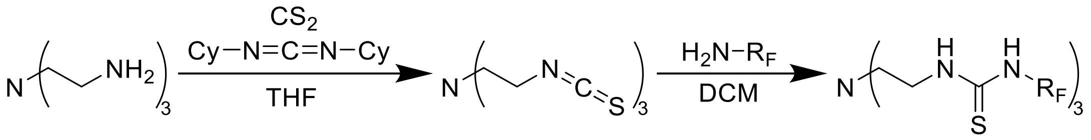

11

Scheme 2.1 General synthesis of the fluorinated tren-based compounds, **A**: compounds **1** - **4**, **B**: compounds **5** - **10**. Reaction conditions and work ups varied, full experimental details follow.

S2.1.1 Tris(2-isothiocyanatoethyl)amine **11**

This compound was synthesised by an adapted literature procedure.[2]

Tris-(2-aminoethyl)amine (10 mmol) was dissolved in THF (150 ml) under N_2 atmosphere. Carbon disulfide (200 mmol) was added yielding a cloudy solution. *N,N*-dicyclohexylcarbodiimide (31 mmol) was added and the reaction stirred overnight.

The yellow suspension was filtered 3 times to remove white/yellow solid precipitate. The solvent was removed from the filtrate under reduced pressure and the residue purified by flash chromatography (eluent: DCM) yielding pale yellow solid.

Yield: 32 %.

S2.1.2 1,1',1''-(Nitrilotris(ethane-2,1-diyl))tris(3-ethylthiourea) **1**

Tris(2-aminoethyl)amine (1 mmol) was dissolved in dichloromethane (10 ml). Ethyl isothiocyanate (3 mmol) was added and the solution stirred at room temperature overnight. The solvent was removed under reduced pressure and the residue recrystallised from ethyl acetate/hexane yielding white crystals.

Yield: 32 %. HRMS ESI$^+$ = $[C_{15}H_{34}N_7S_3]^+$: 408.2032 (calc), 408.22040 (found), -2.0 ppm (err). ^{1}H NMR (400 MHz, DMSO-d_6) δ ppm 1.05 (t, *J*=7.21 Hz, 3 H) 2.52 - 2.70 (m, 2 H) 3.39 - 3.55 (m, 2 H) 7.19 (br s, 1 H) 7.49 (br s, 1 H). ^{13}C NMR (101 MHz, DMSO-d_6) δ ppm 14.9, 38.7, 42.0, 53.1, 182.0. Melting point: 122.4 – 123.6 °C.

S2.1.3 1,1',1''-(Nitrilotris(ethane-2,1-diyl))tris(3-propylthiourea) **2**

Tris(2-aminoethyl)amine (1 mmol) was dissolved in dichloromethane (10 ml). Propyl isothiocyanate (3 mmol) was added and the solution stirred at room temperature overnight. The solvent was removed under reduced pressure yielding a yellow oil.

Oil was purified by flash chromatography (eluent: ethyl acetate) yielding a colourless oil. Appropriate phases were combined and solvent removed under reduced pressure. The residue was dried under vacuum yielding an amorphous white solid.

Yield: 62 %. HRMS ESI$^+$ = $[C_{18}H_{40}N_7S_3]^+$: 450.2502 (calc), 450.2498 (found), 0.8 ppm (err). ^{1}H NMR (400 MHz, DMSO-d_6) δ ppm 0.85 (t, *J*=7.40 Hz, 3 H) 1.47 (sxt, *J*=7.29 Hz, 2 H) 2.52 - 2.70 (m, 2 H) 3.08 - 3.31 (m, 2 H) 3.34 - 3.57 (m, 2 H) 7.20 (br s, 1 H) 7.51 (br s, 1 H). ^{13}C NMR (101 MHz, DMSO-d_6) δ ppm 11.8, 22.5, 42.0, 45.8, 53.1, 182.4. Melting point: 109.7 – 110.3 °C.

S2.1.4 1,1',1''-(Nitrilotris(ethane-2,1-diyl))tris(3-butylthiourea) **3**

This compound had been previously reported[3].

Tris(2-aminoethyl)amine (1 mmol) was dissolved in dichloromethane (10 ml). Butyl isothiocyanate (3 mmol) was added and the solution stirred at room temperature overnight. The solvent was removed under reduced pressure yielding a yellow oil.

Oil was purified by flash chromatography (eluent: ethyl acetate) yielding a colourless oil. Appropriate phases were combined and solvent removed under reduced pressure. The residue was dried under vacuum yielding an amorphous white solid.

Yield: 64 %. HRMS ESI$^+$ = $[C_{21}H_{46}N_7S_3]^+$: 492.2971 (calc), 492.2978 (found), -1.4 ppm (err). ^{1}H NMR (400 MHz, DMSO-d_6) δ ppm 0.87 (t, *J*=7.34 Hz, 3 H) 1.28 (dq, *J*=14.93, 7.29 Hz, 2 H) 1.44 (quin, *J*=7.27 Hz, 2

H) 2.52 - 2.70 (m, 2 H) 3.38 - 3.57 (m, 2 H) 7.19 (br s, 1 H) 7.48 (br s, 1 H). ^{13}C NMR (101 MHz, DMSO-*d*$_6$) δ ppm 14.2, 20.1, 31.3, 42.0, 43.7, 53.1, 182.4. Melting point 83.5 – 85.1 °C.

S2.1.5 1,1',1''-(Nitrilotris(ethane-2,1-diyl))tris(3-(2,2,2-trifluoroethyl)thiourea) **5**

Tris(2-isothiocyanatoethyl)amine **11** (1 mmol) was dissolved in dichloromethane (10 ml). 2,2,2-Trifluoroethylamine (3 mmol) was added and the solution stirred at room temperature for 24 hours. The solvent was removed under reduced pressure and the residue was recrystallised from dichloromethane /hexane yielding a white powder.

Yield: 66 %. HRMS ESI$^+$ = [$C_{15}H_{25}F_9N_7S_3$]$^+$: 570.1184 (calc), 570.1170 (found), 2.5 ppm (err). ^{1}H NMR (400 MHz, DMSO-*d*$_6$) δ ppm 2.65 (br t, *J*=6.85 Hz, 3 H) 3.53 (br s, 2 H) 4.29 - 4.47 (m, 3 H) 7.66 (br s, 1 H) 7.97 (br s, 1 H). ^{13}C NMR (101 MHz, DMSO-*d*$_6$) δ ppm 42.6, 44.6, 52.6, 125.3, 184.4. ^{19}F NMR (376 MHz, DMSO-*d*$_6$) δ ppm -70.6143 (br s, 1 F). Melting point: 142.0 – 142.8 °C.

S2.1.6 1,1',1''-(nitrilotris(ethane-2,1-diyl))tris(3-(2,2,3,3,3-pentafluoropropyl)thiourea) **6**

Tris(2-isothiocyanatoethyl)amine **11** (1 mmol) was dissolved in dichloromethane (10 ml). 2,2,3,3,3-Pentafluoropropylamine (3 mmol) was added and the solution stirred at room temperature for 24 hours.

The solvent was removed under reduced pressure and the residue purified by flash chromatography on an Isolute Si-II column. The residue was loaded with diethyl ether and eluted with ethyl acetate. The ethyl acetate phase was evaporated yielding a colourless oil. The oil was further purified by recrystallisation from dichloromethane/hexane yielding a white solid.

Yield: 18 %. HRMS ESI$^+$ = [$C_{18}H_{25}F_{15}N_7S_3$]$^+$: 720.1089 (calc), 720.1094 (found), -0.7 ppm (err). ^{1}H NMR (400 MHz, DMSO-*d*$_6$) δ ppm 2.66 (br t, *J*=6.85 Hz, 2 H) 3.54 (br s, 2 H) 4.50 (td, *J*=16.11, 5.69 Hz, 2 H) 7.70 (br s, 1 H) 7.81 - 8.10 (m, 1 H). ^{13}C NMR (101 MHz, DMSO-*d*$_6$) δ ppm 42.7, 52.6, 113.9, 117.40, 184.7. ^{19}F NMR (376 MHz, DMSO-*d*$_6$) δ ppm -120.4409 (br t, *J*=15.61 Hz, 1 F) -83.5787 (s, 1 F). Melting point: 130.9 – 131.5 °C.

S2.1.7 1,1',1''-(Nitrilotris(ethane-2,1-diyl))tris(3-(2,2,3,3,4,4,4- heptafluorobutyl)thiourea) **7**

A suspension of 1H,1H-perfluorobutylamine (0.49 g, 2.5 mmol) and tris(2- isothiocyanatoethyl)amine (**11**, I.1.16) (0.22 g, 0.81 mmol) in DCM (8 ml) was left to stand for 6 days without stirring. The white crystals formed were collected and washed with DCM to give pure product.

Yield: 58 %. ^{1}H NMR (400 MHz, DMSO-*d*$_6$) δ = 7.91 (br s, 1H), 7.73 (br s, 1H), 4.54 (dt, *J*=5.7, 16.9 Hz, 2H), 3.33 (s, 1H), 2.67 (br t, *J*=6.8 Hz, 2H). ^{13}C NMR (101MHz, DMSO-*d*$_6$) δ = 184.7, 117.8 (qt, *J*=286.8, 33.8 Hz), 115.8 (tt, *J*=254.9, 30.5 Hz), 109.0 (tq, *J*=264.1, 36.7 Hz), 52.6, 43.0 (t, *J*=21.3 Hz), 42.7. 19F

NMR (376MHz, DMSO-d_6) δ = -80.43 (br t, *J*=8.7 Hz), -117.35 - -118.13 (m), -127.67 (br s). Melting point 152.5 – 153.2°C.

S2.1.8 1,1',1''-(Nitrilotris(ethane-2,1-diyl))tris(3-(2,2,3,3,4,4,5,5,5-nonafluoropentyl)thiourea) **8**

A solution of 1H,1H-perfluoropentylamine (0.54 g, 2.2 mmol) and tris(2-isothiocyanatoethyl)amine (**11**, I.1.16) (0.19 g, 0.72 mmol) in DCM (4 ml) was left to stand for 6 days without stirring. The white crystals formed were collected and washed with DCM to give pure product.

Yield: 72 %. $_1$H NMR (400 MHz, DMSO-d_6) δ = 7.91 (br s, 1H), 7.72 (br s, 1H), 4.56 (dt, *J*=5.6, 16.8 Hz, 2H), 3.55 (br s, 2H), 2.67 (br t, *J*=6.8 Hz, 2H). $_{19}$F NMR (376MHz, DMSO-d_6) δ = -80.67 (br t, *J*=8.7 Hz), -117.16 (br s), -124.34 (br s), -126.01 (br t, *J*=12.1 Hz). Melting point 164.4 – 165.2°C.

S2.1.9 1,1',1''-(nitrilotris(ethane-2,1-diyl))tris(3-(3,3,3-trifluoropropyl)thiourea) **9**

Tris(2-isothiocyanatoethyl)amine **11** (1 mmol) was dissolved in dichloromethane (10 ml). 3,3,3-Trifluoropropylamine (3 mmol) was added and the solution stirred at room temperature for 24 hours.

The solvent was removed under reduced pressure and the residue purified by flash chromatography on an Isolute Si-II column. The residue was loaded with diethyl ether and eluted with ethyl acetate. The ethyl acetate phase was evaporated yielding a colourless oil which formed an amorphous solid on drying.

Yield: 92 %. HRMS ESI$^+$ = $[C_{18}H_{31}F_9N_7S_3]^+$: 612.1654 (calc), 612.1668 (found), -2.3 ppm (err). ^{1}H NMR (400 MHz, DMSO-d_6) δ ppm 2.63 (t, *J*=6.60 Hz, 3 H) 3.35 - 3.53 (m, 2 H) 3.54 - 3.73 (m, 2 H) 7.47 (br s, 1 H) 7.55 - 7.73 (m, 1 H). ^{13}C NMR (101 MHz, DMSO-d_6) δ ppm 33.0, 37.3, 42.0, 52.9, 127.3, 183.0 ^{19}F NMR (376 MHz, DMSO-d_6) δ ppm -63.7752 (br t, *J*=12.14 Hz, 1 F). Melting point: 82.3 – 83.6 °C.

S2.1.10 1,1',1''-(nitrilotris(ethane-2,1-diyl))tris(3-(4,4,4-trifluorobutyl)thiourea) **10**

Tris(2-isothiocyanatoethyl)amine 11 (1 mmol) was dissolved in dichloromethane (10 ml). 4,4,4-Trifluorobutylamine (3 mmol) was added and the solution stirred at room temperature for 24 hours.

The solvent was removed under reduced pressure and the residue purified by flash chromatography on an Isolute Si-II column. The residue was loaded with diethyl ether and eluted with ethyl acetate. The ethyl acetate phase was evaporated yielding a colourless oil which formed an amorphous solid on drying.

Yield: 77 %. HRMS ESI$^+$ = $[C_{21}H_{37}F_9N_7S_3]^+$: 654.2123 (calc), 654.2126 (found), -0.4 ppm (err). ^{1}H NMR (400 MHz, DMSO-d_6) δ ppm 1.70 (tt, *J*=7.50 Hz, 2 H) 2.24 (qt, *J*=11.60, 8.30 Hz, 2 H) 2.62 (br t, *J*=6.72 Hz, 2 H) 3.34 - 3.57 (m, 4 H) 7.28 (br s, 1 H) 7.61 (br s, 1 H). ^{13}C NMR (101 MHz, DMSO-d_6) δ ppm 22.1,

30.7, 42.0, 42.6, 52.9,128.1, 182.7. ^{19}F NMR (376 MHz, DMSO-d_6) δ ppm -64.8359 (t, J=11.27 Hz, 1 F). Melting point: 97.6 – 99.9 °C.

S2.2 NMR Spectra

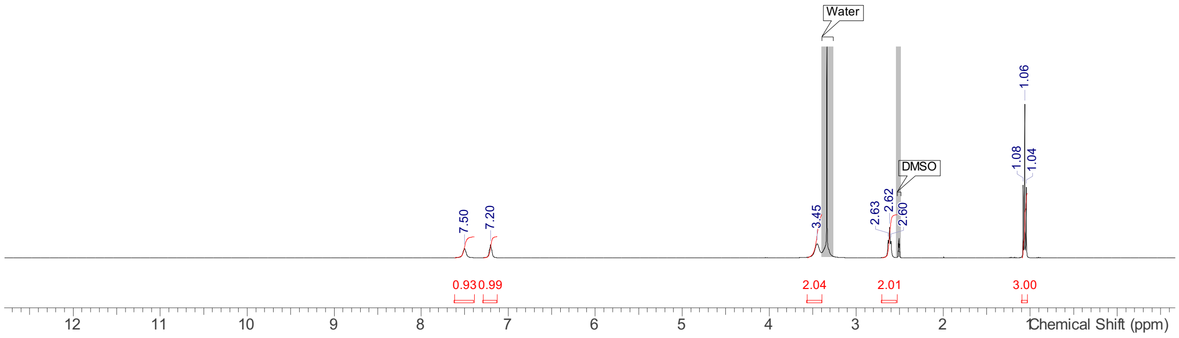

Figure S2.1 ^{1}H NMR (400 MHz, DMSO-d$_6$) spectrum of 1,1',1''-(nitrilotris(ethane-2,1-diyl))tris(3-ethylthiourea) **1**.

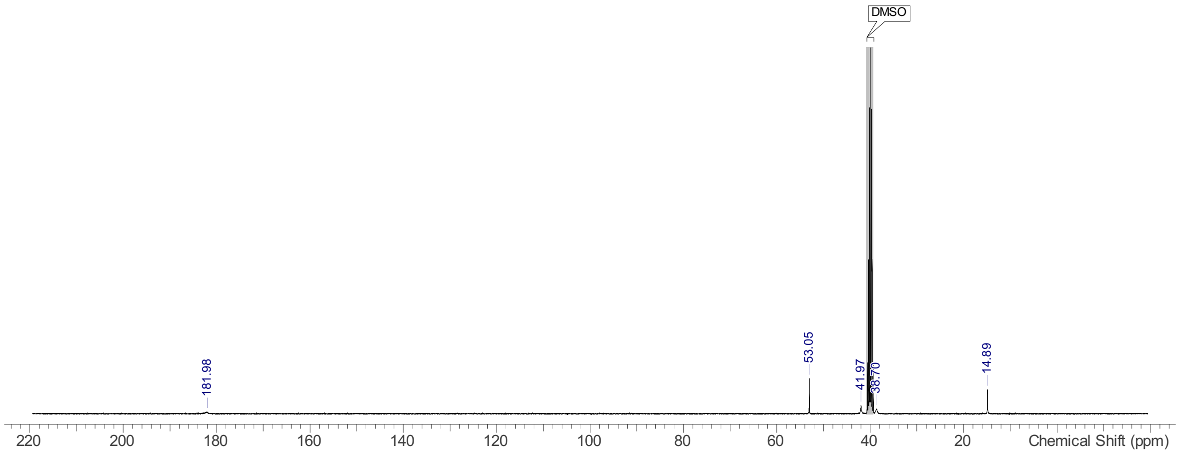

Figure S2.2 ^{13}C NMR (101 MHz, DMSO-d$_6$) spectrum of 1,1',1''-(nitrilotris(ethane-2,1-diyl))tris(3-ethylthiourea) **1**.

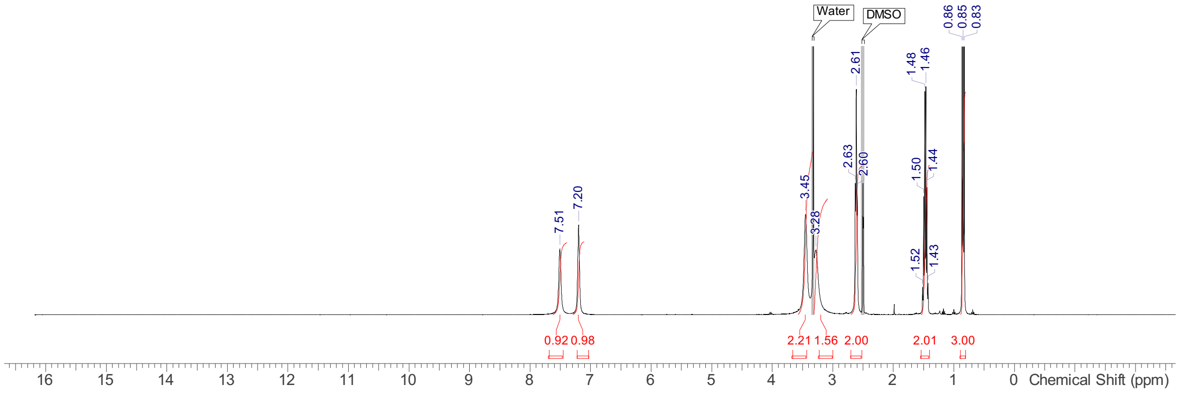

Figure S2.3 ^{1}H NMR (400 MHz, DMSO-d$_6$) spectrum of 1,1',1''-(nitrilotris(ethane-2,1-diyl))tris(3-propylthiourea) **2**.

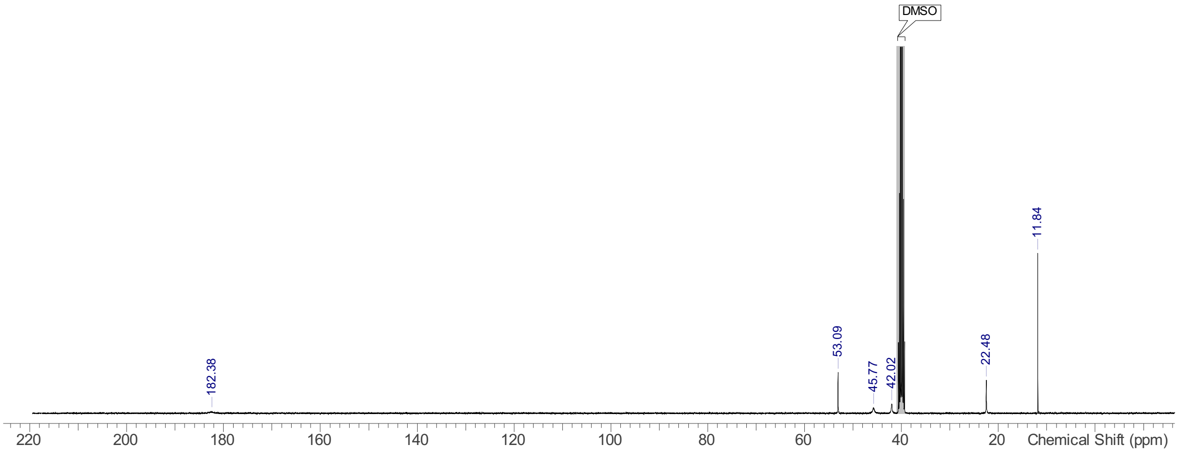

Figure S2.4 ^{13}C NMR (101 MHz, DMSO-d$_6$) spectrum of 1,1',1''-(nitrilotris(ethane-2,1-diyl))tris(3-propylthiourea) **2**.

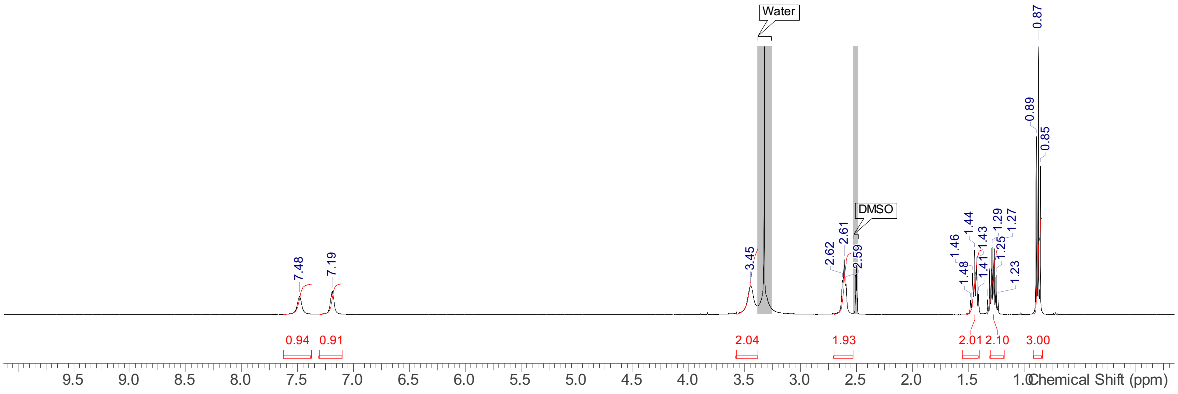

Figure S2.5 ^{1}H NMR (400 MHz, DMSO-d$_6$) spectrum of 1,1',1''-(nitrilotris(ethane-2,1-diyl))tris(3-butylthiourea) **3**.

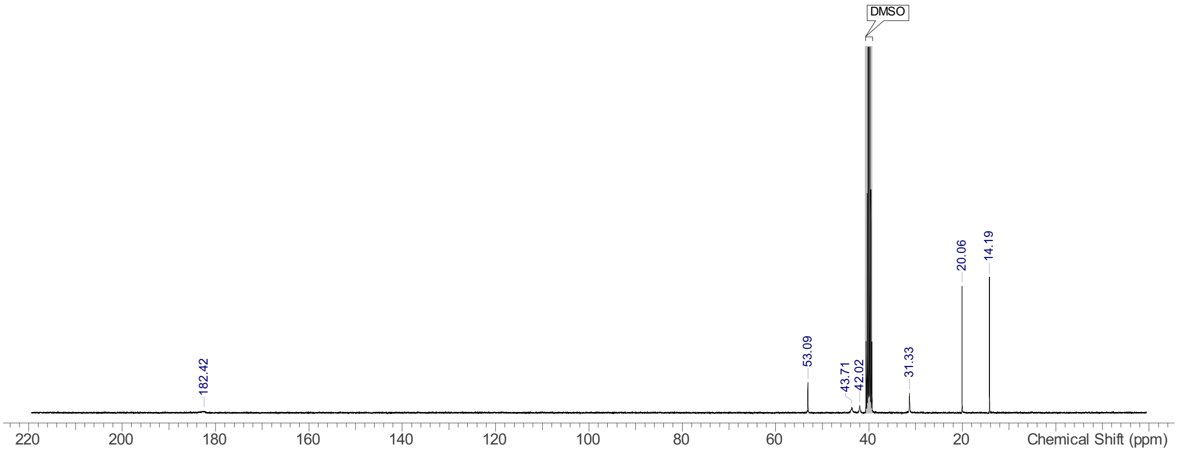

Figure S2.6 ^{13}C NMR (101 MHz, DMSO-d$_6$) spectrum of 1,1',1''-(nitrilotris(ethane-2,1-diyl))tris(3-butylthiourea) **3**.

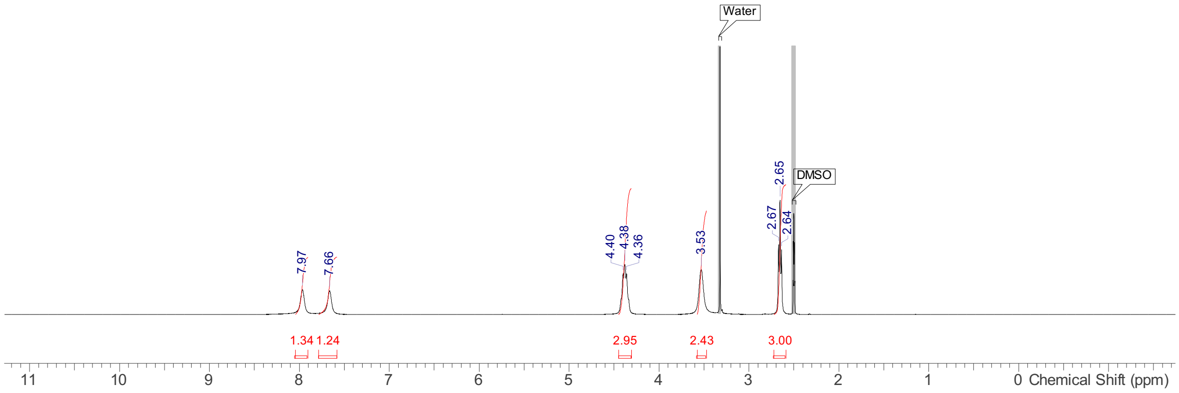

Figure S2.7 ^{1}H NMR (400 MHz, DMSO-d_6) spectrum of 1,1',1''-(nitrilotris(ethane-2,1-diyl))tris(3-(2,2,2-trifluoroethyl)thiourea) **5**.

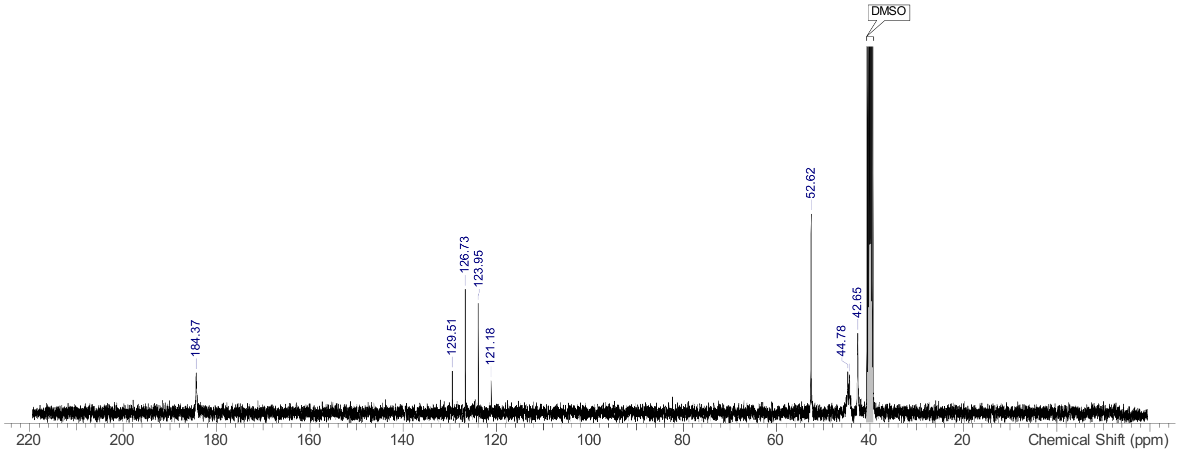

Figure S2.8 ^{13}C NMR (101 MHz, DMSO-d_6) spectrum of 1,1',1''-(nitrilotris(ethane-2,1-diyl))tris(3-(2,2,2-trifluoroethyl)thiourea) **5**.

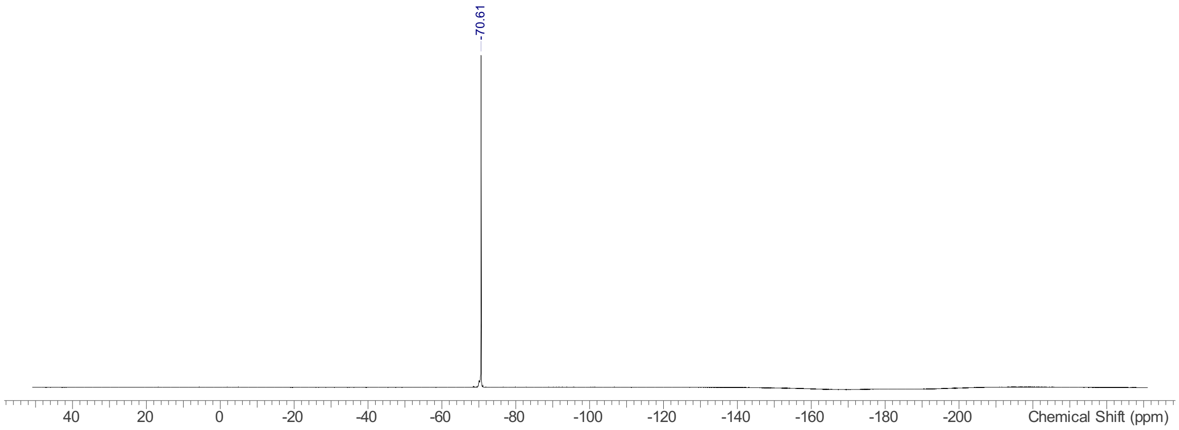

Figure S2.9 ^{19}F NMR (376 MHz, DMSO-d_6) spectrum of 1,1',1''-(nitrilotris(ethane-2,1-diyl))tris(3-(2,2,2-trifluoroethyl)thiourea) **5**.

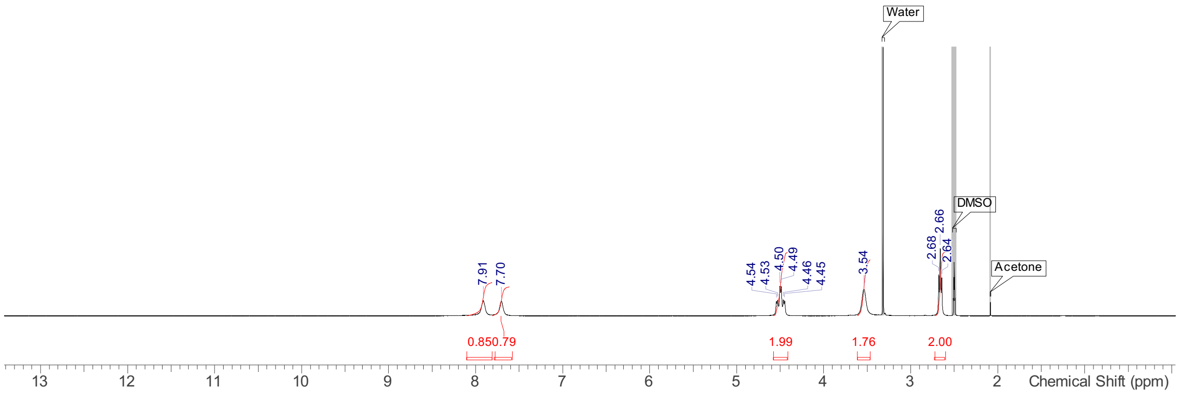

Figure S2.10 ^{1}H NMR (400 MHz, DMSO-d$_6$) spectrum of 1,1',1''-(nitrilotris(ethane-2,1-diyl))tris(3-(2,2,3,3,3-pentafluoropropyl)thiourea) **6**.

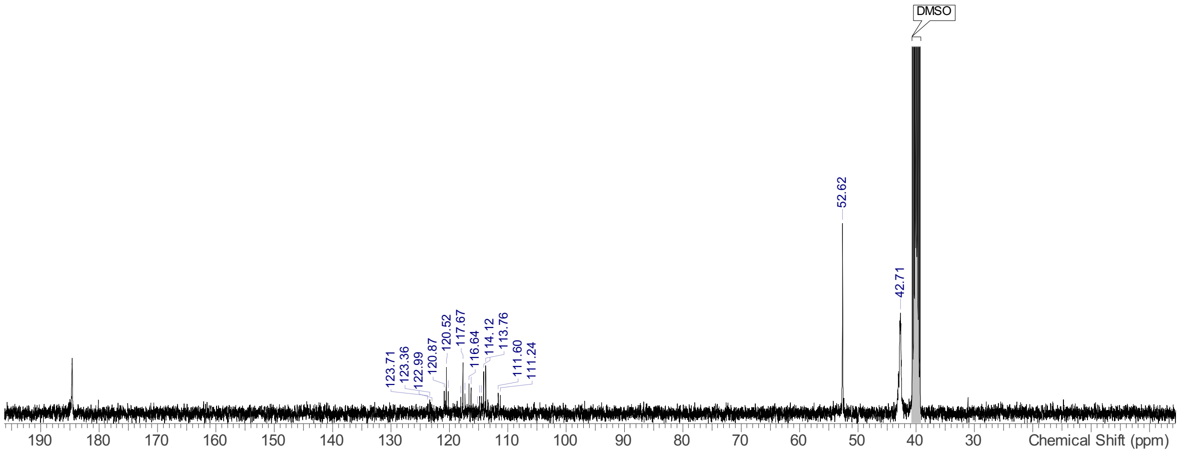

Figure S2.11 ^{13}C NMR (101 MHz, DMSO-d$_6$) spectrum of 1,1',1''-(nitrilotris(ethane-2,1-diyl))tris(3-(2,2,3,3,3-pentafluoropropyl)thiourea) **6**.

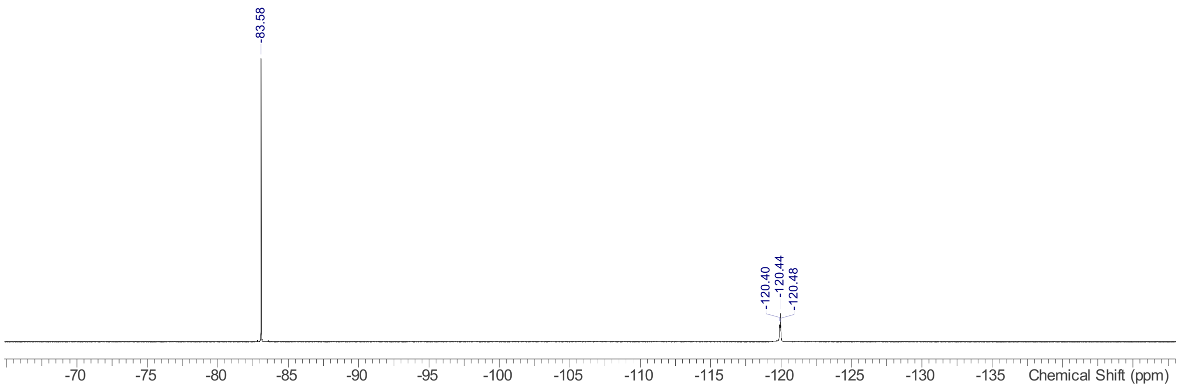

Figure S2.12 ^{19}F NMR (376 MHz, DMSO-d$_6$) spectrum of 1,1',1''-(nitrilotris(ethane-2,1-diyl))tris(3-(2,2,3,3,3-pentafluoropropyl)thiourea) **6**.

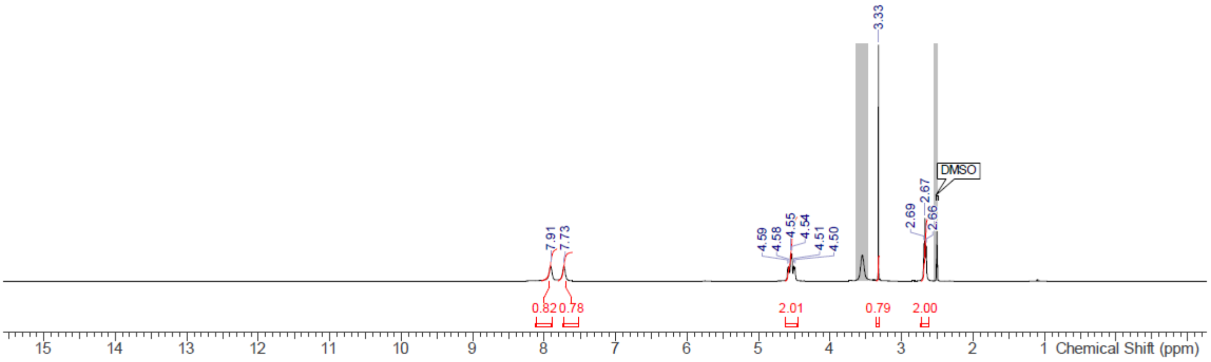

Figure S2.13 ¹H NMR (400 MHz, DMSO-d$_6$) spectrum of 1,1',1''-(Nitrilotris(ethane-2,1-diyl))tris(3-(2,2,3,3,4,4,4-heptafluorobutyl)thiourea) **7.**

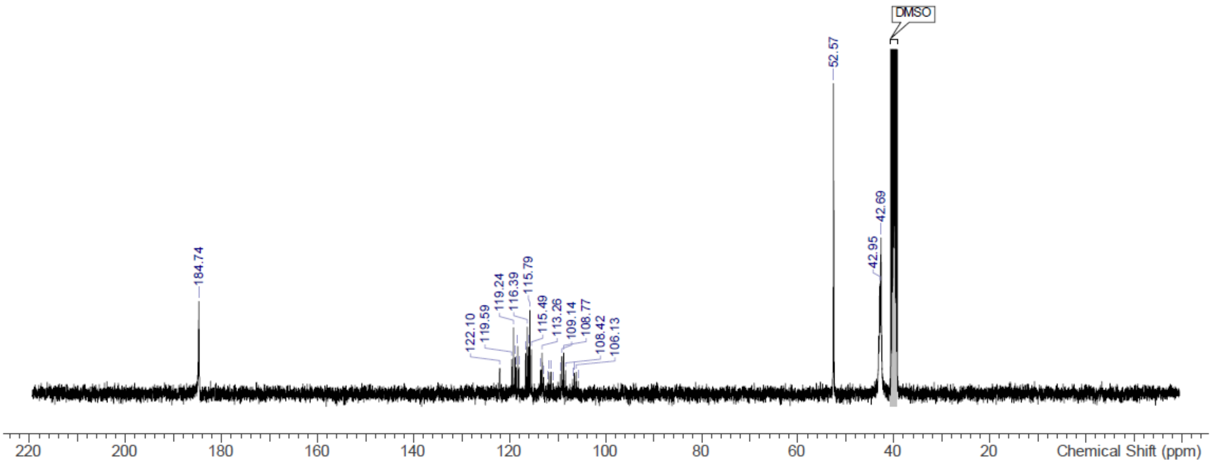

Figure S2.14 ¹³C NMR (101 MHz, DMSO-d$_6$) spectrum of 1,1',1''-(Nitrilotris(ethane-2,1-diyl))tris(3-(2,2,3,3,4,4,4-heptafluorobutyl)thiourea) **7**.

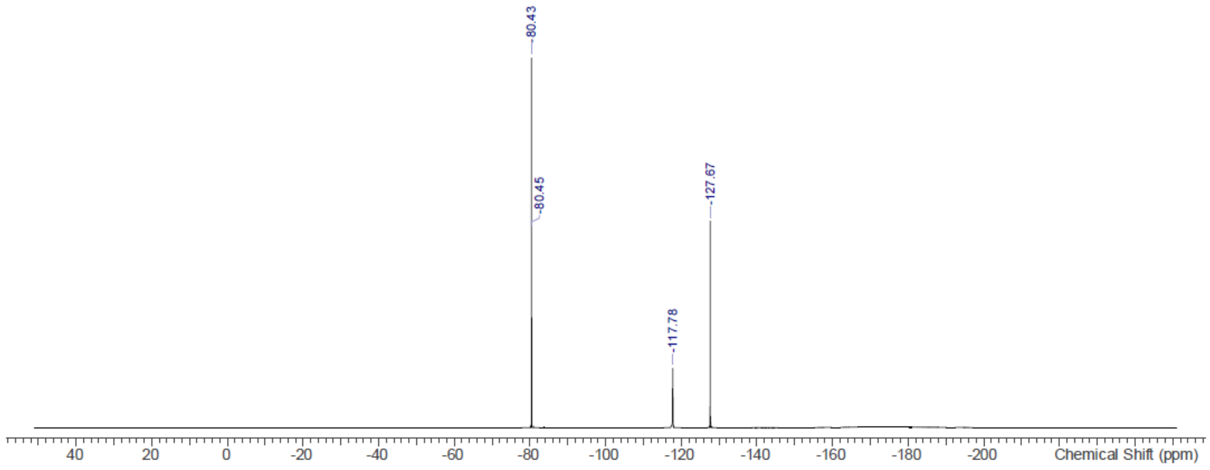

Figure S2.15 ¹⁹F NMR (376 MHz, DMSO-d$_6$) spectrum of 1,1',1''-(Nitrilotris(ethane-2,1-diyl))tris(3-(2,2,3,3,4,4,4-heptafluorobutyl)thiourea) **7**.

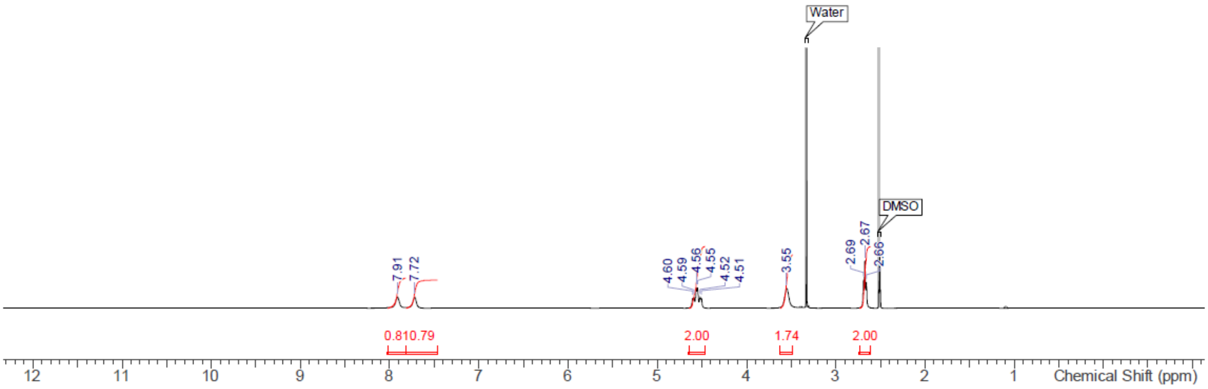

Figure S2.16 ¹H NMR (400 MHz, DMSO-d$_6$) spectrum of 1,1',1''-(Nitrilotris(ethane-2,1-diyl))tris(3-(2,2,3,3,4,4,5,5,5-nonafluoropentyl)thiourea) **8**.

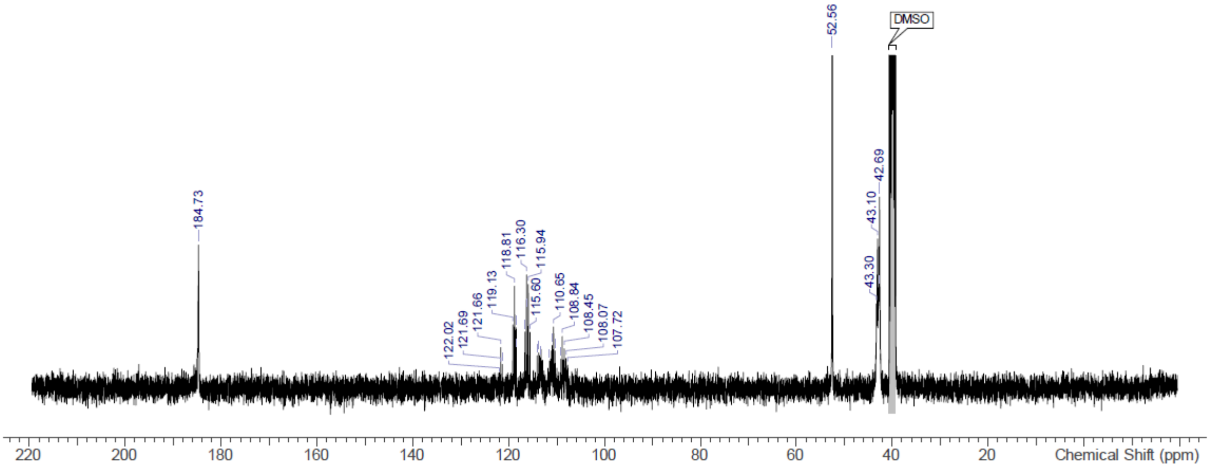

Figure S2.17 ¹³C NMR (101 MHz, DMSO-d$_6$) spectrum of 1,1',1''-(Nitrilotris(ethane-2,1-diyl))tris(3-(2,2,3,3,4,4,5,5,5-nonafluoropentyl)thiourea) **8**.

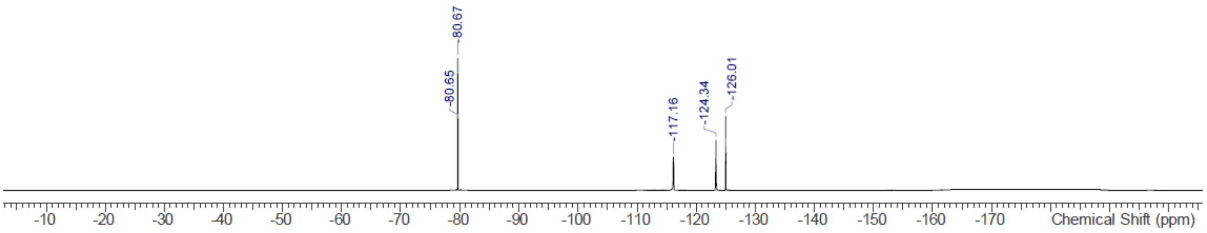

Figure S2.18 ¹⁹F NMR (376 MHz, DMSO-d$_6$) spectrum of 1,1',1''-(Nitrilotris(ethane-2,1-diyl))tris(3-(2,2,3,3,4,4,5,5,5-nonafluoropentyl)thiourea) **8**.

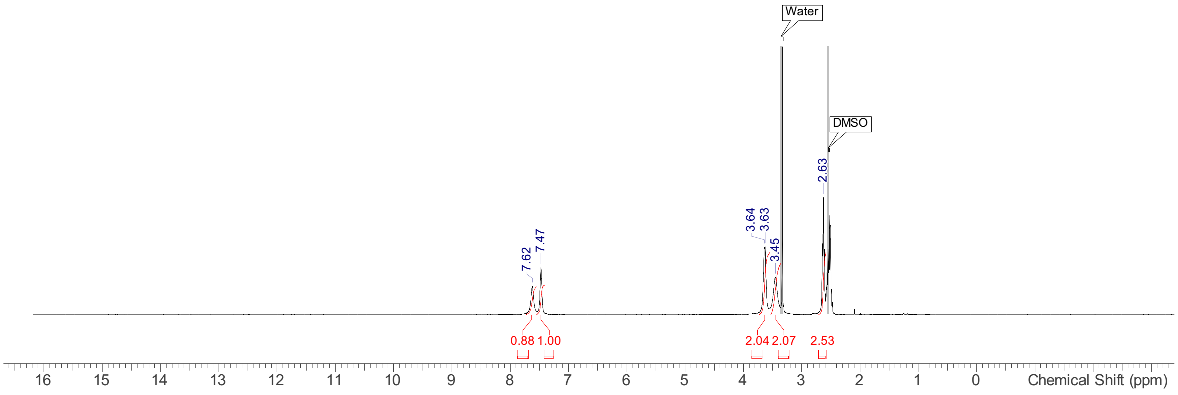

Figure S2.19 ^{1}H NMR (400 MHz, DMSO-d_6) spectrum of 1,1',1''-(nitrilotris(ethane-2,1-diyl)) tris(3-(3,3,3-trifluoropropyl)thiourea) **9**.

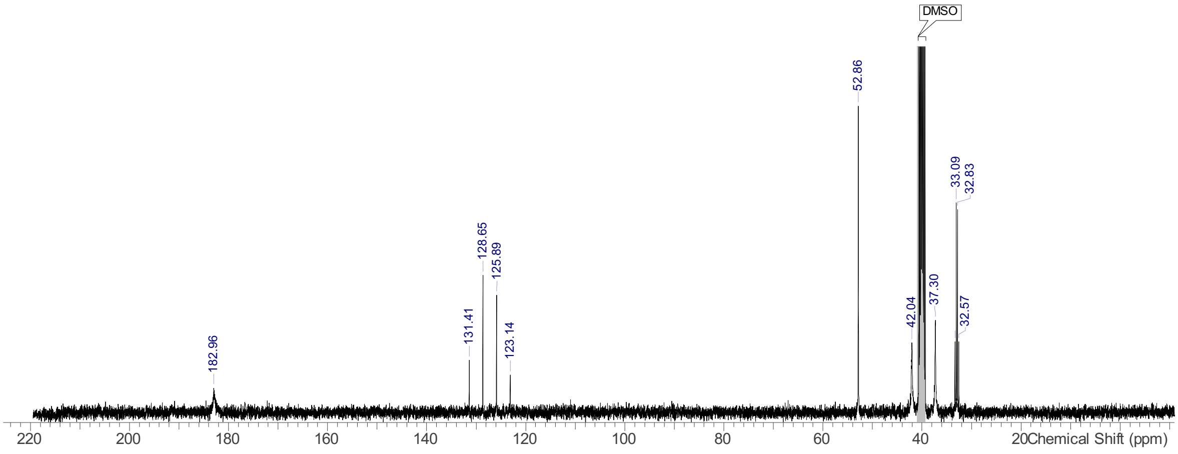

Figure S2.20 ^{13}C NMR (101 MHz, DMSO-d_6) spectrum of 1,1',1''-(nitrilotris(ethane-2,1-diyl)) tris(3-(3,3,3-trifluoropropyl)thiourea) **9**.

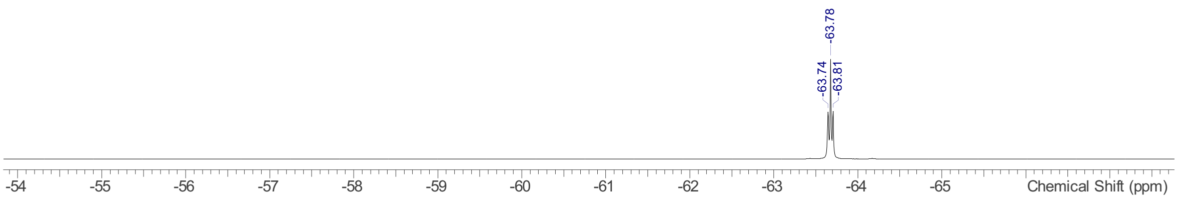

Figure S2.21 ^{19}F NMR (376 MHz, DMSO-d_6) spectrum of 1,1',1''-(nitrilotris(ethane-2,1-diyl)) tris(3-(3,3,3-trifluoropropyl)thiourea) **9**.

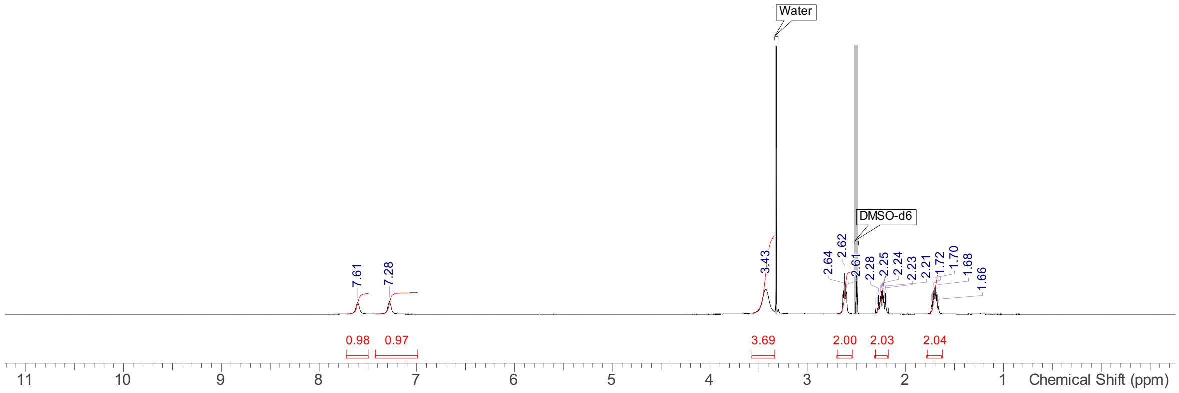

Figure S2.22 ^{1}H NMR (400 MHz, DMSO-d_6) spectrum of 1,1',1''-(nitrilotris(ethane-2,1-diyl))tris(3-(4,4,4-trifluorobutyl)thiourea) **10**.

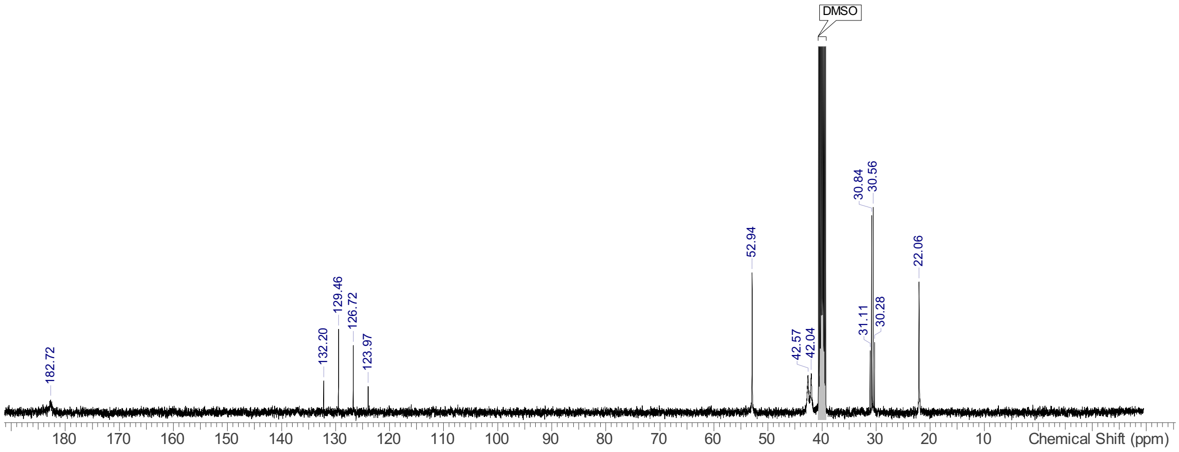

Figure S2.23 ^{13}C NMR (101 MHz, DMSO-d_6) spectrum of 1,1',1''-(nitrilotris(ethane-2,1-diyl))tris(3-(4,4,4-trifluorobutyl)thiourea) **10**.

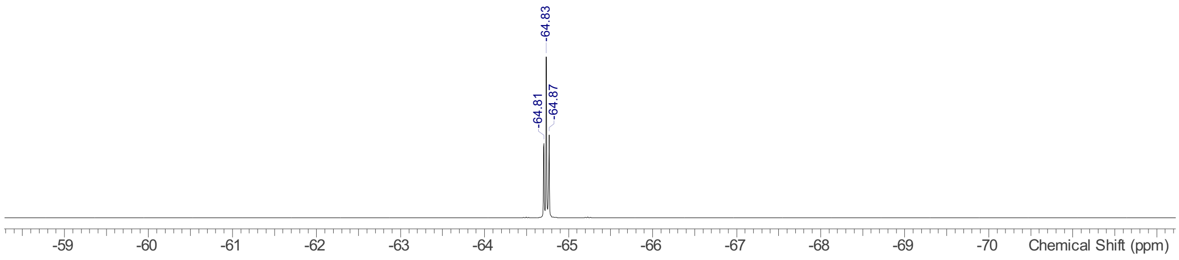

Figure S2.24 ^{19}F NMR (376 MHz, DMSO-d_6) spectrum of 1,1',1''-(nitrilotris(ethane-2,1-diyl)) tris(3-(4,4,4-trifluorobutyl)thiourea) **10**.

S3 Experimental Methods

S3.1 Transport Studies

S3.1.1 Lipid Vesicle Synthesis

POPC was purchased from Avanti Polar Lipids and stored as a solution in chloroform (1 g lipid in 35 ml). POPC solution was added to a 50 ml round-bottomed flask of known mass and the solvent removed under reduced pressure forming a lipid film. The film was dried *in vacuo* for at least 4 hours and the mass of lipid weighed.

The lipid film was re-suspended in a known quantity of internal buffer (INT) containing relevant salts for the experiment and buffered to the required pH. This suspension was subjected to 9 freeze-thaw cycles in liquid nitrogen before being allowed to rest for 30 minutes. The suspension was extruded 25 times through a 200 nm polycarbonate membrane forming monodispersed unilamellar vesicles. Buffer not encased by the vesicles was replaced by the relevant external buffer (EXT) by either dialysis or size-exclusion chromatography, as appropriate for the individual experiment (see below).

S3.1.2 NMDG/HPTS Assays

Internal buffer (INT): 100 mM NaCl, 10 mM HEPES buffer, 1 mM HPTS, pH 7.2
External buffer (EXT): 100 mM NaCl, 10 mM HEPES buffer, pH 7.2

Un-encapsulated HPTS was removed from extruded vesicles by size-exclusion chromatography on a Sephadex G-50 column using EXT as the eluent. 1 mM samples of vesicles suspended in EXT were loaded into an Agilent Technologies Carey Eclipse Fluorescence Spectrophotometer. The change in internal pH was monitored by recording the ratio between HPTS emission at 510 nm after excitation at 460 nm and 405 nm every 10 seconds.

At the start of the experiment any assisting protonophore was added to the sample if required as a 2 µl solution in DMSO (either Gramicidin-A to a final concentration of 0.1 % loading wrt. Lipid, or oleic acid to 2% loading). The compound to be tested was then added, again as 2 µl of an appropriately diluted solution in DMSO to the final concentration required. The experiment was initiated by addition of 20 µl of aqueous NMDG, to raise the external pH to pH 8.

After 300 s, a detergent was added (Triton X-100, Sigma-Aldrich, 1 g in 8 ml 7:1 H_2O:DMSO, 20 µl) to lyse the vesicles and calibrate to 100 % pH gradient dissipation. All trials were obtained in triplicate and the curves averaged. Curves were fitted to either a single exponential (Eq 1) or a double exponential function (Eq 2) using Origin 2015. This allowed the calculation of the initial transport rate at t = 0 as shown.

$$y = y_1 - ae^{-bx} \qquad \Rightarrow Init. Rate = ab \qquad \text{(Eq 1)}$$

$$y = y_1 - ae^{-bx} - ce^{-dx} \qquad \Rightarrow Init. Rate = ab + cd \qquad \text{(Eq 2)}$$

S3.2 **NMR Titrations**

All salts obtained from Sigma-Aldrich and dried under vacuum overnight prior to use. A solution of the sample receptor was prepared to a concentration of approximately 1 mM in 0.5 % H_2O/DMSO-d_6. 500 µl of the initial sample was placed in an NMR tube and the ^{1}H spectrum recorded using Bruker AVII400 FT-NMR spectrometer at 298 K. Aliquots of an ~16 mM solution of the TBA (tetrabutylammonium) salt of the anionic guest (dissolved in receptor sample solution) were added and ^{1}H NMR spectra obtained for each titre. Approximately 20 spectra were obtained, with titres from 0 to ~6 equivalents of anion to receptor.

The chemical shifts of both NH protons were globally fitted using BindFit v0.5.[4, 5] The data were fitted to a 1:1 binding model giving binding constants with asymptotic errors all < 3 %.

S3.3 **Computational Methods**

The quantum calculations were carried out with Gaussian 09 (Rev. A.02),[6] using different theory levels, as further detailed. These calculations include the geometry optimisations of the oleate anion (OA), the free tripodal derivatives and their chloride complexes and calculation of the distribution of electrostatic potential. The distribution of the electrostatic potential, *V*(r), on the molecular surface of the tripodal compounds was computed from previously optimised structures of their chloride complexes, after removal of the anion, using the same level of theory. Subsequently, the *V*(r) was evaluated on the 0.001 electrons Bohr^{-3} contour of ρ(r), and is henceforth labelled V_S(r). The electrostatic potential surface ranges, including the most negative and most positive values ($V_{S,min}$ and $V_{S,max}$, respectively), were ascertained using the *Multiwfn* software.[7]

The theoretical investigation on membrane systems comprised passive diffusion MD simulations, Steered Molecular Dynamics (SMD) and Umbrella Sampling (US) simulations. All MD simulations were carried out with AMBER 16,[8] with resort to GPU acceleration.[9-11] The LIPID14[12] force field was employed for the POPC lipids, while GAFF[13, 14] parameters and atomic RESP charges[15] were used for the tripodal molecules and oleate anion, as follows.

S3.3.1 Structure Generation

The initial structures of free **1** - **10** were generated by atomic manipulation of the crystal structure of **3**·Cl^-. Subsequently, these structures were optimised at the HF/6-31G* level with the Gaussian 09 software.[6] The optimisation of these ten individual structures was followed by a single point

calculation to generate the electrostatic potential (ESP) at the same theory level, using the Merz-Singh-Kollman scheme with 4 concentric layers per atom and 6 density points in each layer (IOp(6/33=2, 6/41=4, 6/42=6)). The initial atomic charges of each molecule were then calculated by RESP fitting, along with the assignment of GAFF atom types, using the *antechamber* module, as implemented in the AMBER software suite.[16]

To obtain atomic charges less dependent on molecular conformation or orientation and the calculation of the final RESP charges of **1** - **10** employed in all membrane MD simulations, conformational analyses were undertaken on the tripodal compounds using the following protocol: The initial molecular mechanics (MM) energy minimised structures, using the initial RESP charges, were heated to 500 K in the gas phase for 50 ps, followed by collection runs of 0.5 ns using a time step of 1 fs, allowing the stochastic search of the conformational space. Frames were saved every 0.1 ps leading to trajectory files containing 5000 structures. All these structures were further minimised by MM using the steepest descendent gradient followed by the conjugate gradient algorithm, until the convergence criterion of 0.0001 kcal mol^{-1} $Å^{-1}$ was attained. Afterwards, the MM minimised structures were clustered with the UCSF Chimera software.[17] From the resulting clusters, three representative conformations with substantially different RMSD values were selected for each tripodal molecule. All selected conformations had the N−H binding units of the thiourea moieties adopting a *syn* configuration, but different spatial dispositions of the thiourea appended chains. All these structures were further HF/6-31G* geometry optimised, followed by their ESP calculations as described above. Then, the individual ESP data were extracted from the corresponding Gaussian 09 outputs, concatenated and subsequently used to generate the input files for the two-stage RESP fitting, using identical weights for all conformations. On the other hand, for the oleate anion, eight structures were generated from the POPC lipids taken from an equilibrated bilayer. Afterwards, these randomly selected structures were optimised at the HF/6-31G* level followed by ESP calculations. As previously described, the ESP data were used to generate the final multi-RESP atomic charges.

The structures of the chloride complexes of **1** - **10**, also generated from the X-ray crystal structure of **3**·Cl^-, were DFT optimised in gas-phase at the M06-2X/6-31+G(d,p) level of theory. All optimised geometries have been characterised as minima by the absence of imaginary frequencies. The quantum optimised structures of the tripodal chloride complexes were used in further single point quantum calculations, as well as starting binding arrangements in MD simulations with a POPC bilayer model. The structure of **6**·OA was generated with the carboxylate group positioned inside the tripodal binding pocket. This initial geometry was optimised *via* DFT at the same level of theory, and the final structure was used in the ensuing MD simulations.

S3.3.2 MD Simulations

The chloride or oleate anion complexes were inserted in a free POPC bilayer with Packmol,[18] at its core (scenario A) or in its water slab (scenario B). This previously equilibrated membrane system is a POPC membrane model, with an orthorhombic shape composed of 128 phospholipids, 6500 TIP3P model water molecules,[19] and 17 Cl^- and 18 Na^+ solvated ions (*ca.* 0.15 M). The monoatomic ions were described with suitable van der Waals parameters.[20]

The MD simulations with the chloride complexes were carried out as follows: the initial configuration of each system was subjected to 20,000 steps of MM energy minimisation with a 500 kcal mol^{-1} $Å^{-2}$ positional restraint on the chloride complex and lipid molecules, through the steepest descent algorithm for 10,000 steps plus 10,000 steps of the conjugated gradient algorithm. Subsequently, the restraints were removed and the entire system was allowed to relax for another 20,000 steps, using the same algorithms. The system was equilibrated by heating it to 303 K in an NVT ensemble for 100 ps with a 10 kcal mol^{-1} $Å^{-2}$ restraint on the chloride complex and lipid molecules followed by a 5 ns run using an NPT ensemble with a 5 kcal mol^{-1} $Å^{-2}$ restraint on the chloride complex. Then, the positional restraint was removed and the simulation continued for a further 300 ns (scenario A, a single MD run) or 200 ns (scenario B, two independent MD runs). Long-range electrostatic interactions were described with the Particle Mesh Ewald (PME) algorithm[21] using a real-space cut-off at 10 Å. The cut-off for the Lennard-Jones interactions was also set at 10 Å. The temperature of the system was maintained at 303 K, using the Langevin thermostat,[22] with a collision frequency γ of 1.0 ps^{-1}. The pressure was controlled by the Berendsen barostat[23] at 1 atm and compressibility of 44.6×10^{-6} bar^{-1}, with a relaxation time of 1.0 ps. The covalent bonds to hydrogen atoms were constrained using the SHAKE algorithm,[24] allowing the use of a 2 fs time step. The MD simulation trajectory frames were saved every 10.0 ps. The **6**·OA complex was simulated using the same multi-stage equilibration protocol, but with shorter production runs of 100 ns in both scenarios. The post processing and analysis of the MD simulation trajectory files were performed with *cpptraj*.[25]

The starting configurations necessary for the US simulations of free **2**, **6**, **8** and **9** were generated from previous SMD simulations, as follows: the complexed chloride in scenario A was relocated to the water phase and then the system was minimised, heated, and equilibrated as described above. Afterwards, the final frame of the equilibration period was used as the starting frame for the SMD. Subsequently, the free receptors were dragged from the bilayer core, along the membrane normal (*viz*, *z*-dimension, the reaction coordinate) at 2.5 Å ns^{-1} in MD simulations of 17 ns, in the NPT ensemble. Throughout the travelled 42.5 Å along the membrane system, a force constant of 5.0 kcal mol^{-1} $Å^{-2}$ was applied to the non−hydrogen atoms of the tripodal derivatives. A similar procedure was also applied to **6** in scenario B: the complexed anion was relocated to the water phase and the system was minimised, heated and equilibrated as above, prior to the SMD simulation where the tripodal molecule was dragged along the

membrane normal (the *z*-dimension) for 170 ns at 0.5 Å ns^{-1}, with a force constant of 5.0 kcal mol^{-1} $Å^{-2}$. This methodology resulted in the exit of the free tripodal receptors to the water phase having a small structural impact on the POPC bilayer but avoiding the formation of a chloride complex below the water/lipid interface.

On the other hand, the starting geometries for the tripodal complexes (both chloride and OA) were gathered from the passive diffusion MD simulations carried out in scenarios A and B. The spacing between the centre of mass of the tripodal molecules in the US simulations is *ca.* 1 Å, with a total of 31 evenly spaced independent starting points (ranging from 0 to +30 Å along the *z* axis). A distance restraint of 5.0 kcal mol^{-1}·$Å^{-2}$ was applied along the *z*-dimension, between the centre of mass of the non-hydrogen atoms of the tripodal receptor and the centre of mass defined by phosphorus atoms of the lipid headgroups and the terminal CH_3 carbon atoms. Each of the 31 windows was simulated for 80 ns, except for the chloride complexes of **6** and **8**, which were run for 100 ns, and the US MD simulations of **6**·OA, which were 130 ns long. Only the last 50 ns were considered as sampling time, with the initial MD simulation period being discarded as equilibration period. In the US simulations of the tripodal complexes, six 5.0 kcal mol^{-1} $Å^{-2}$ harmonic distance restrains between the nitrogen atoms and the chloride or carboxylate carbon atom were applied. Each independent US window underwent a minimisation and heating stage, with the same distance restraints of the production run. The remaining details are as given for the passive diffusion simulations, apart from the distance restraints. The trajectory frames were saved every 10 ps, while the distance between the centre of mass of the tripodal derivative and the centre of mass of the POPC bilayer, defined by the phosphorus atoms of the lipid head groups and the terminal CH_3 carbon atoms, was saved every 50 steps.

The US simulations of the single Cl^- were preceded by a SMD simulation, carried out using as starting point a frame of the equilibrated free membrane. A solvated chloride ion was pulled along the bilayer normal at 5.0 Å ns^{-1} in a 16 ns long MD simulation in the NPT ensemble. A force constant of 5.0 kcal mol^{-1} $Å^{-2}$ was applied to the anion along the 80 Å of the membrane system. In the corresponding US simulations, the free anion was restrained with a 3.0 kcal mol^{-1} $Å^{-2}$ force applied along the *z*-dimension, and the 31 independent windows were simulated for 25 ns, with only the final 10 ns being considered for further analysis.

S3.3.3 Potential of Mean Force calculations

The free energy profiles were estimated using the variational free energy profile (VFEP) method,[26, 27] from the last 50 ns of the US MD simulations with **2**, **6**, **8** and **9**. The individual profiles, plotted with the bootstrap errors calculated from 100 random data sets with the same size, are shown in Figures S4.29 and S4.30. All profiles were normalised for z = 30 Å (bulk water). The energy profiles are also

plotted together in the main text for the chloride complexes and free anion (Fig. 10A), and for the free transporters (Fig. 10B).

S3.3.4 Interaction Energy Calculations

The interaction energies between the POPC bilayer and the tripodal molecules **2**, **6**, **8** & **9** were energetically evaluated through (Eq 3):

$$\Delta E_{total} = \Delta \mathrm{E}_{elec} + \Delta E_{vdW} + \Delta E_{int} \qquad \text{(Eq 3)}$$

ΔE_{elec} and ΔE_{vdW} correspond to the non-bonded electrostatic and van der Waals energy terms, while ΔE_{int} corresponds to the sum of bond, angle and dihedral energies. The three individual molecular mechanics energy components are given by (Eq 4 – 6):

$$\Delta E_{elec} = \Delta \mathrm{E}_{elec}(system) - (\Delta \mathrm{E}_{elec}(POPC) + \Delta \mathrm{E}_{elec}(T)) \qquad \text{(Eq 4)}$$

$$\Delta E_{vdW} = \Delta \mathrm{E}_{vdW}(system) - (\Delta \mathrm{E}_{vdW}(POPC) + \Delta \mathrm{E}_{vdW}(T)) \qquad \text{(Eq 5)}$$

$$\Delta E_{int} = \Delta \mathrm{E}_{int}(system) - (\Delta \mathrm{E}_{int}(POPC) + \Delta \mathrm{E}_{int}(T)) \qquad \text{(Eq 6)}$$

Here $(system)$ represents the transporter and the phospholipid membrane, excluding the water molecules and all chloride and sodium ions. $(POPC)$ stands for the 128 phospholipid molecules and (T) for the isolated tripodal molecules (**2**, **6**, **9** or **8**). These three energies were estimated with snapshots extracted every 100 ps from the last 50 ns of the US window corresponding to the *z*-dimension of the minimum in energy profile of the corresponding transporter.

The bonded term ΔE_{int} amounts to zero, given that the individual terms $\Delta \mathrm{E}_{int}(system)$, $\Delta \mathrm{E}_{int}(POPC)$ and $\Delta \mathrm{E}_{int}(T)$ were calculated using the same MD simulation. In these conditions, through (Eq 3), only the contribution of both non-bonded energy terms is evaluated in the intermolecular interactions between the phospholipids and transporters. The average values of ΔE_{total}, $\Delta \mathrm{E}_{elec}$ and $\Delta \mathrm{E}_{vdW}$ for the relevant US MD simulations are given in Table S4.3.

S3.4 **Anionophore-Mediated Anion Transport in Cells**

S3.4.1 Cells and cell culture

Fischer rat thyroid (FRT) cells stably expressing the halide sensor YFP-H148Q/I152L (YFP-FRT cells)[28, 29] were a generous gift of AS Verkman (University of California, San Francisco). YFP-FRT cells were cultured as described previously.[30] For anion transport studies, YFP-FRT cells were seeded into 96-well plates and used for anion transport studies 3-4 days later.

S3.4.2 Reagents

Stocks of anionophores were prepared in DMSO. Immediately before use, stock solutions were diluted in phosphate buffered saline (PBS) to achieve final concentrations. By itself, the vehicle DMSO (0.1 or 0.5 % v v^{-1}), was without effect on YFP fluorescence.

S3.4.3 Anionophore-mediated anion transport

Anionophore-mediated anion transport was quantified by measuring I^--induced quenching of YFP fluorescence using a microplate reader (model: FLUORstar Omega; BMG LABTECH Ltd., Aylesbury, UK) following the method of Galietta et al.[29] In brief, after washing YFP-FRT cells twice with PBS, they were incubated with anionophores for 10 minutes at 37 °C before I^- (100 mM) was added at 37 °C to quench YFP fluorescence. The rate of I^--induced quenching, determined by the fit of first-order exponential functions, was used to quantify anion transport by test anionophores.

S3.4.4 Statistics

Anionophores were tested in duplicate on each 96-well plate in at least three independent experiments. Results are expressed as means ± SEM of n observations. To compare sets of data, we used Student's unpaired t-test. Differences were considered statistically significant when $P < 0.05$. All tests were performed using SigmaPlot 12 (Systat Software Inc., San Jose, CA, USA).

S4 Supplementary Data

S4.1 Lipophilicity Estimation

Log P values were determined experimentally using the retention factor (k') from isocratic HPLC retention time (RT) data, as this is known to be directly proportional to log P.[31, 32] Retention times were measured in triplicate on a C_{18} RP-HPLC column with isocratic elution with 50 % CH_3CN/H_2O. Although calibration of retention times to a known series of compounds can be used to convert k' to log P[31], the expected large range of log P values in this series of compounds made this impractical. k' is calculated from RT using (Eq 7), where t_0 is the column dead time, measured by the retention time of an un-retained polar compound (urea, RT = 0.22 min) and confirmed by calculation from RT data using a homologous series of alkyl substituted thoiureas[33] and (Eq 8).[31]

$$k' = \frac{RT - t_0}{t_0} \qquad \text{(Eq 7)}$$

$$RT(N_{C+1}) = A * RT(N_C) + (1 - A)t_0 \qquad \text{(Eq 8)}$$

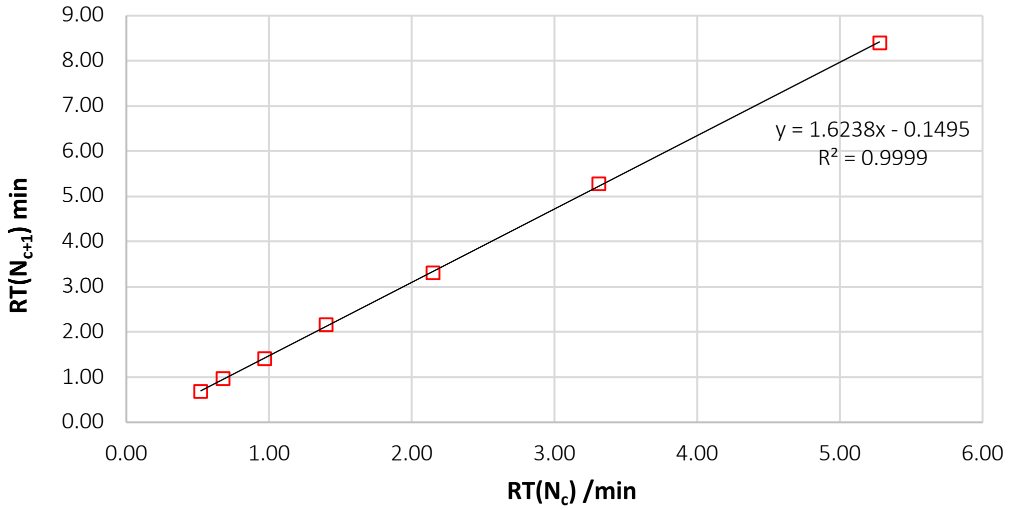

Figure S4.1 Linear fit of RT data of a homologous series of alkyl substituted thioureas [33]. Linear regression yields A = 1.623807, $(1\text{-}A)t_0$ = -0.149526 giving a value of t_0 of 0.22 min.

Table S4.1 Log (k') calculations for compounds **1** – **10**. RT = retention time in min, triplicate runs. RT Av = average of RT triplicates, RT SD = standard deviation of average values. k` calculated as per (Eq 7); ± error on log values calculated by best/worst cases based on standard deviations on RT Av values.

Compound	RT 1	RT 3	RT 2	RT Av	RT SD	k`	Log (k`)	± Error
**1**	0.22	0.22	0.23	0.22	0.01	0.0152	-1.82	0.44
**2**	0.25	0.26	0.24	0.25	0.01	0.1364	-0.87	0.12
**3**	0.38	0.38	0.38	0.38	0.00	0.7273	-0.14	0.00
**4**	0.85	0.85	0.82	0.84	0.02	2.8106	0.45	0.01
**5**	0.30	0.30	0.30	0.30	0.00	0.3636	-0.44	0.00
**6**	0.63	0.63	0.62	0.63	0.01	1.8485	0.27	0.01
**7**	2.05	2.06	2.05	2.05	0.01	8.3333	0.92	0.00

**8**	7.61	7.59	7.65	7.62	0.03	33.6212	1.53	0.00
**9**	0.34	0.34	0.33	0.34	0.01	0.5303	-0.28	0.02
**10**	0.42	0.42	0.43	0.42	0.01	0.9242	-0.03	0.01

S4.2 NMDG Assay Transport Curves

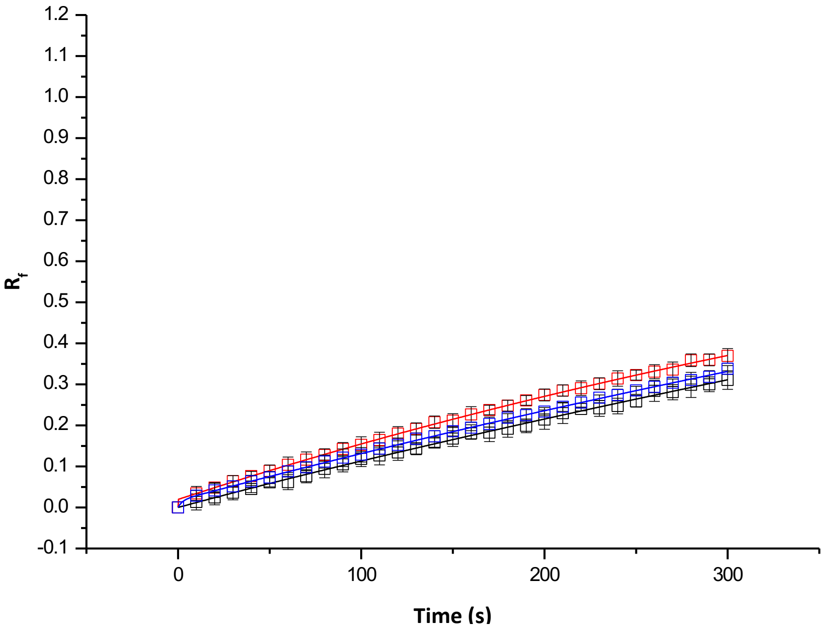

Figure S4.2 Relative fluorescence trace from HPTS assays for compound **1** at 0.01 % loading with fitted exponential function curves. Black squares: no assisting protonophore; red squares: 0.1 mol % Gramicidin-A; blue squares: 2 mol % oleic acid.

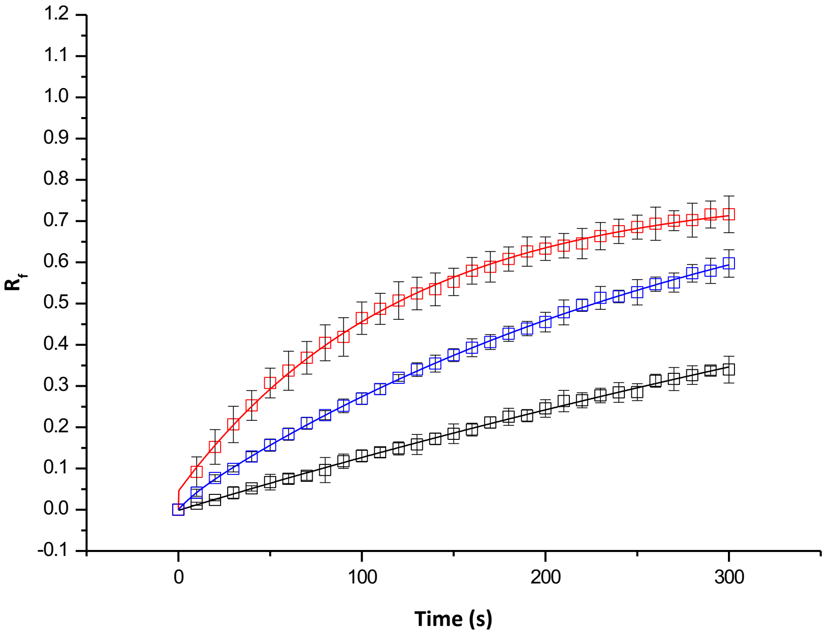

Figure S4.3 Relative fluorescence trace from HPTS assays for compound **2** at 0.01 % loading with fitted exponential function curves. Black squares: no assisting protonophore; red squares: 0.1 mol % Gramicidin-A; blue squares: 2 mol % oleic acid.

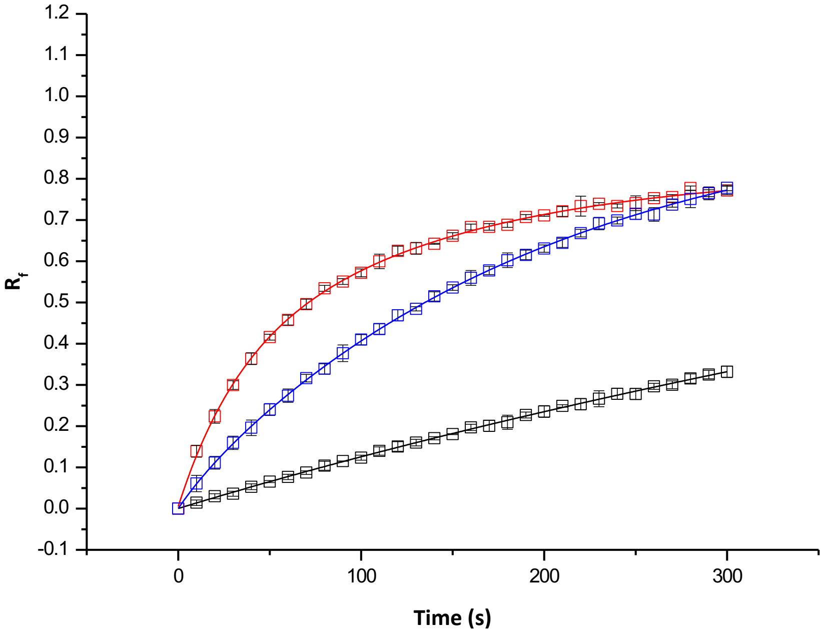

Figure S4.4 Relative fluorescence trace from HPTS assays for compound **3** at 0.01 % loading with fitted exponential function curves. Black squares: no assisting protonophore; red squares: 0.1 mol % Gramicidin-A; blue squares: 2 mol % oleic acid.

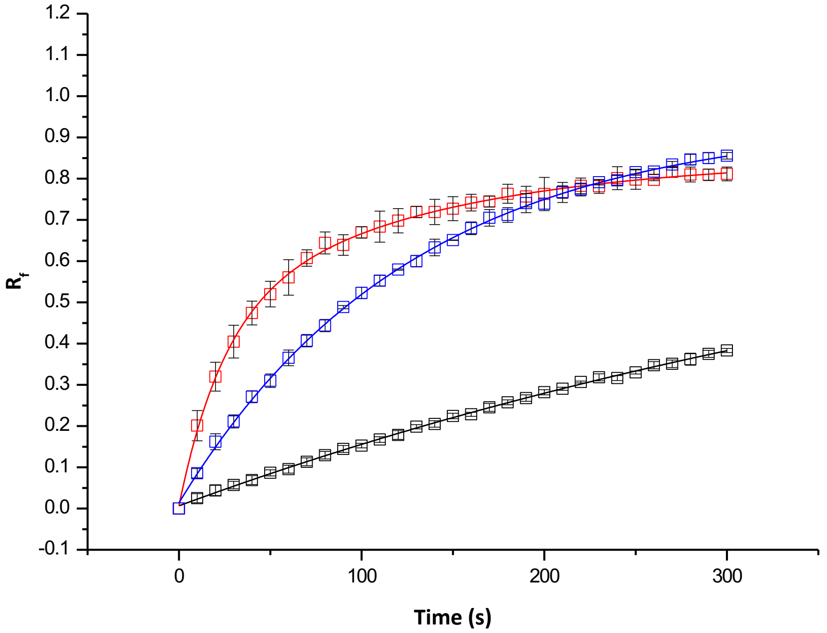

Figure S4.5 Relative fluorescence trace from HPTS assays for compound **4** at 0.01 % loading with fitted exponential function curves. Black squares: no assisting protonophore; red squares: 0.1 mol % Gramicidin-A; blue squares: 2 mol % oleic acid.

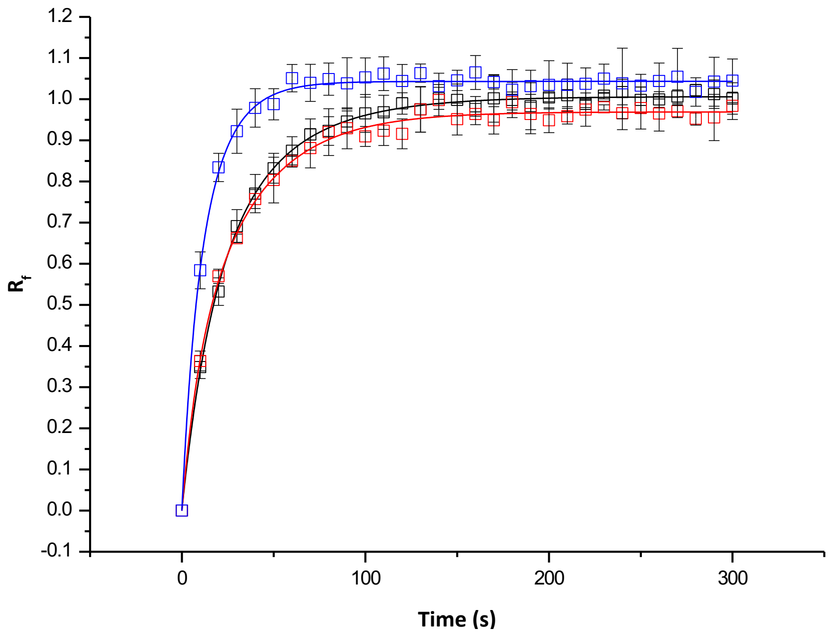

Figure S4.6 Relative fluorescence trace from HPTS assays for compound **5** at 0.01 % loading with fitted exponential function curves. Black squares: no assisting protonophore; red squares: 0.1 mol % Gramicidin-A; blue squares: 2 mol % oleic acid.

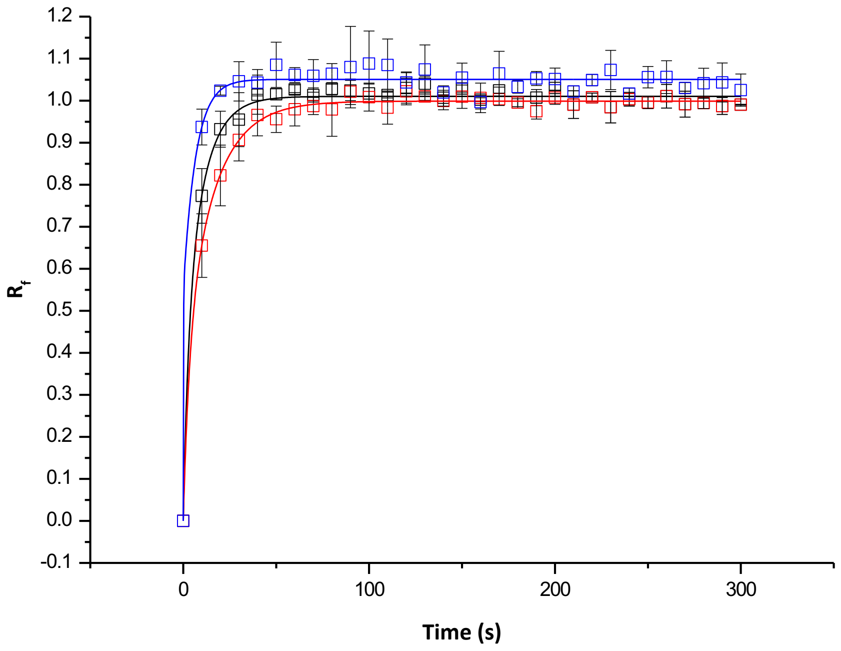

Figure S4.7 Relative fluorescence trace from HPTS assays for compound **6** at 0.01 % loading with fitted exponential function curves. Black squares: no assisting protonophore; red squares: 0.1 mol % Gramicidin-A; blue squares: 2 mol % oleic acid.

As can be seen in Figure S4.7, compound **6** is highly active in the NMDG-Cl assay at 0.01 % loading preventing accurate determination of initial rate values. Thus, compound **6** was studied at 0.001 % loading, the curves for which are presented in **Figure S4.8.**

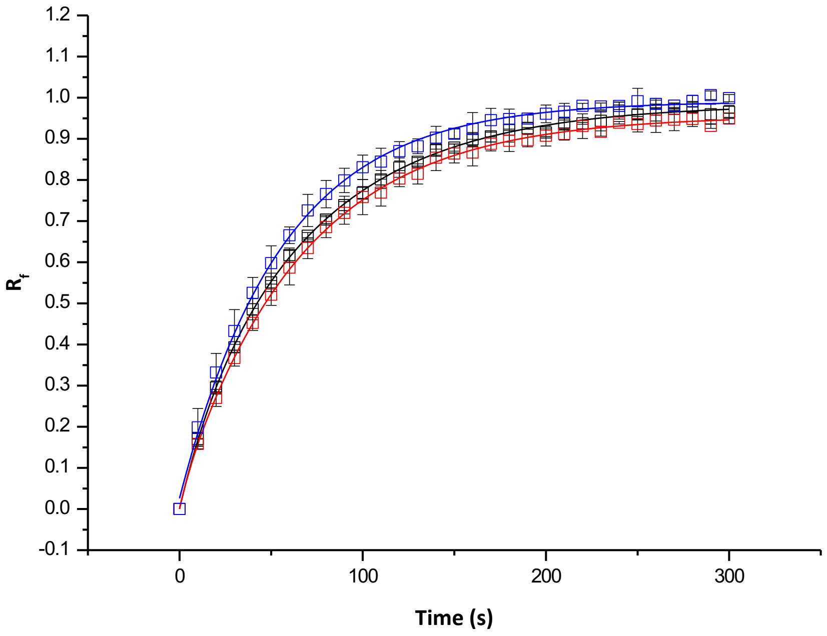

Figure S4.8 Relative fluorescence trace from HPTS assays for compound **6** at 0.001 % loading with fitted exponential function curves. Black squares: no assisting protonophore; red squares: 0.1 mol % Gramicidin-A; blue squares: 2 mol % oleic acid.

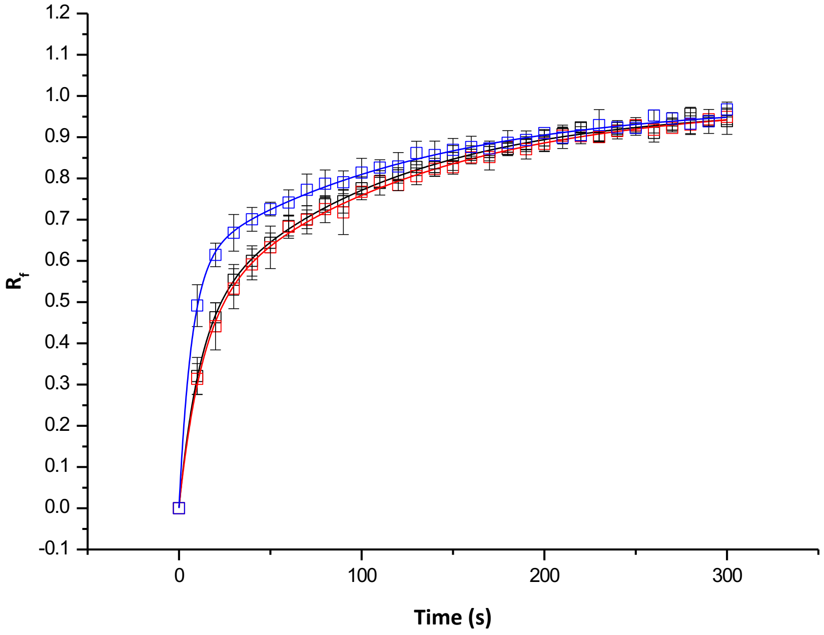

Figure S4.9 Relative fluorescence trace from HPTS assays for compound **7** at 0.01 % loading with fitted exponential function curves. Black squares: no assisting protonophore; red squares: 0.1 mol % Gramicidin-A; blue squares: 2 mol % oleic acid.

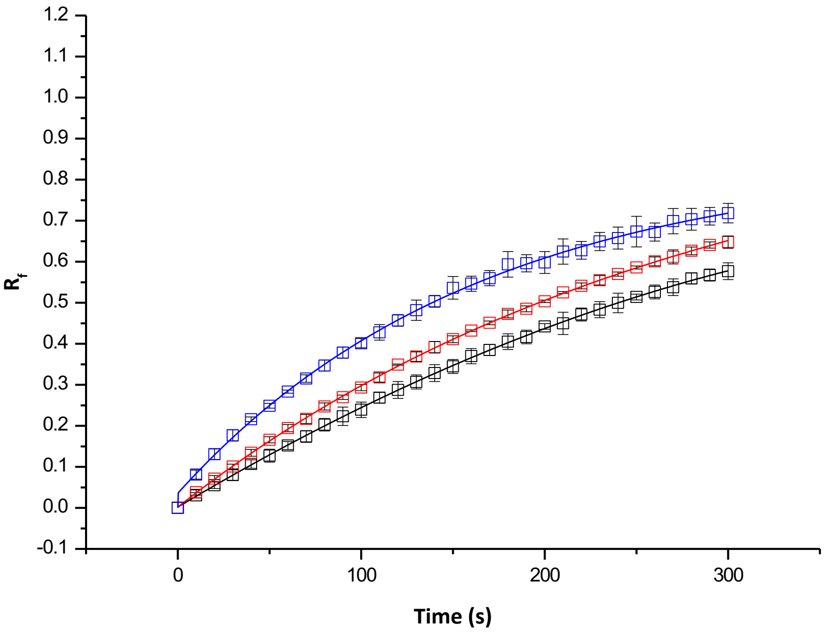

Figure S4.10 Relative fluorescence trace from HPTS assays for compound **8** at 0.01 % loading with fitted exponential function curves. Black squares: no assisting protonophore; red squares: 0.1 mol % Gramicidin-A; blue squares: 2 mol % oleic acid.

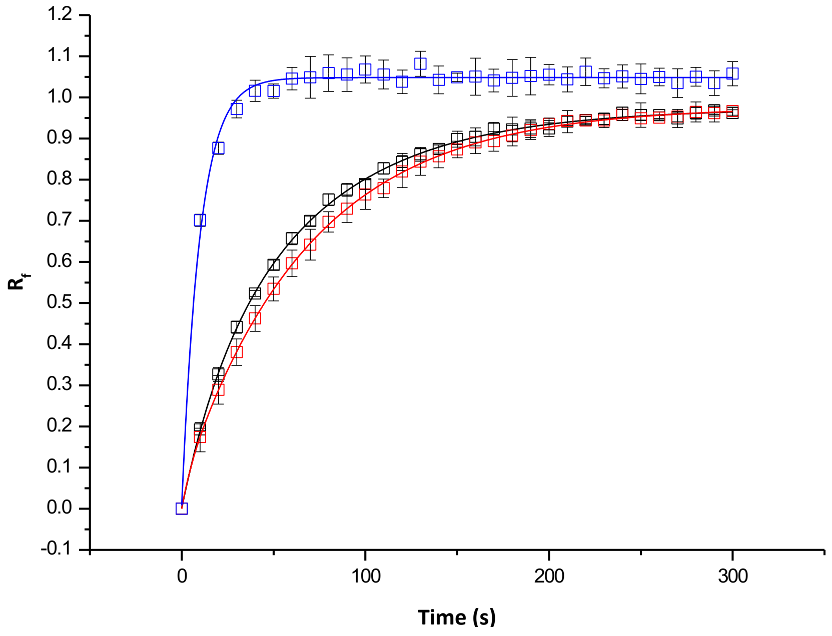

Figure S4.11 Relative fluorescence trace from HPTS assays for compound **9** at 0.01 % loading with fitted exponential function curves. Black squares: no assisting protonophore; red squares: 0.1 mol % Gramicidin-A; blue squares: 2 mol % oleic acid.

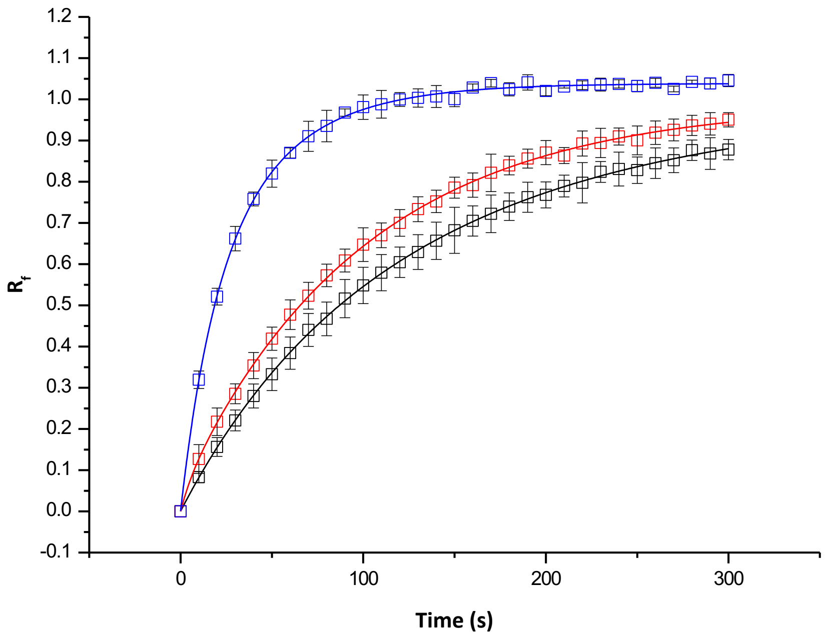

Figure S4.12 Relative fluorescence trace from HPTS assays for compound **10** at 0.01 % loading with fitted exponential function curves. Black squares: no assisting protonophore; red squares: 0.1 mol % Gramicidin-A; blue squares: 2 mol % oleic acid.

S4.3 **NMR Titration Fit Plots**

The following fits were obtained for the tren series using global fitting with BindFit v0.5[4, 5].

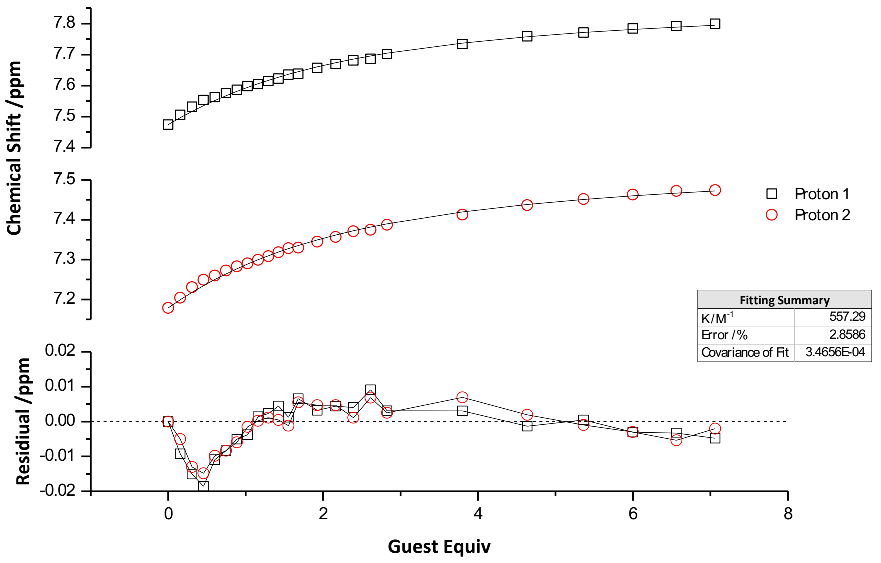

Figure S4.13 Plot of 1',1''-(nitrilotris(ethane-2,1-diyl))tris(3-ethylthiourea) **1** with TBA Cl, following both NH protons (δ = 7.47, 7.18 ppm). From the fitted functions, K = 557 M^{-1}, error = 2.9 %.

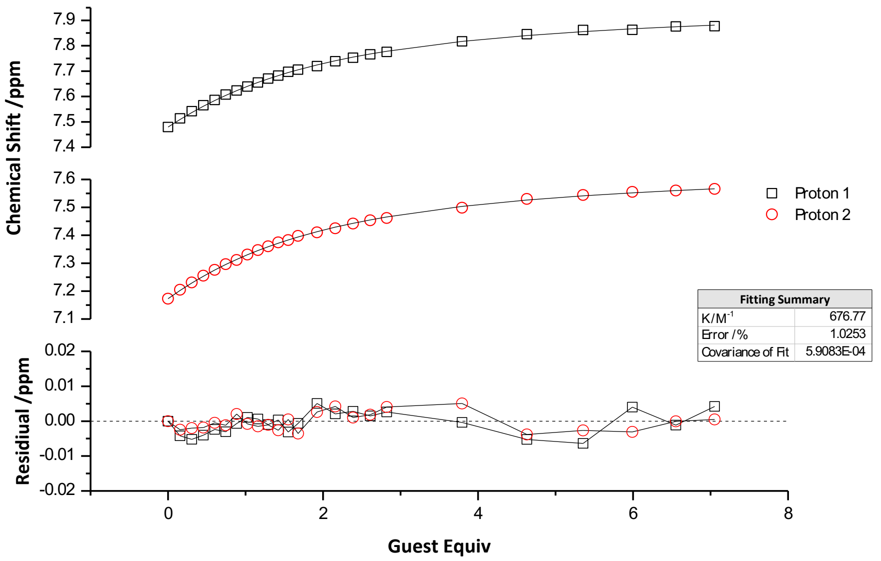

Figure S4.14 Plot of 1',1''-(nitrilotris(ethane-2,1-diyl))tris(3-propylthiourea) **2** with TBA Cl, following both NH protons (δ = 7.48, 7.17 ppm). From the fitted functions, K = 677 M^{-1}, error = 1.0 %.

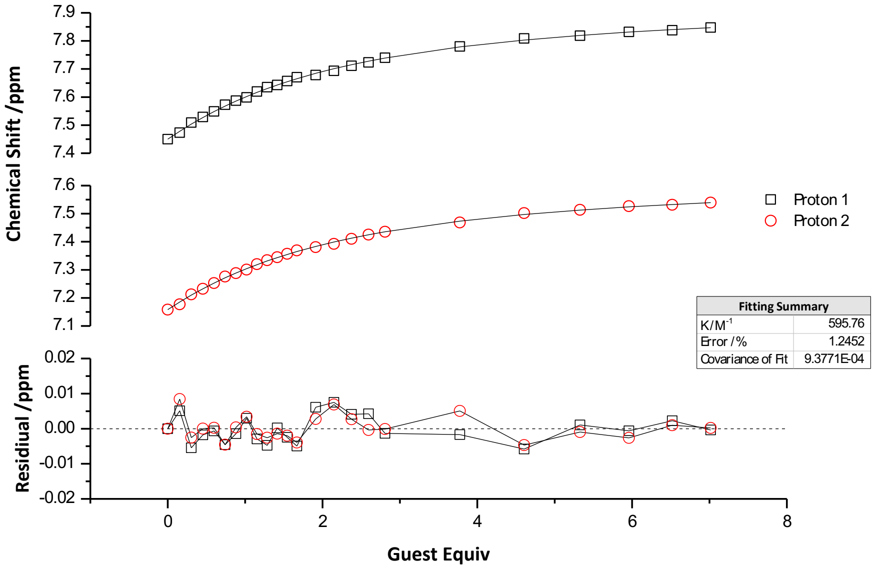

Figure S4.15 Plot of 1',1''-(nitrilotris(ethane-2,1-diyl))tris(3-butylthiourea) **3** with TBA Cl, following both NH protons (δ = 7.45, 7.16 ppm). From the fitted functions, K = 596 M^{-1}, error = 1.2 %.

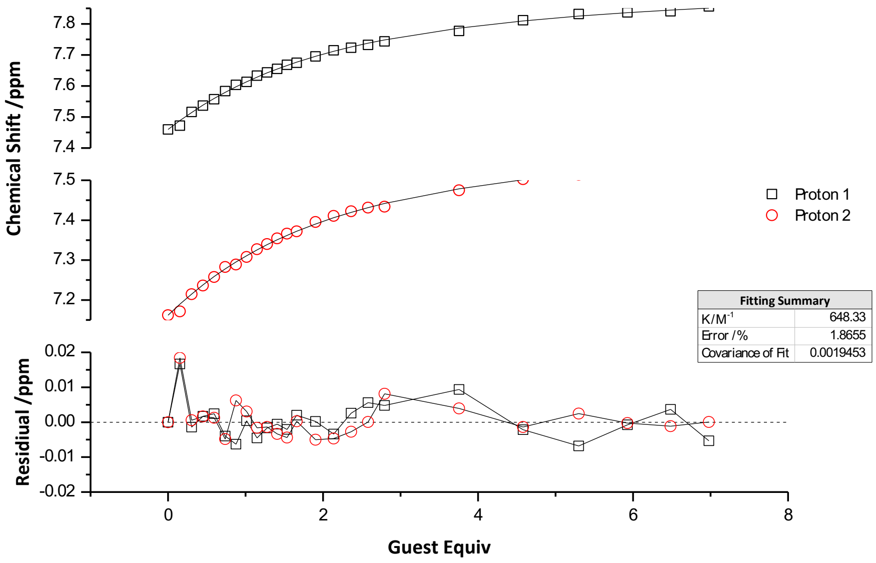

Figure S4.16 Plot of 1',1''-(nitrilotris(ethane-2,1-diyl))tris(3-pentylthiourea) **4** with TBA Cl, following both NH protons (δ = 7.46, 7.16 ppm). From the fitted functions, K = 648 M^{-1}, error = 1.92 %.

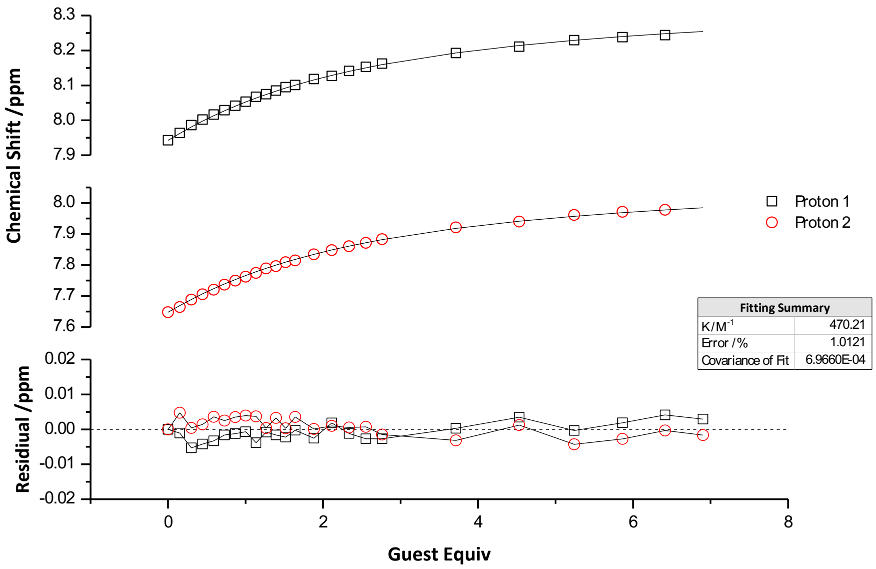

Figure S4.17 Plot of 1',1''-(nitrilotris(ethane-2,1-diyl))tris(3-(2,2,2-trifluoroethylthiourea **5** with TBA Cl, following both NH protons (δ = 7.94, 7.65 ppm). From the fitted functions, K = 470 M^{-1}, error = 1.0 %.

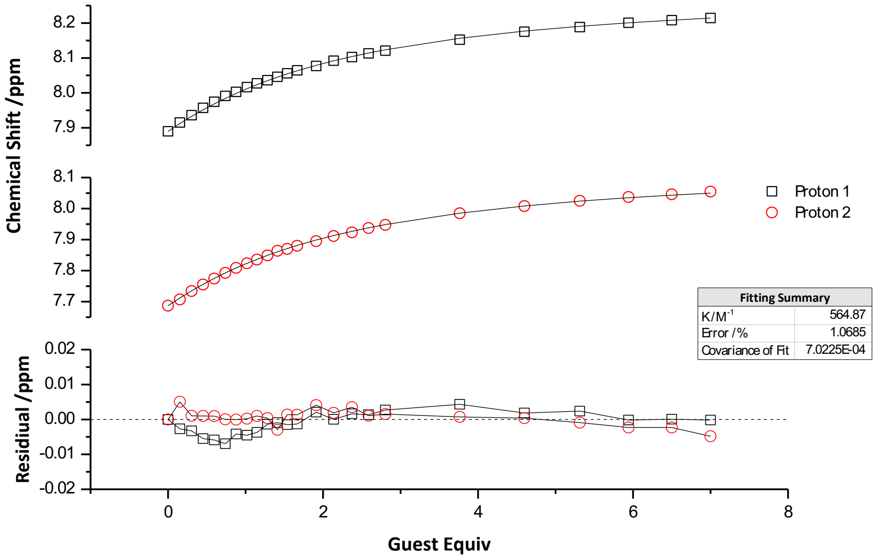

Figure S4.18 Plot of 1',1''-(nitrilotris(ethane-2,1-diyl))tris(3-(2,2,3,3,3-pentafluoropropylthiourea) **6** with TBA Cl, following both NH protons (δ = 7.89, 7.69 ppm). From the fitted functions, K = 565 M^{-1}, error = 1.1 %.

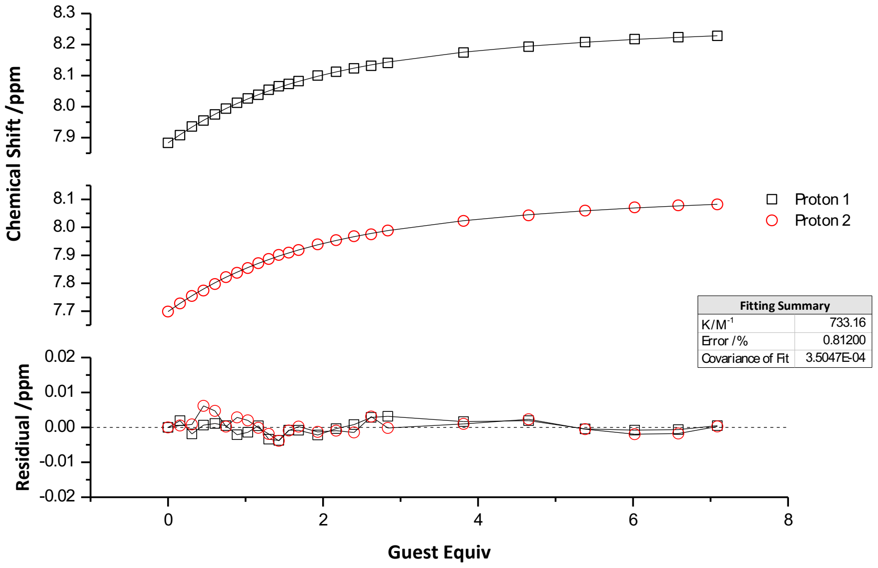

Figure S4.19 Plot of 1',1''-(nitrilotris(ethane-2,1-diyl))tris(3-(2,2,3,3,4,4,4-heptafluorobutylthiourea) **7** with TBA Cl, following both NH protons (δ = 7.88, 7.70 ppm). From the fitted functions, K = 733 M^{-1}, error = 0.8 %.

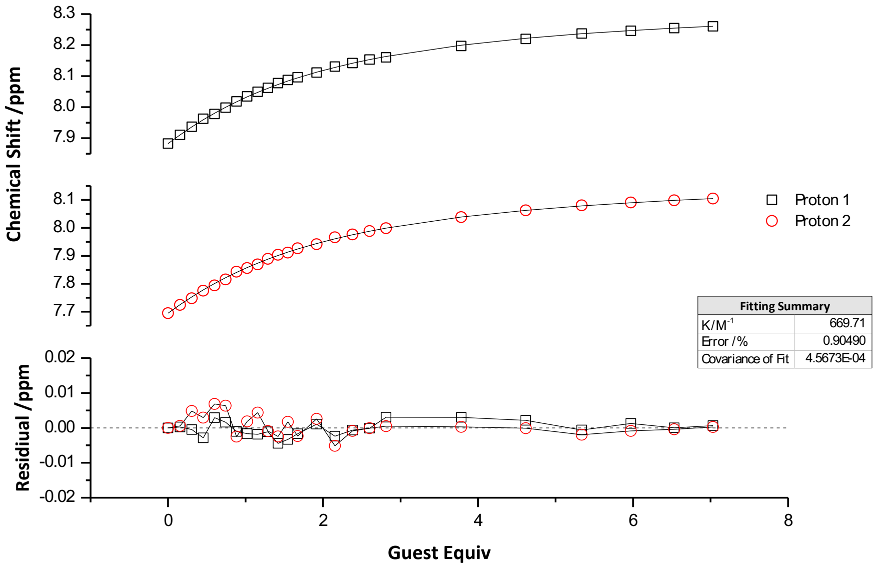

Figure S4.20 Plot of 1',1''-(nitrilotris(ethane-2,1-diyl))tris(3-(2,2,3,3,4,4,5,5,5-nonafluoropentylthiourea) **8** with TBA Cl, following both NH protons (δ = 7.88, 7.69 ppm). From the fitted functions, K = 670 M^{-1}, error = 0.9 %.

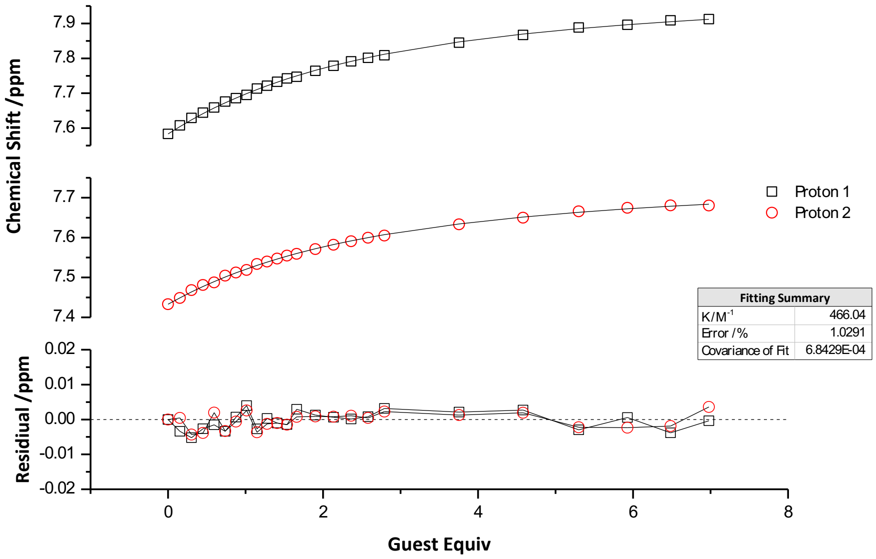

Figure S4.21 Plot of 1',1''-(nitrilotris(ethane-2,1-diyl))tris(3-(3,3,3-trifluoropropylthiourea) **9** with TBA Cl, following both NH protons (δ = 7.58, 7.43 ppm). From the fitted functions, K = 466 M^{-1}, error = 1.0 %.

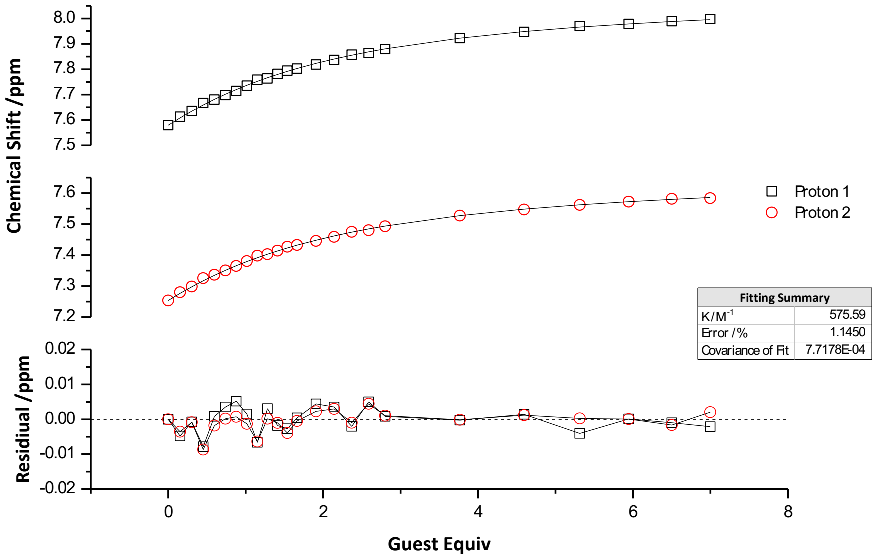

Figure S4.22 Plot of 1',1''-(nitrilotris(ethane-2,1-diyl))tris(3-(4,4,4-trifluorobutylthiourea) **10** with TBA Cl, following both NH protons (δ = 7.67, 7.33 ppm). From the fitted functions, K = 576 M^{-1}, error = 1.1 %.

S4.4 Additional Modelling Data

Table S4.3 Average molecular mechanics energy terms (kcal mol^{-1}), with the corresponding standard deviations, assessed during the last 50 ns of the US window at *z* = 10 Å for **2**, **6** and **9** or *z* = 8 Å for **8**. N = 500.

Compound	ΔE_{total}	ΔE_{elec}	ΔE_{vdW}
**2**	-77.47 ± 12.04	-21.03 ± 9.33	-56.44 ± 5.38
**6**	-103.57 ± 15.10	-37.21 ± 13.15	-66.37 ±4.98
**9**	-110.23 ± 24.18	-52.01 ± 21.68	-58.23 ± 5.51
**8**	-84.71 ± 15.10	-17.53 ± 11.00	-67.18 ± 5.81

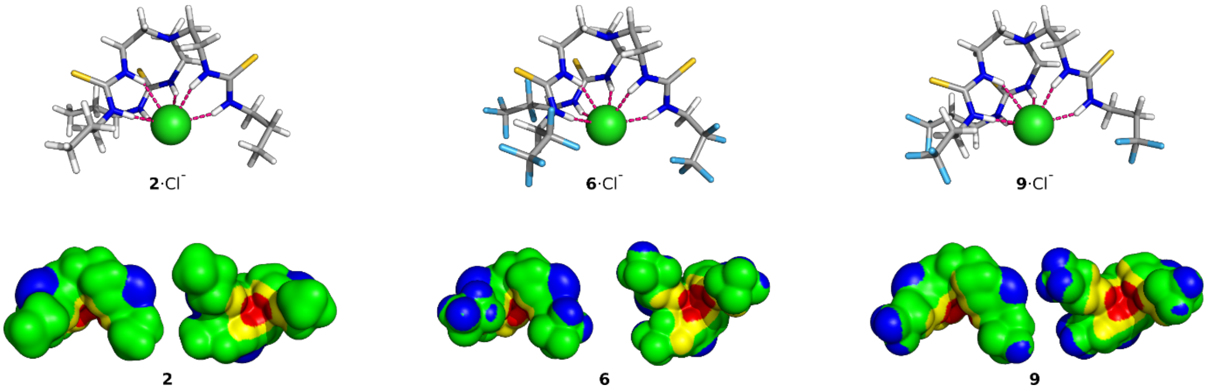

Figure S4.23 Top: DFT optimised structures of complexes **2**·Cl$^-$, **6**·Cl$^-$, and **9**·Cl$^-$. The hydrogen, carbon, sulfur, nitrogen, fluorine atoms and chloride anion are shown in white, grey, yellow, blue and cyan sticks, respectively, while the anion is shown as a green sphere. The N–H···Cl$^-$ hydrogen bonds are drawn as pink dashed lines. Bottom: Distribution of the electrostatic potential mapped on the 0.001 electrons Bohr^{-3} isodensity surface of **2**, **6**, and **9** in side and bottom views. The colour scales range from blue to red, in kcal mol^{-1}, as follows: blue – lower than –5.0; green – between –5.0 and 30.0; yellow – between 30.0 and 65.0; and red – greater than 65.0.

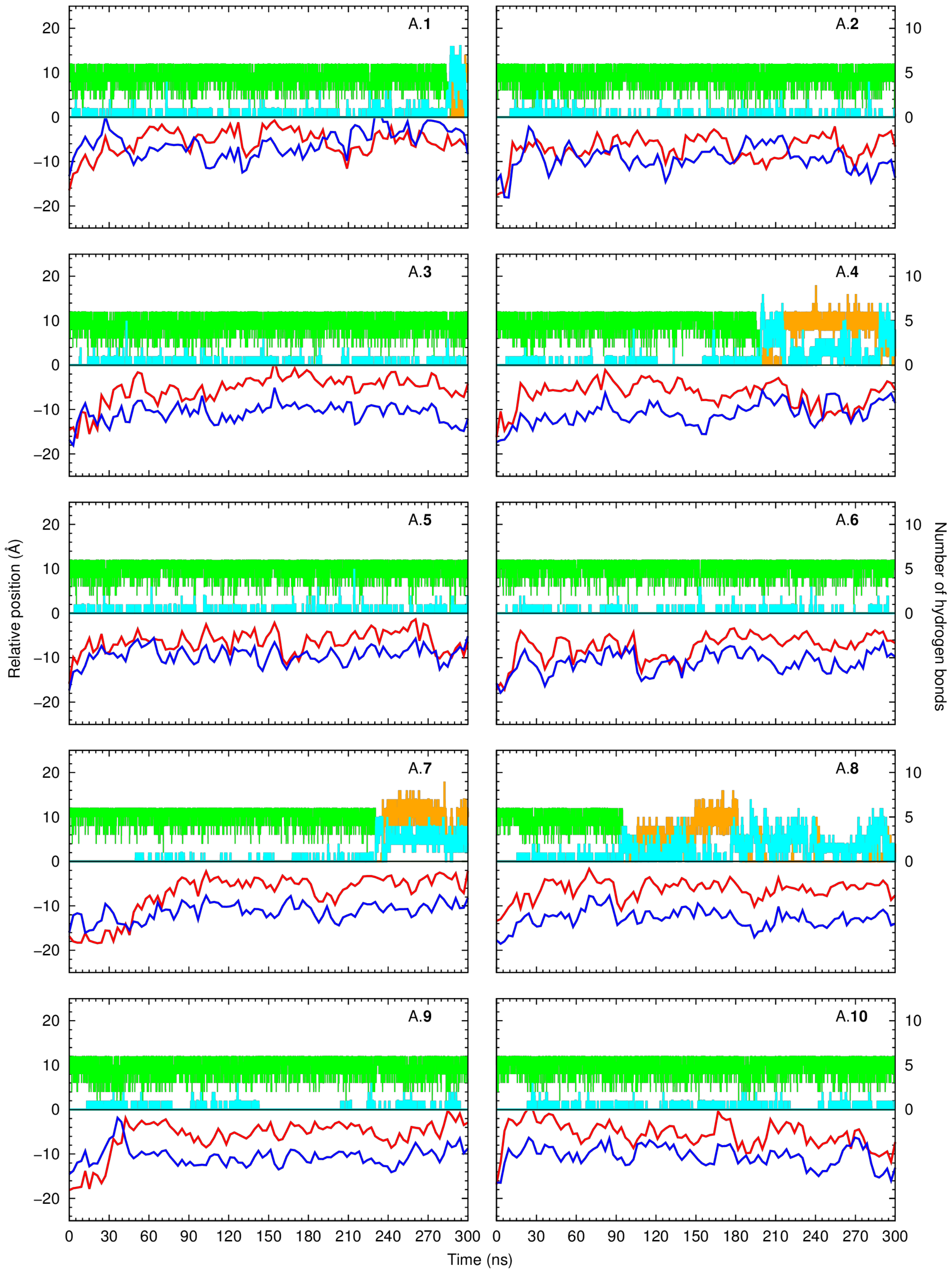

Figure S4.24 Evolution of the $N_{tren}\cdots P_{int}$ (red line) and $C_{ter}\cdots P_{int}$ (blue line) distances throughout 300 ns of the MD replicates of **1** - **10**·Cl⁻ in scenario A (data were smoothed with Bézier curves). The evolution of the number of N−H···Cl⁻ hydrogen bonds is also plotted as a green line, while the hydrogen bonds with the water molecules or phosphate head groups are shown in cyan and orange, respectively. The water/lipid interface is represented as a black line at z = 0 Å. Each MD simulation is identified with the initial scenario and tripodal molecule studied.

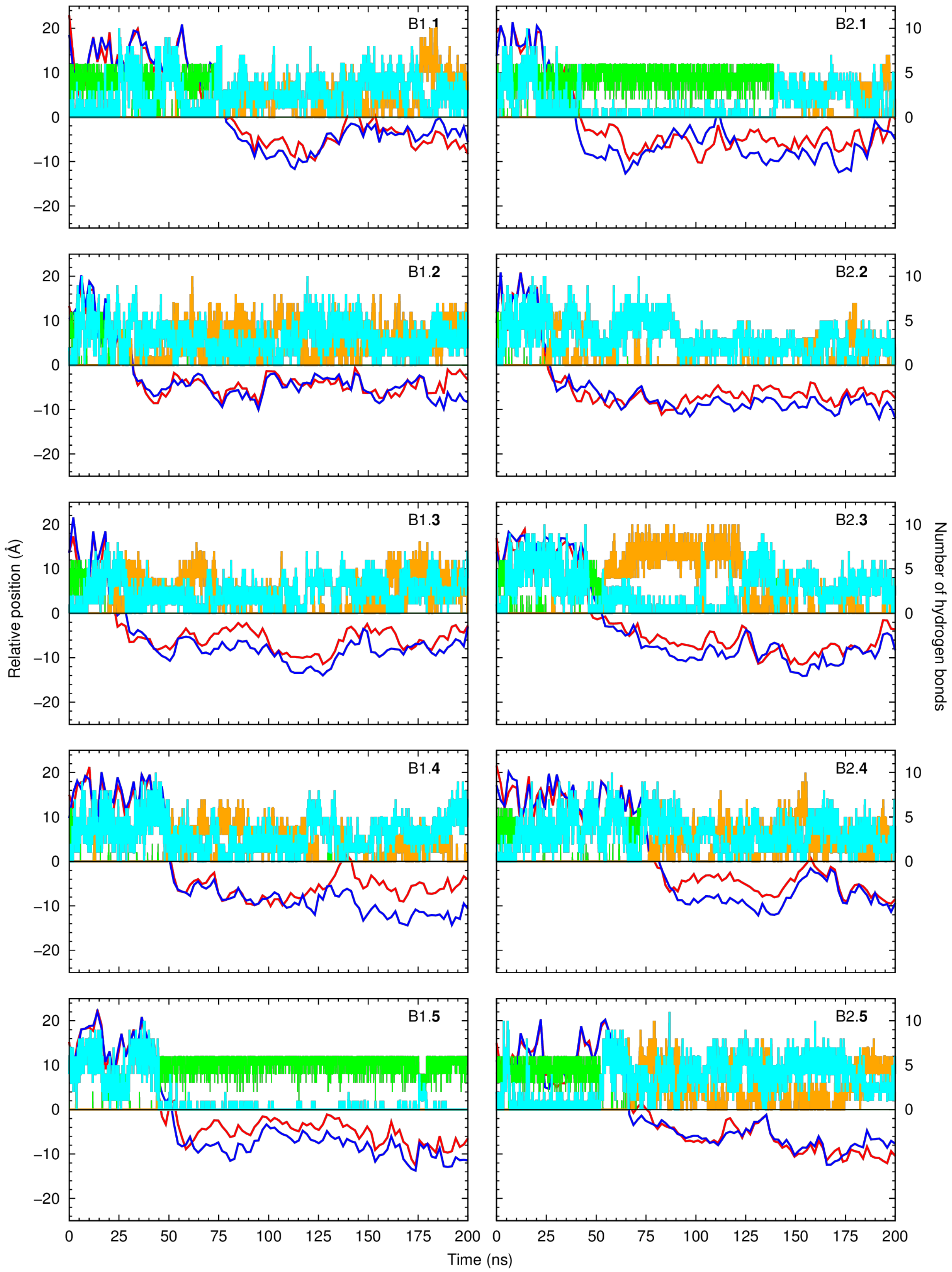

Figure S4.25 Evolution of the $N_{tren}\cdots P_{int}$ (red line) and $C_{ter}\cdots P_{int}$ (blue line) distances throughout 200 ns of the MD replicates of **1** - **5**·Cl^- in scenario B (data were smoothed with Bézier curves). The evolution of the number of N−H···Cl^- hydrogen bonds is also plotted as a green line, while the hydrogen bonds with the water molecules or phosphate head groups are shown in cyan and orange, respectively. The water/lipid interface is represented as a black line at $z = 0$ Å. Each MD simulation is identified with the initial scenario, MD run number and tripodal molecule studied.

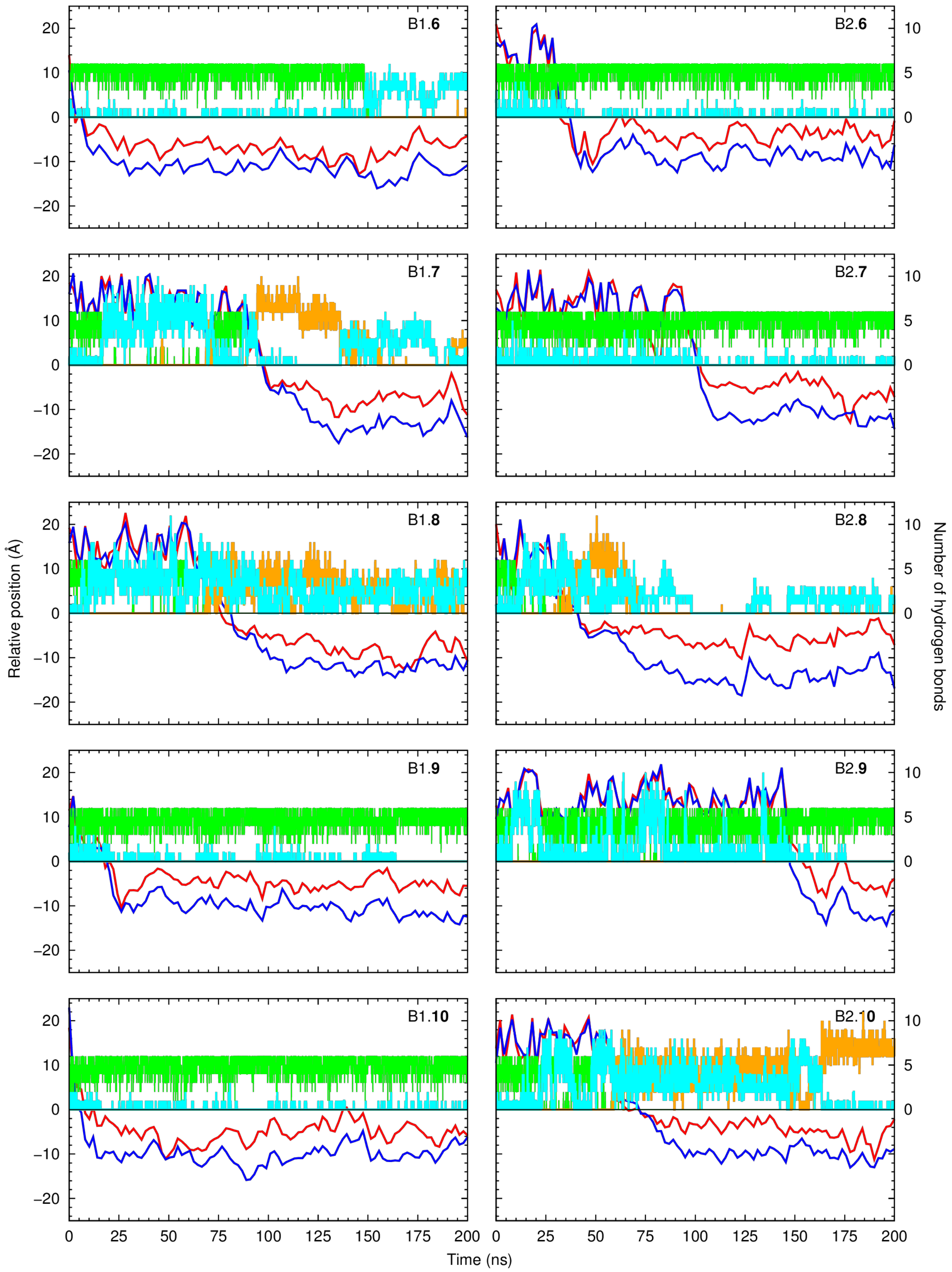

Figure S4.26 Evolution of the N$_{tren}$···P$_{int}$ (red line) and C$_{ter}$···P$_{int}$ (blue line) distances throughout 200 ns of the MD replicates of **6** - **10**·Cl⁻ in scenario B (data were smoothed with Bézier curves). The evolution of the number of N−H···Cl⁻ hydrogen bonds is also plotted as a green line, while the hydrogen bonds with the water molecules or phosphate head groups are shown in cyan and orange, respectively. The water/lipid interface is represented as a black line at z = 0 Å. Each MD simulation is identified with the initial scenario, MD run number and tripodal molecule studied.

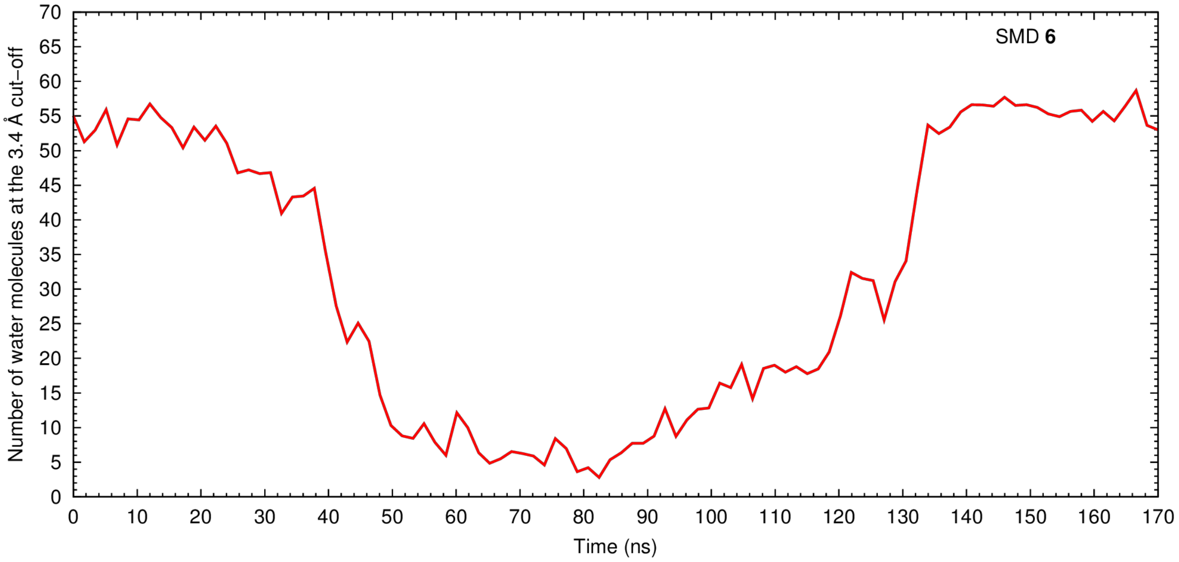

Figure S4.27 Variation of the number of water molecules within the solvation shell defined by a cut-off of 3.4 Å from **6**, in the SMD simulation from one side of the POPC bilayer to the opposite side. Data were smoothed using Bézier curves.

The hydration of the free compounds and their chloride complexes was assessed throughout the last 50 ns of the US MD simulations and is presented in Fig. S4.28. The translocation of the transporters is accompanied by a drift of water molecules, whose number markedly decreases as the transporters are located closer to the bilayer core.

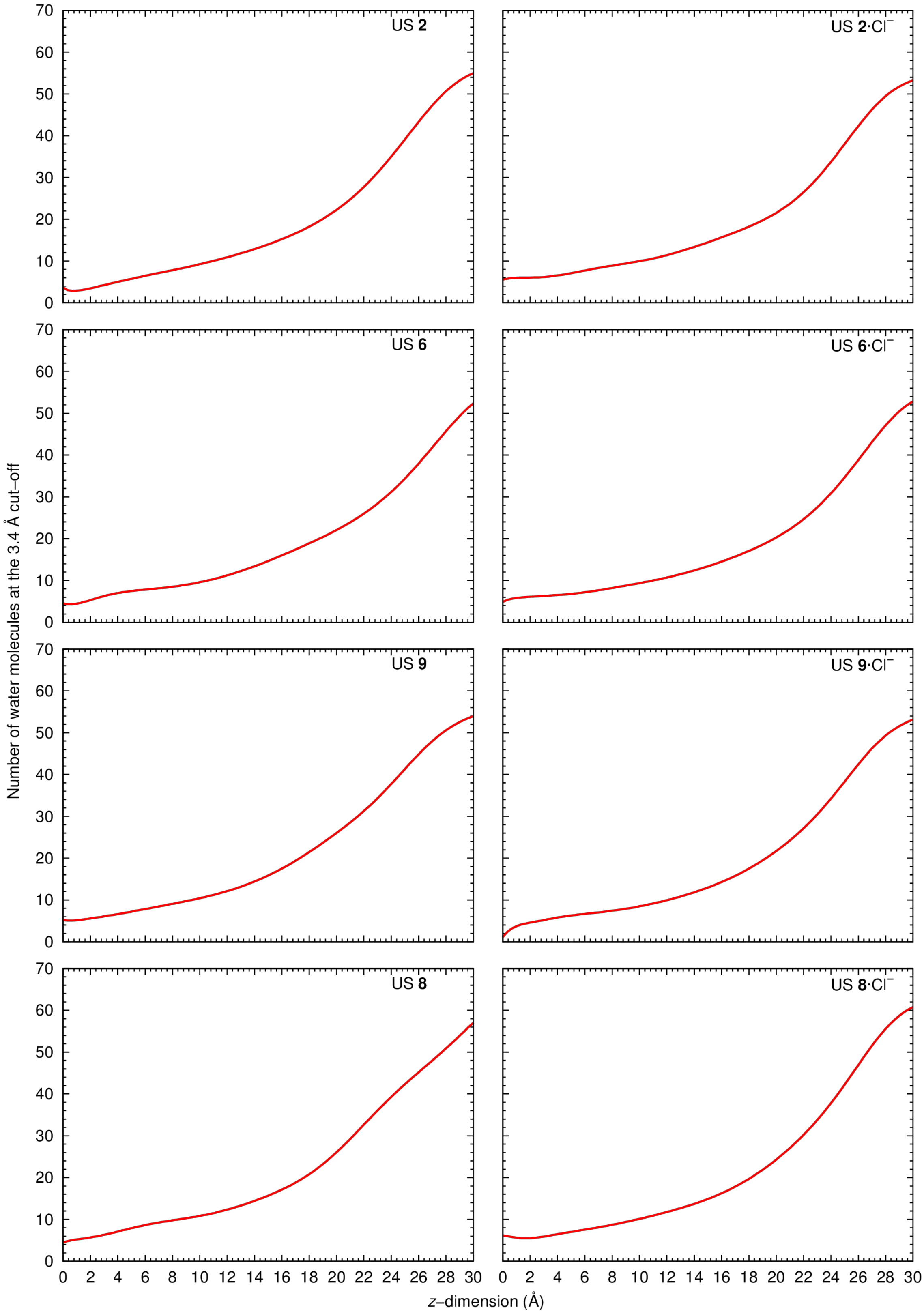

Figure S4.28 Variation of the number of water molecules within the solvation shell defined by a cut-off of 3.4 Å from **2**, **6**, **9** and **8**, during the last 50 ns of the US MD simulations of the free transporters (left) or their chloride complexes (right), between *z* = 0 Å and *z* = 30 Å. Data were smoothed using Bézier curves.

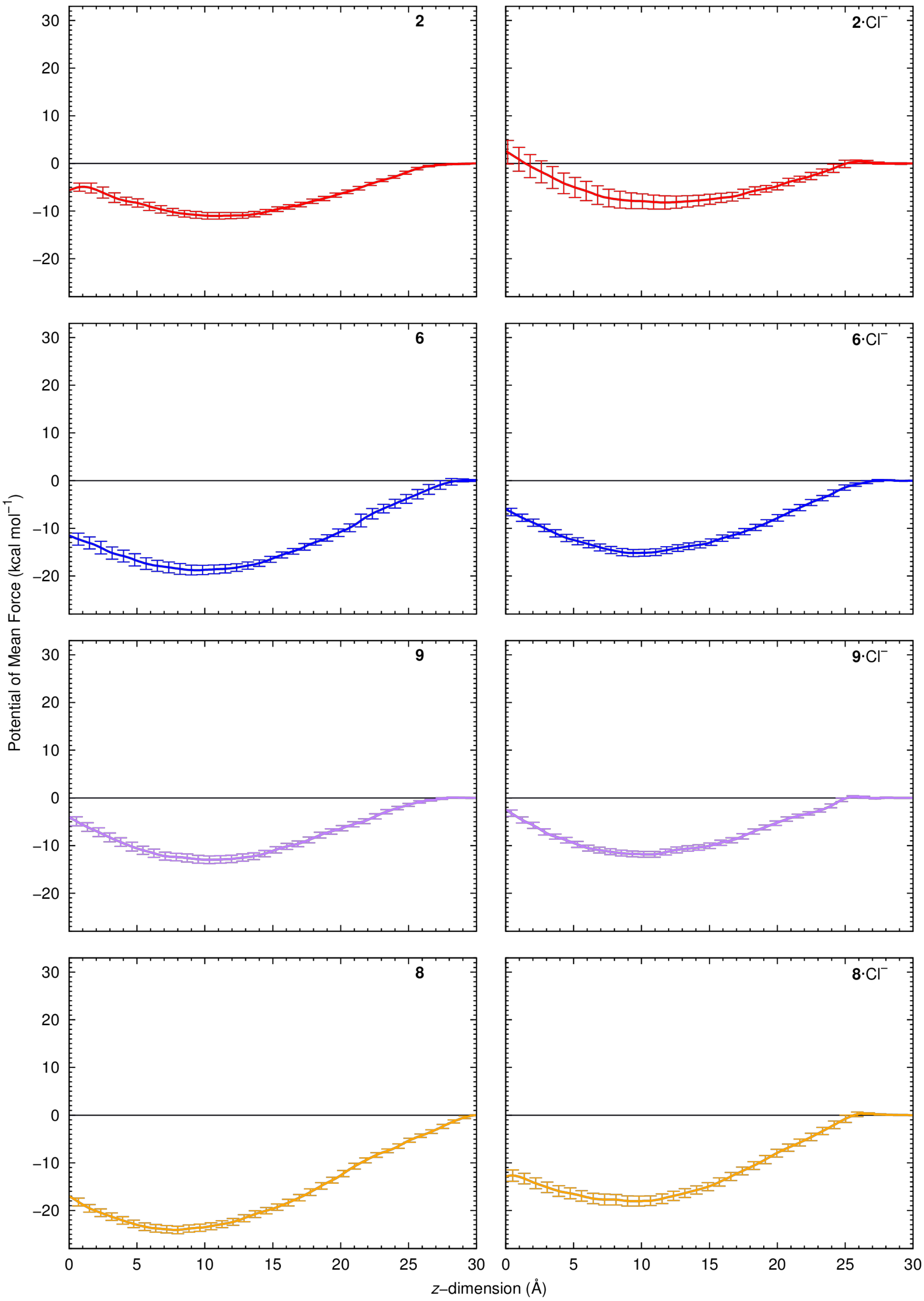

Figure S4.29 PMF as a function of the tren thiourea derivatives' distance to the membrane centre of mass (Z = 0 Å), for the free **2**, **6**, **9** and **8** as well as their chloride complexes. The error bars correspond to the bootstrap errors calculated from 100 random data sets with the same size and are upscaled 5 times.

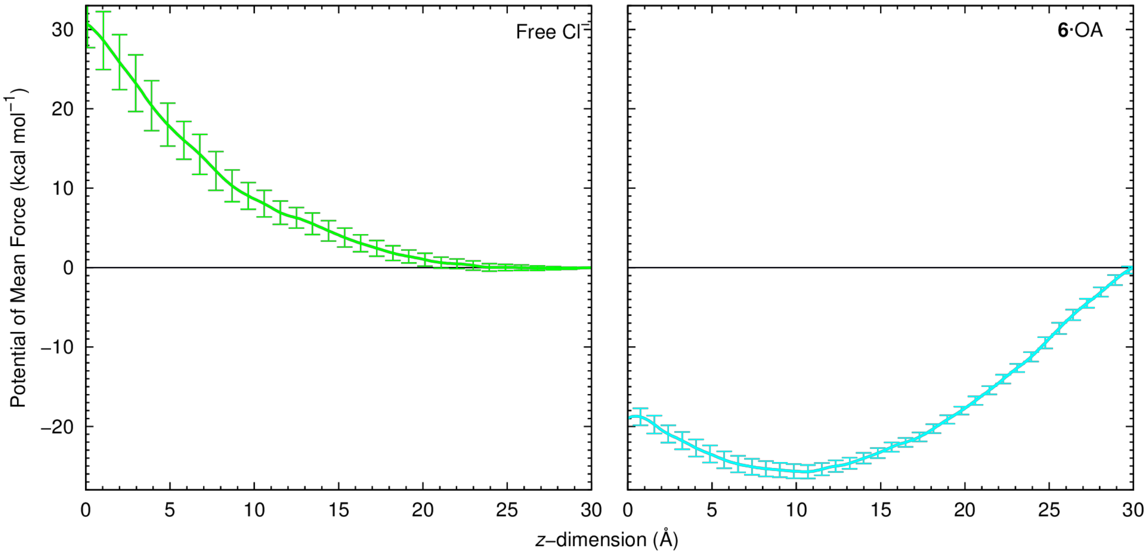

Figure S4.30 PMF as a function of the distance to the membrane centre of mass (Z = 0 Å), for the free chloride and **6**·OA. The error bars correspond to the bootstrap errors calculated from 100 random data sets with the same size and are upscaled 5 times.

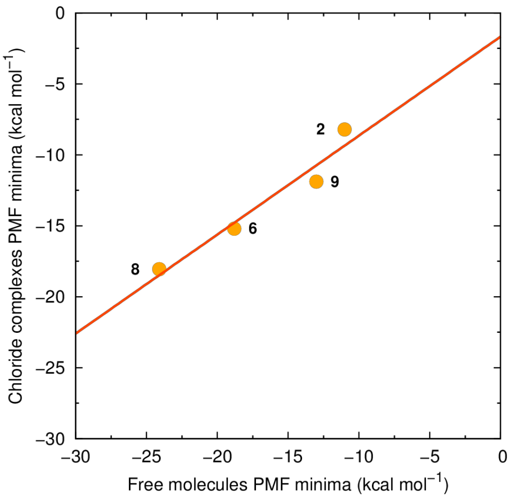

Figure S4.31 Comparison of the PMF minima between the free tripodal molecules **2**, **6**, **8** and **9** and their chloride complexes (R^2 = 0.94). Compound numbers are shown next to the data points.

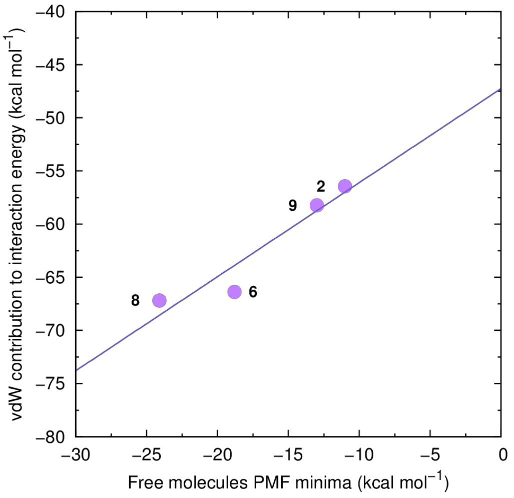

Figure S4.32 Van der Waals contribution to the total Molecular Mechanics interaction energy between the free tripodal transporters **2**, **6**, **8** and **9** as a function of their calculated PMF minima (R^2 = 0.90). Compound numbers are shown next to the data points.

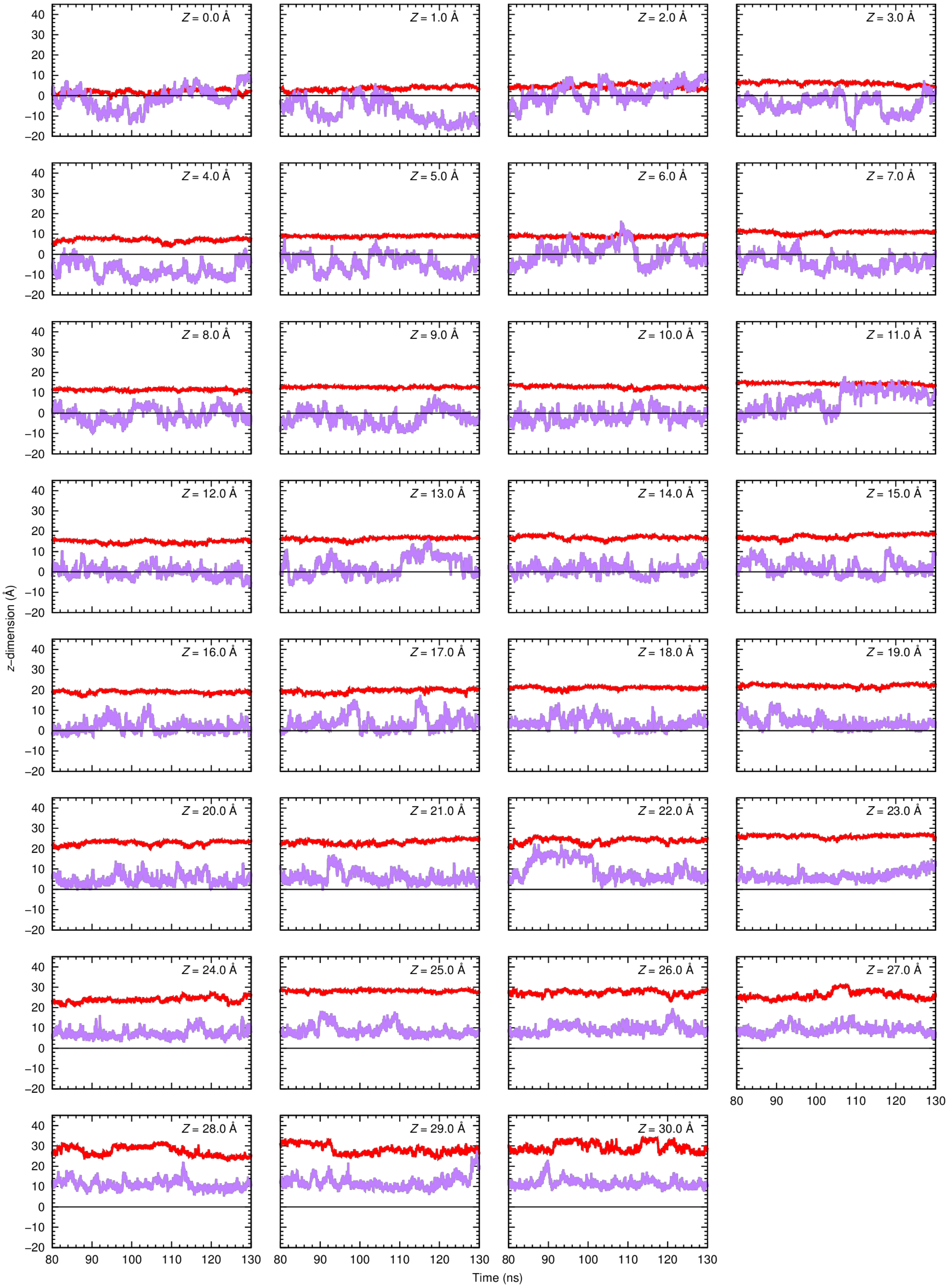

Figure S4.33 Variation of the N_{tren} (red) and OA_{CH3} (purple) referential points in **6** and OA along the final 50 ns of the 31 US MD windows. The centre of the bilayer is represented as a black line at z = 0 Å.

S4.4.1 Convergence of the Free Energy Profiles

The convergence of the PMF profiles was ascertained by assessing the data in sequential 20 ns intervals, until it was apparent that the curves overlapped continuously (Figure S4.34). For instance, for **6**·OA, longer simulation windows were needed, due to the flexibility of oleic acid.

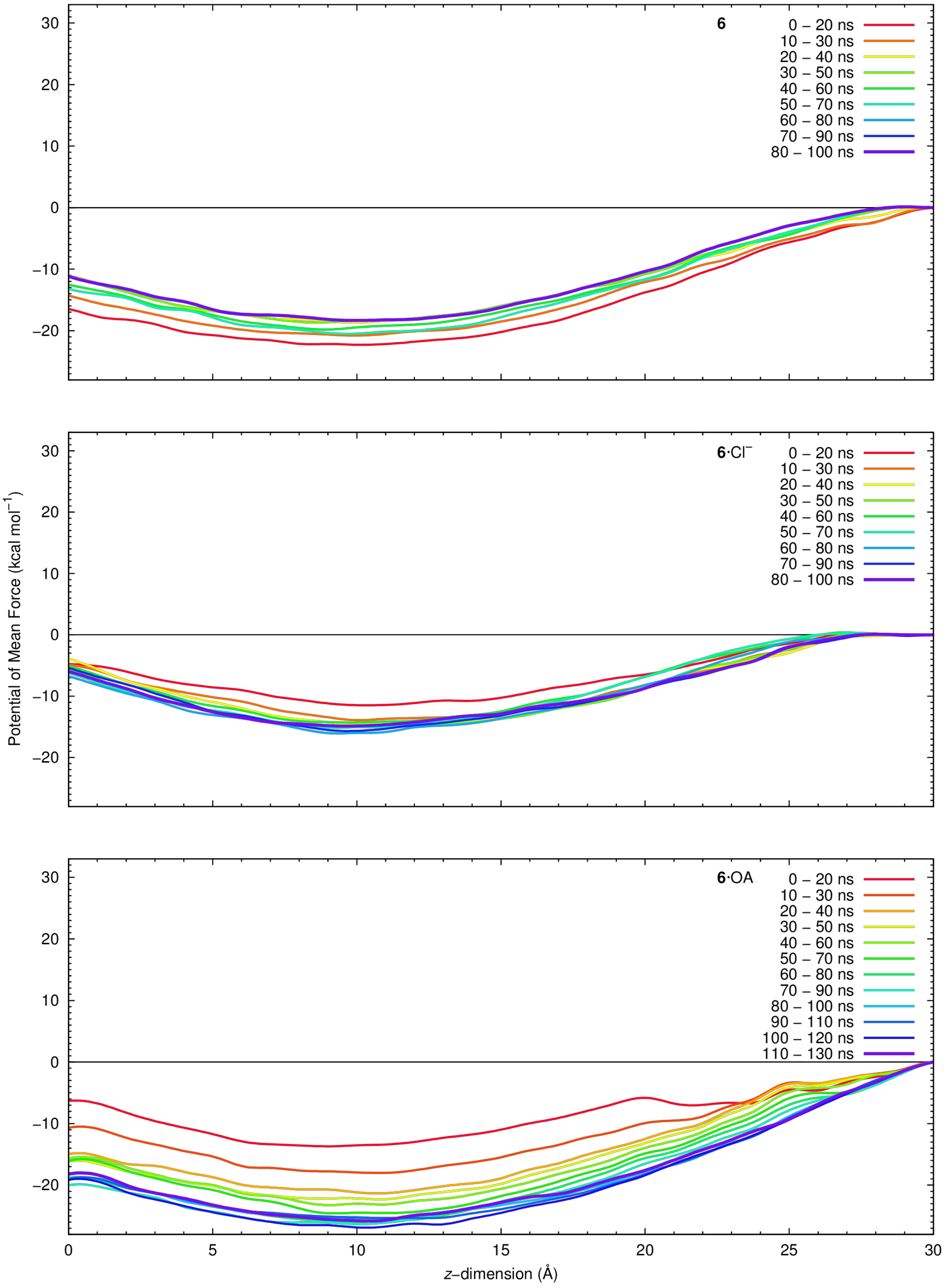

Figure S4.34 The equilibration and convergence of the umbrella sampling simulation windows were assessed in 20 ns intervals (coloured according to time period), taken through the trajectory and used to calculate the PMF of **6**, **6**·Cl^- or **6**·OA. The PMF curves for **6** and **6**·Cl^- converged within the initial 50 ns. However, for **6**·OA extended simulation time was needed to achieve the sampling time of 50 ns.

S4.5 Anion Transport in Cells

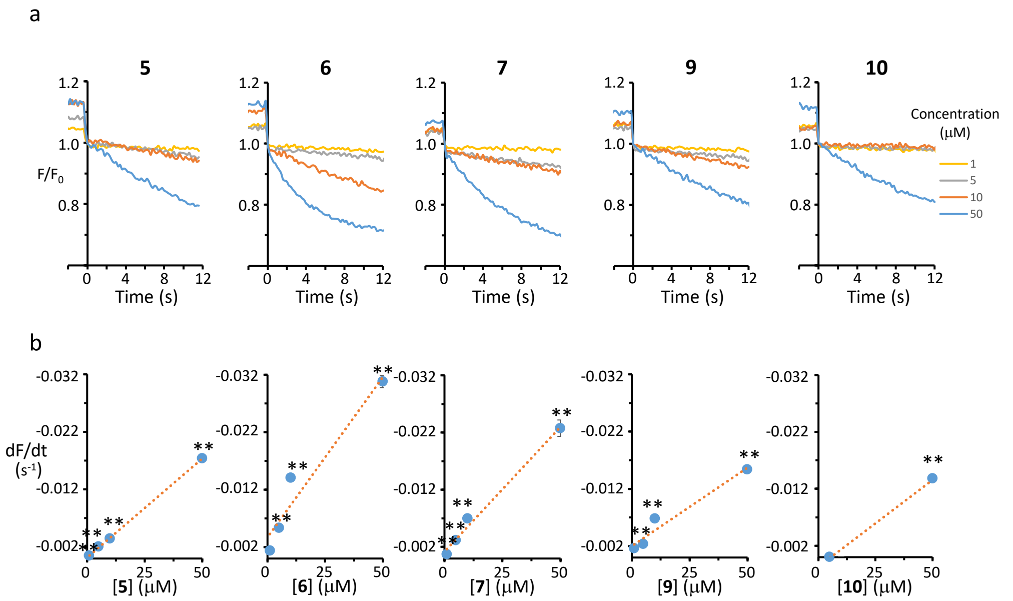

Figure S4.35 Concentration-dependence of anion transport by anionophores. (a) Representative time courses of cell fluorescence from YFP-FRT cells treated with increasing concentrations of the indicated anionophores in DMSO (0.1 or 0.5% v v^{-1}). Cell fluorescence values were normalised to the fluorescence intensity before NaI (100 mM) addition at t = 0 s. (b) Relationship between anionophore concentration and anion transport for compounds **5 - 7**, **9** and **10** in YFP-FRT cells. Fluorescence quenching by the anionophore vehicle (1 – 10 μM, 0.1% v v^{-1} DMSO; 50 μM, 0.5% v v^{-1} DMSO) was subtracted from each test measurement. Data are means ± SEM (n = 16 – 32 from at least four independent experiments); ** P < 0.01 vs. control. The dotted lines show the fit of first-order regression functions to mean data.

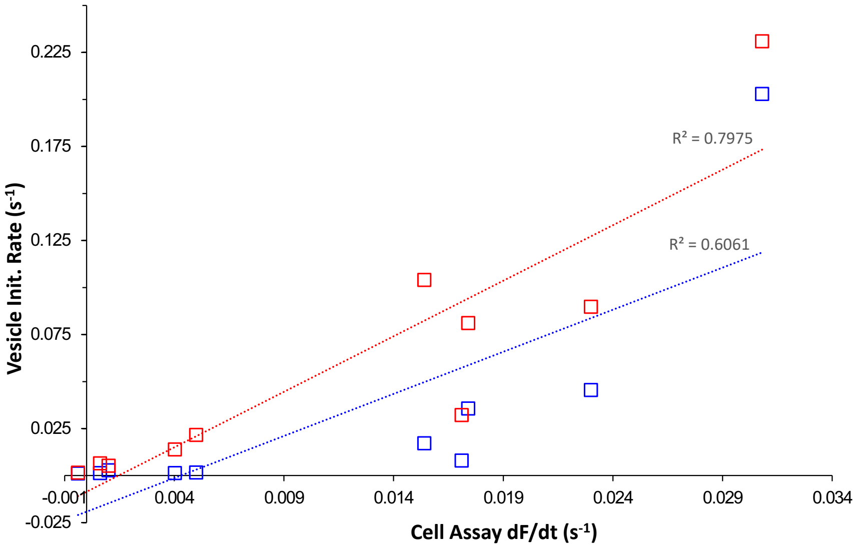

Figure S4.36 Correlation of anion transport activity in cells and vesicles. Symbols show the fastest rates of anion transport for compounds **1** – **10** determined in the absence (blue) and presence (red) of protonophores. For analysis, the fastest rate of transport in either Gr or OA was used. The dotted lines show the fit of first-order regression functions to the data (absence of protonophore [blue], r^2 = 0.6061; presence of protonophore [red], r^2 = 0.7975)

S4.6 **Crystal Data**

Table S4.4 Crystal data and structure refinement details for **3**·TBACl complex (CCDC 1859132).

Empirical formula	$C_{37}H_{81}ClN_8S_3$	
	$C_{21}H_{45}N_7S_3$, $C_{16}H_{36}N$, Cl	
Formula weight	769.73	
Temperature	120(2) K	
Wavelength	0.71073 Å	
Crystal system	Triclinic	
Space group	*P*–1	
Unit cell dimensions	a = 13.6678(11) Å	α = 85.095(5)°
	b = 15.9682(16) Å	β = 74.096(6)°
	c = 22.901(2) Å	γ = 76.589(6)°
Volume	4674.5(7) Å^3	
Z	4 (Z′ = 2)	
Density (calculated)	1.094 Mg / m^3	
Absorption coefficient	0.249 mm^{-1}	
F(000)	1696	
Crystal	Plate; Colourless	
Crystal size	0.30 × 0.18 × 0.03 mm^3	
θ range for data collection	2.92 – 25.03°	
Index ranges	$-16 \le h \le 16$, $-18 \le k \le 18$, $-27 \le l \le 27$	
Reflections collected	50328	
Independent reflections	15862 [R_{int} = 0.1351]	
Completeness to θ = 25.03°	96.0 %	
Absorption correction	Semi–empirical from equivalents	
Max. and min. transmission	0.9926 and 0.9291	
Refinement method	Full-matrix least-squares on F^2	
Data / restraints / parameters	15862 / 63 / 911	
Goodness-of-fit on F^2	1.065	
Final *R* indices [$F^2 > 2\sigma(F^2)$]	$R1$ = 0.1510, $wR2$ = 0.2726	
R indices (all data)	$R1$ = 0.2553, $wR2$ = 0.3310	
Largest diff. peak and hole	0.665 and –0.403 e Å^{-3}	

Diffractometer: *Nonius KappaCCD* area detector (ϕ scans and ω scans to fill asymmetric unit). **Cell determination:** DirAx[34] **Data collection:** Collect (Collect: Data collection software, R. Hooft, Nonius B.V., 1998). **Data reduction and cell refinement**: *Denzo*[35]. **Absorption correction**: Sheldrick, G. M. SADABS - Bruker Nonius area detector scaling and absorption correction - V2.10 **Structure solution**: *SHELXS97*[36]. **Structure refinement**: *SHELXL97*[37].

Special details: All hydrogen atoms were placed in idealised positions and refined using a riding model. One of the receptor molecule arms is modelled as disordered over 2 positions. Thermal parameter and geometric restraints were applied. This disorder is responsible for a close contact "H66C..H17C = 1.98 A" and a large Hirshfeld difference "C65--C66 = 0.26 A". It was evident that the data quality did not permit finer detail disorder to be modelled.

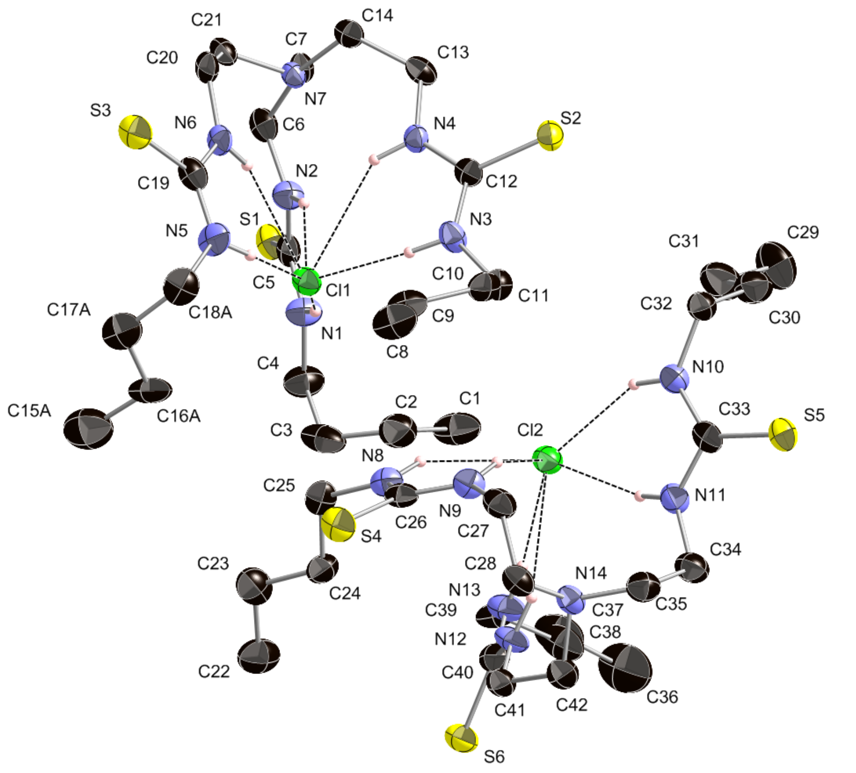

Figure S4.37 Crystal structure of **3**·Cl^- complex. Thermal ellipsoids are shown at the 35% probability level. TBA^+ counterion and disordered atoms have been omitted for clarity.

S5 References

1. X. Wu, L. W. Judd, E. N. W. Howe, A. M. Withecombe, V. Soto-Cerrato, H. Li, N. Busschaert, H. Valkenier, R. Pérez-Tomás, D. N. Sheppard, Y.-B. Jiang, A. P. Davis and P. A. Gale, *Chem*, 2016, **1**, 127-146.
2. P. G. Young, J. K. Clegg, M. Bhadbhade and K. A. Jolliffe, *Chem. Commun.*, 2011, **47**, 463-465.
3. N. Busschaert, P. A. Gale, C. J. E. Haynes, M. E. Light, S. J. Moore, C. C. Tong, J. T. Davis and J. William A. Harrell, *Chem. Commun.*, 2010, **46**, 6252-6254.
4. BindFit v0.5, http://app.supramolecular.org/bindfit/, (accessed June 2017).
5. P. Thordarson, *Chem. Soc. Rev.*, 2011, **40**, 1305-1323.
6. M. J. Frisch, G. W. Trucks, H. B. Schlegel, G. E. Scuseria, M. A. Robb, J. R. Cheeseman, G. Scalmani, V. Barone, B. Mennucci, G. A. Petersson, H. Nakatsuji, M. Caricato, X. Li, H. P. Hratchian, A. F. Izmaylov, J. Bloino, G. Zheng, J. L. Sonnenberg, M. Hada, M. Ehara, K. Toyota, R. Fukuda, J. Hasegawa, M. Ishida, T. Nakajima, Y. Honda, O. Kitao, H. Nakai, T. Vreven, J. J. A. Montgomery, J. E. Peralta, F. Ogliaro, M. Bearpark, J. J. Heyd, E. Brothers, K. N. Kudin, V. N. Staroverov, R. Kobayashi, J. Normand, K. Raghavachari, A. Rendell, J. C. Burant, S. S. Iyengar, J. Tomasi, M. Cossi, N. Rega, J. M. Millam, M. Klene, J. E. Knox, J. B. Cross, V. Bakken, C. Adamo, J. Jaramillo, R. Gomperts, R. E. Stratmann, O. Yazyev, A. J. Austin, R. Cammi, C. Pomelli, J. W. Ochterski, R. L. Martin, K. Morokuma, V. G. Zakrzewski, G. A. Voth, P. Salvador, J. J. Dannenberg, S. Dapprich, A. D. Daniels, Ö. Farkas, J. B. Foresman, J. V. Ortiz, J. Cioslowski and D. J. Fox, *Gaussian 09 Revision A1*, Gaussian, Inc., Wallingford, CT, 2009.
7. T. Lu and F. Chen, *J. Comput. Chem.*, 2012, **33**, 580-592.
8. D. A. Case, R. M. Betz, W. Botello-Smith, D. S. Cerutti, I. T. E. Cheatham, T. A. Darden, R. E. Duke, T. J. Giese, H. Gohlke, A. W. Goetz, N. Homeyer, S. Izadi, P. Janowski, J. Kaus, A. Kovalenko, T. S. Lee, S. LeGrand, P. Li, C. Lin, T. Luchko, R. Luo, B. Madej, D. Mermelstein, K. M. Merz, G. Monard, H. Nguyen, H. T. Nguyen, I. Omelyan, A. Onufriev, D. R. Roe, A. Roitberg, C. Sagui, C. L. Simmerling, J. Swails, R. C. Walker, J. Wang, R. M. Wolf, X. Wu, L. Xiao, D. M. York and P. A. Kollman, *AMBER* 2016, University of California, San Francisco, 2016.
9. A. W. Gotz, M. J. Williamson, D. Xu, D. Poole, S. Le Grand and R. C. Walker, *J. Chem. Theory Comput*, 2012, **8**, 1542-1555.
10. R. Salomon-Ferrer, A. W. Gotz, D. Poole, S. Le Grand and R. C. Walker, *J. Chem. Theory Comput.*, 2013, **9**, 3878-3888.
11. S. Le Grand, A. W. Götz and R. C. Walker, *Comput. Phys. Commun.*, 2013, **184**, 374-380.
12. C. J. Dickson, B. D. Madej, A. A. Skjevik, R. M. Betz, K. Teigen, I. R. Gould and R. C. Walker, *J. Chem. Theory Comput.*, 2014, **10**, 865-879.
13. J. Wang, R. M. Wolf, J. W. Caldwell, P. A. Kollman and D. A. Case, *J. Comput. Chem.*, 2004, **25**, 1157-1174.
14. J. Wang, R. M. Wolf, J. W. Caldwell, P. A. Kollman and D. A. Case, *J. Comput. Chem.*, 2005, **26**, 114-114.
15. C. I. Bayly, P. Cieplak, W. D. Cornell and P. A. Kollman, *J. Phys. Chem.*, 1993, **97**, 10269-10280.
16. J. Wang, W. Wang, P. A. Kollman and D. A. Case, *J. Mol. Graph Model*, 2006, **25**, 247-260.
17. E. F. Pettersen, T. D. Goddard, C. C. Huang, G. S. Couch, D. M. Greenblatt, E. C. Meng and T. E. Ferrin, *J. Comput. Chem.*, 2004, **25**, 1605-1612.
18. L. Martinez, R. Andrade, E. G. Birgin and J. M. Martinez, *J Comput Chem*, 2009, **30**, 2157-2164.
19. W. L. Jorgensen, J. Chandrasekhar, J. D. Madura, R. W. Impey and M. L. Klein, *J. Chem. Phys.*, 1983, **79**, 926-935.
20. P. Li, L. F. Song and K. M. Merz, Jr., *J. Chem. Theory Comput.*, 2015, **11**, 1645-1657.
21. T. Darden, D. York and L. Pedersen, *J. Chem. Phys.*, 1993, **98**, 10089-10092.
22. R. J. Loncharich, B. R. Brooks and R. W. Pastor, *Biopolymers*, 1992, **32**, 523-535.
23. H. J. C. Berendsen, J. P. M. Postma, W. F. Vangunsteren, A. Dinola and J. R. Haak, *J. Chem. Phys.*, 1984, **81**, 3684-3690.
24. J.-P. Ryckaert, G. Ciccotti and H. J. C. Berendsen, *J. Comput. Phys.*, 1977, **23**, 327-341.
25. D. R. Roe and T. E. Cheatham, 3rd, *J. Chem. Theory Comput.*, 2013, **9**, 3084-3095.

26. T. S. Lee, B. K. Radak, A. Pabis and D. M. York, *J. Chem. Theory Comput.*, 2013, **9**, 153-164.
27. T. S. Lee, B. K. Radak, M. Huang, K. Y. Wong and D. M. York, *J. Chem. Theory Comput.*, 2014, **10**, 24-34.
28. A. S. Verkman and L. J. V. Galietta, *Nat. Rev. Drug Discov.*, 2009, **8**, 153-171.
29. L. V. J. Galietta, S. Jayaraman and A. S. Verkman, *Am. J. Physiol. Cell Physiol.*, 2001, **281**, C1734-C1742.
30. H. Li, H. Valkenier, L. W. Judd, P. R. Brotherhood, S. Hussain, J. A. Cooper, O. Jurček, H. A. Sparkes, D. N. Sheppard and A. P. Davis, *Nat. Chem.*, 2016, **8**, 24-32.
31. OECD, *OECD Guidelines for the Testing of Chemicals, Section 1*, 2004, DOI: 10.1787/9789264069824-en.
32. A. Berthod and S. Carda-Broch, *J Chromatogr*, 2004, **1037**, 3-14.
33. M. J. Spooner and P. A. Gale, *Chem. Commun.*, 2015, **51**, 4883-4886.
34. A. Duisenberg, *J. Appl. Crystallogr.*, 1992, **25**, 92-96.
35. Z. Otwinowski and W. Minor, in *Method. Enzymol.*, eds. C. W. Carter, Jr. and R. M. Sweet, Academic Press, 1997, vol. 276, pp. 307-326.
36. G. Sheldrick, *Acta Crystallogr. A*, 1990, **46**, 467-473.
37. G. M. Sheldrick, *Program for crystal-structure refinement. University of Gottingen, Germany.*, 1997.
